# Cannabis Virome Reconstruction and Antiviral RNAi Characterization through Small RNA Sequencing

**DOI:** 10.3390/plants12233925

**Published:** 2023-11-21

**Authors:** Niccolo’ Miotti, Natalia Sukhikh, Nathalie Laboureau, Paola Casati, Mikhail M. Pooggin

**Affiliations:** 1 Department of Agricultural and Food Sciences—Production, Landscape, Agroenergy, University of Milan, Via Celoria 2, 20133 Milan, Italy; niccolo.miotti@unimi.it (N.M.); paola.casati@unimi.it (P.C.); 2 PHIM Plant Health Institute, University Montpellier, CIRAD, INRAE, IRD, Institute Agro, 34398 Montpellier, France; sukhikh.natalie@gmail.com (N.S.); nathalie.laboureau@cirad.fr (N.L.)

**Keywords:** *Cannabis sativa*, hemp, virus, *Bromoviridae*, *Mitoviridae*, *Partitiviridae*, small RNA, RNA interference

## Abstract

Viral infections pose an emerging threat to hemp (*Cannabis sativa*) cultivation. We used Illumina small (s)RNA sequencing for virome reconstruction and characterization of antiviral RNA interference (RNAi) in monoecious and dioecious hemp varieties, which exhibited different virus-like symptoms. Through de novo and reference-based sRNA assembly, we identified and reconstructed Cannabis cryptic virus (family *Partitiviridae*), *Cannabis sativa* mitovirus 1 (*Mitoviridae*) and Grapevine line pattern virus (*Bromoviridae*) as well as a novel virus tentatively classified into *Partitiviridae*. Members of both *Partitiviridae* and *Bromoviridae* were targeted by antiviral RNAi, generating 21 nt and, less abundant, 22 nt sRNAs from both strands of the entire virus genome, suggesting the involvement of Dicer-like (DCL) 4 and DCL2 in viral sRNA biogenesis, respectively. Mitovirus sRNAs represented predominantly the positive-sense strand and had a wider size range, with the 21 nt class being most abundant on both strands. For all viruses, 21 and 22 nt sRNAs had predominantly 5′-terminal uridine or cytosine, suggesting their binding to antiviral Argonaute (AGO) 1 and AGO5, respectively. As no clear association of any virus with symptoms was observed, further studies should clarify if these viruses individually or in combination can cause hemp diseases.

## 1. Introduction

Hemp (*Cannabis sativa* L.) is currently undergoing rediscovery in various economical fields, from industrial to medicinal [1,2]. The increase in cultivated areas is creating new challenges in managing hemp diseases. Fungal and bacterial pathogens of hemp have been extensively studied, and effective management strategies are well-known [3]. However, viral infections are emerging as a significant threat to hemp cultivation [4]. Indeed, new viruses and a viroid have recently been reported as capable of infecting *C. sativa* and significantly reducing the yield [5,6,7,8,9]. Among other approaches for virus identification, Illumina transcriptome sequencing (Illumina, Inc., San Diego, CA, USA), followed by the de novo assembly of sequencing reads, was exploited to study *C. sativa* virome, revealing mixed infections of known and previously unreported viruses [10].

RNA interference (RNAi) is an evolutionarily conserved mechanism that regulates gene expression and defends against invasive nucleic acids such as transposons, transgenes and viruses in most eukaryotes. In plants, RNAi is directed by 21, 22 and 24 nt small (s)RNAs which are generated by Dicer-like (DCL) family proteins from longer double-stranded (ds)RNA precursors and then get associated with Argonaute (AGO) family proteins that sort sRNAs by size and 5′-nucleotide identity to create RNA-induced silencing complexes repressing target genes post-transcriptionally and/or transcriptionally in a sequence-specific manner [11,12]. Based on genetic and biochemical evidence from model plants such as *Arabidopsis thaliana* and deep sRNA sequencing studies of various cultivated and non-cultivated land plants, all four types of DCLs are involved in antiviral defence. They generate 21 nt (DCL4, DCL1), 22 nt (DCL2) and 24 nt (DCL3) small interfering (si)RNAs from the entire genomes of RNA and DNA viruses as well as viral satellites and viroids [13,14,15]. Although no information is available on *C. sativa* DCLs and AGOs, the RNAi machinery is well conserved in land plants (reviewed in [15]), and, therefore, we assume that the main types/clades of DCLs (homologs of the *A. thaliana* DCL1, DCL2, DCL3 and DCL4) and AGOs (homologs of the *A. thaliana* AGO1, AGO2, AGO4 and AGO5 clades) are likely encoded in the genome of *C. sativa* that belongs, together with *A. thaliana,* to dicot plants.

As virus-derived siRNAs densely cover both strands of the viral genome as tiling arrays without gaps, the deep sequencing and de novo assembly of sRNAs allow for the reconstruction of complete genomes of known and unknown viruses and viroids [14], while the bioinformatics analysis of size, polarity, 5′-nucleotide identity and hotspot profiles of viral and host sRNAs allows for the characterization of the plant RNAi machinery [15]. In this work, we made use of sRNA sequencing and bioinformatics for the identification and reconstruction of virome components and the characterization of antiviral RNAi of *C. sativa* plants cultivated for both cannabinoid and fiber/seed production, which exhibited different virus-like symptoms. To our knowledge, this is the first characterization of antiviral RNAi in hemp plants. Previously, sRNA sequencing was undertaken, along with transcriptome and degradome analysis, to study the regulation of key genes involved in cannabinoid biosynthesis in hemp plants [16].

## 2. Results and Discussion

### 2.1. Identification and Genome Reconstruction of Cannabis Virome Components

Nine leaf samples of *Cannabis sativa* collected in June 2022 (Table 1) were subjected to RT-PCR analysis to verify the presence of beet curly top virus (BCTV), lettuce chlorosis virus (LCV) and Cannabis cryptic virus (CanCV) and hop latent viroid (HLVd), previously reported as capable of infecting hemp plants [5,6,7,8,9,10,17,18]. The results were negative for all the tested samples, except ALYU-304 being positive for both RNA-dependent RNA polymerase (RdRP)- and coat protein (CP)-encoding genomic segments of CanCV. The low incidence of the latter virus was somewhat unexpected, as previous studies have reported that CanCV is widely distributed in hemp plants in Europe, particularly in monoecious varieties of the industrial hemp [17,18]. Since among the nine tested samples, only ALYU-304 represented the monoecious hemp (Table 1), dioecious varieties may not (frequently) host CanCV.

We then performed deep sRNA sequencing and bioinformatics analysis to identify and reconstruct virome components in these 9 samples as well as in other 10 samples of monoecious and dioecious hemp plants collected in 2021 (Table 1; Figure S1) and, at the same time, characterize the RNAi machinery of *C. sativa*. Following total RNA extraction and sRNA integrity control using blot hybridization analysis of the evolutionarily conserved plant miRNA (Figure S2), 19 samples (ALYU-297-to-315) were subjected to Illumina sRNA sequencing (Figure S3; Dataset S1), followed by the de novo and reference-based assembly of 15 to 34 nt sRNA reads (as described in Section 3) and Blastn analysis of the sRNA contigs. As a result, we identified and reconstructed genomes of four viruses, including Grapevine line pattern virus (GLPV, genus *Anulavirus*, family *Bromoviridae*), *Cannabis sativa* mitovirus 1 (CasaMV1, genus *Duamitovirus*, family *Mitoviridae*), CanCV (genus *Betapartitivirus*, family *Partitiviridae*) and a novel virus distantly related to Maize-associated partiti-like virus (unclassified, tentative member of *Partitiviridae*) (Table 1). The absence of sRNA contigs representing HLVd, LCV or BCTV confirmed the results of our RT-PCR analysis.

**Figure 1:**
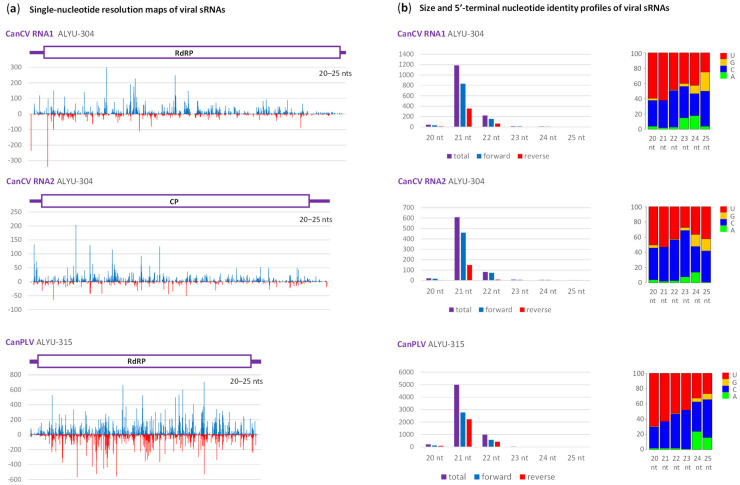
Illumina sequencing analysis of Cannabis cryptic virus (CanCV)- and Cannabis partiti-like virus (CanPLV)-derived small RNAs in hemp plants ALYU-304 and ALYU-315. (**a**) Single-nucleotide resolution maps of 20–25 nt small (s)RNA reads mapped with zero mismatches on the reconstructed genome sequences of CanCV isolate ALYU-304 and CanPLV isolate ALYU-315. Histograms plot the numbers of viral reads at each nucleotide position of two genomic RNAs of CanCV and a single genomic RNA of CanPLV: blue bars above the axis represent positive-sense strand reads starting at each respective position, while red bars below the axis represent complementary strand reads ending at each respective position. The viral genome organization is shown schematically above the histograms, with the ORFs encoding RNA-dependent RNA polymerase (RdRP) and coat protein (CP) of CanCV and putative RdRP of CanPLV boxed. (**b**) Size and 5′-terminal nucleotide identity profiles of viral sRNAs. Counts in reads per million (RPM) of sRNAs derived from positive-sense (forward) and negative-sense (reverse) strands or both strands of the viral genome are plotted as bar graphs. Frequencies of 5′-terminal uridine (U), guanosine (G), cytosine (C) and adenosine (A) are plotted as a percentage of total sRNAs for each of the six size classes (20, 21, 22, 23, 24 and 25 nt).

CanCV was detected only in ALYU-304 and ALYU-306 samples representing two plants of the monoecious hemp variety Felina 32 (Table 1; Dataset S1), thus supporting our RT-PCR results (positive for ALYU-304 and negative for ALYU-308-to-315). The two isolates of the reconstructed CanCV genome comprising 2391 nt RNA1 and 2266 nt RNA2 shared 99.7% (6 single-nucleotide polymorphisms (SNPs)) and 99.6% (8 SNPs) identity (Dataset S2), respectively, and exhibited >99% identity to CanCV isolates “hemp09” (NC_031134.1 and NC_031130.1) and Fedora17 (JN196536.1 and JN196537.1; [17]) for which complete genomes are available and CanCV isolate “CSU” (MT893743.1) for which only RNA1 is available in the NCBI GenBank. The SNPs identified in both isolates did not disrupt the ORFs for RdRP and CP encoded by RNA1 and RNA2, respectively (Figure 1a).

The presence of CanCV was not associated with any particular symptoms: indeed, the CanCV-positive plant ALYU-306 showed symptoms similar to those of CanCV-negative plants ALYU-305 and ALYU-307, whereas the CanCV-positive plant ALYU-304 with four-times-higher counts of viral sRNAs had no detectable symptoms (Table 1). The latter finding confirms a cryptic behaviour of this virus [18], characteristic for plant-infecting members of the family *Partitiviridae* [19] (www.ictv.global/report/partitiviridae; accessed on 1 November 2023). Besides the absence of CanCV in all the dioecious plants representing six varieties, only two of the four samples of the monoecious plants both representing one of the three analysed varieties were infected with this virus (Table 1). Thus, CanCV may not be as widespread in cultivated hemp plants in Europe as previously thought [17,18]. Supporting our findings, a recent survey in several locations in Colorado (USA) revealed CanCV only in one (indoor) plantation of unspecified hemp variety [10].

A novel partiti-like virus was identified in four samples (ALYU-308-309, ALYU-314-315) and completely or near-completely reconstructed from ALYU-315 and ALYU-309, having the highest and intermediate counts of viral sRNAs (Table 1; Dataset S1), respectively. The ALYU-309 isolate lacked a few nucleotides at 3′ and 5′ termini and differed from the ALYU-315 isolate at 35 SNP positions of its 1832 nt RNA genome (Dataset S2), thus sharing 98% nt identity. Blastn analysis showed that this virus shares 65.9 to 66.7% nt identity (at 71% query coverage) with Maize-associated partiti-like virus (MAPLV) isolates MW063111.1, MW063112.1 and MF372918.1 from maize (*Zea mays*) plants and RNA1 segment of *Partitiviridae* sp. isolate XZN141309 from bird faeces. A single ORF of the new virus (Figure 1a) encodes a putative RdRP of 563 amino acids with four substitutions distinguishing the two isolates and 63.4–66.8% identity (at 95% query coverage) to putative RdRPs encoded by the above-mentioned MAPLV isolates and *Partitiviridae* sp. isolate XZN141309 and 49–58% identity to RdRPs of other putative members of *Partitiviridae* of plant and non-plant origins. We, therefore, classified this new virus as a tentative member of *Partitiviridae* and named it Cannabis partiti-like virus (CanPLV). Our isolates of CanPLV lack a CP-encoding genomic segment, characteristic of the family *Partitiviridae* [18], as we were unable to find any sRNA contig presenting such a segment. This feature is shared by MAPLV isolates having only a single RdRP-encoding segment deposited in the GenBank. The presence of CanPLV does not correlate with any particular symptoms (Table 1), suggesting its cryptic nature. Notably, CanPLV was detected only in dioecious varieties (three of the six tested; Table 1), while CanCV appears to be restricted to monoecious varieties of hemp (Table 1; [17,18]).

**Figure 2:**
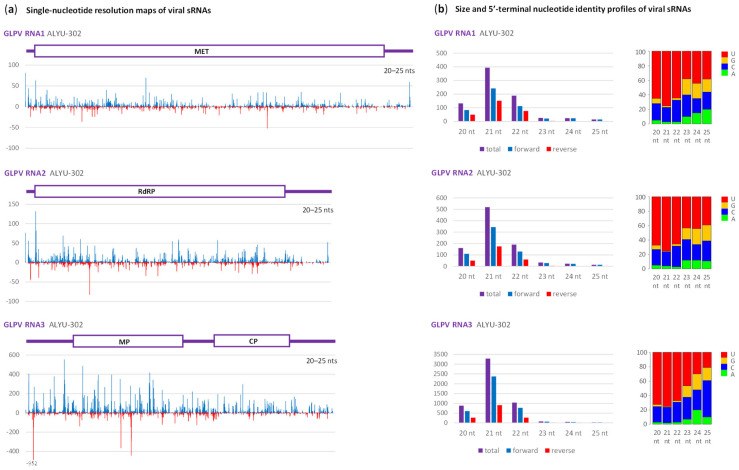
Illumina sequencing analysis of Grapevine line patter virus (GLPV)-derived small RNAs in the hemp plant ALYU-302. (**a**) Single-nucleotide resolution maps of 20–25 nt small (s)RNA reads mapped with zero mismatches on the reconstructed genome sequence of GLPV isolate ALYU-302. Histograms plot the numbers of viral reads at each nucleotide position of three RNA segments of the GLPV genome (RNA1, RNA2 and RNA3): blue bars above the axis represent positive-sense strand reads starting at each respective position, while red bars below the axis represent complementary strand reads ending at each respective position. The viral genome organization is shown schematically above the histograms, with the ORFs encoding methyltrasferase (MET) in RNA1, RNA-dependent RNA polymerase (RdRP) in RNA2, and movement (MP) and coat (CP) proteins in RNA3 boxed. (**b**) Size and 5′-terminal nucleotide identity profiles of viral sRNAs. Counts in reads per million (RPM) of sRNAs derived from positive-sense (forward) and negative-sense (reverse) strands or both strands of the viral genomic RNAs are plotted as bar graphs. Frequencies of 5′-terminal uridine (U), guanosine (G), cytosine (C) and adenosine (A) are plotted in percentage of total sRNAs for each of the six size classes (20, 21, 22, 23, 24 and 25 nt).

Three genomic RNA segments of the anulavirus GLPV, detected in ALYU-301 and ALYU-302 samples of the dioecious variety Perugina and fully reconstructed from ALYU-302 having a higher viral sRNA coverage (Table 1; Dataset S1), share the highest pairwise identity with RNA1 (98.7%), RNA2 (97.8%) and RNA3 (99.0%) of GLPV isolate “GLPV-Lar” (MW888424.1, MW888423.1 and MW888422.1, respectively). The latter isolate was recently detected and reconstructed using Illumina sequencing analysis of high-molecular-weight RNA from the indoor-cultivated hemp in Colorado (USA) in mixed infection with CanCV [10]. Previously, GLPV was identified as a causative agent of disease symptoms in grapevine (*Vitis vinifera*) plants in Hungary [20]. Our hemp isolate of GLPV from Italy shares a slightly lower identity (96.9, 97.8 and 98.4%) to the grapevine isolate Baco22A (MT319109.1, MT319110.1, MT319111.1) than to the hemp isolate from USA. Notably, the 3163 nt RNA1, 2496 nt RNA2 and 2531 nt RNA3 of our reconstructed GLPV genome appear to have complete 5′- and 3′-termini, similar to those of the grapevine isolate, while the USA isolate has incomplete termini (Dataset S2). Moreover, the evolutionarily conserved 3′-terminal tRNA-like structure shared by all the three segments of our GLPV isolate ends with the universal CCA required for aminoacylation, whereas the last nucleotide of the CCA motif is missing in all segments of the grapevine isolate and the isolate Rg24 from *Rehmannia glutinosa* (MZ395976.1, MZ395977.1, MZ395978.1) (Dataset S2). As the 5′- and 3′-termini of the grapevine isolate of GLPV were confirmed using 5′- and 3′-RACE [20], we assume that the last adenosine at the 3′-termini was likely omitted, being considered as part of artificial poly(A) tails added during 3′-RACE. The four conserved ORFs encoding methyltransferase (MET) in RNA1, RdRP in RNA2 and movement protein (MP) and CP in RNA3 are well preserved in our hemp isolate of GLPV (Figure 2a). Since the GLPV-positive sample ALYU-301 was a mixture of leaves from asymptomatic Perugina plants, while the second GLPV-positive sample ALYU-302 was composed of symptomatic leaves of a single Perugina plant with yellow/orange spots on the leaves (Figure S1), it is unclear if GLPV is a causal agent of these virus-like symptoms, even though viral sRNAs were ca. 6-times more abundant in the symptomatic leaves compared to non-symptomatic ones (Table 1; Dataset S1). Further studies using mechanical inoculations or agroinoculations with GLPV infections clones are needed to verify possible association between GLPV infection and symptom development in hemp plants.

The last component of Cannabis virome we identified is the mitovirus CasaMV1, already reported for *C. sativa* [21]. It was detected in 8 of the 19 samples representing 3 of the 6 dioecious and 1 of the 3 monoecious varieties; thus, it represented the most prevalent virome component. Its monopartite RNA genome was fully reconstructed from two dioecious varieties (ALYU-311-313 and ALYU-314), having the highest counts of viral sRNAs (Table 1; Dataset S1). The two reconstructed variants of the 2855 nt CasaMV1 genome differed by 10 SNPs within a single, RdRP-encoding ORF, which resulted in 3 amino acid substitutions, and were most closely related to the CasaMV1 isolates PurpleKush (NC_076527.1; 6 and 10 SNPs, respectively, and 1 and 4 amino acid substitutions, respectively) and MPC/MSU (3 and 7 SNPs, 2 and 5 amino acid substitutions, respectively) from Canada [21,22]. Members of the family *Mitoviridae* lack a coat protein and encode a single RdRP enabling their replication in mitochondria of fungi [23], invertebrates [24,25] and plants [21]. These viruses are generally considered to be cryptic. However, there is no information on their impact on plant hosts. The fact that we detected CasaMV1 in both symptomatic (*n* = 7) and non-symptomatic (*n* = 1) hemps plants would suggest its cryptic behaviour. Nonetheless, the highest levels of CasaMV1-derived sRNAs in the two dioecious varieties did coincide with the deformation of leaves (Table 1).

To further validate our Illumina sRNA sequencing results, we performed RT-PCR analysis of selected total RNA samples for the presence of all the four viruses detected and reconstructed from sRNA reads. The RT-PCR analysis using the primer pairs designed previously for CanCV and GLPV and in this study for CanPLV and CasaMV1 (Table S1) fully confirmed the Illumina sequencing results (Figure S4).

### 2.2. Interactions of Cannabis Virome Components with the Plant RNAi Machinery

Mapping of sRNAs on the reconstructed viral genomes with zero mismatches, followed by in-depth bioinformatics analysis of virus-derived sRNA profiles, revealed that both the classified (CanCV) and tentative (CanPLV) members of *Partitiviridae* spawn predominantly 21 nt and much less abundant 22 nt sRNAs from both strands of the entire virus genome (Figure 1; Dataset S1; Dataset S3).

These major size classes of viral sRNAs have predominantly 5′-terminal uridine (5′U) and cytosine (5′C) (Figure 1b). These findings suggest that *C. sativa* homologs of DCL4 and DCL2 process viral genomic dsRNA or dsRNA intermediates of viral replication into 21 nt (DCL4) and 22 nt (DCL2) siRNAs which are then sorted and stabilized by *C. sativa* homologs of AGO1 (5′U) and AGO5 (5′C), as established for other RNA viruses in the model dicot plants (reviewed in [15]). Similar profiles of viral sRNAs were reported for most families of plant RNA viruses (including *Partitiviridae*; [26]) in different species and families of land plants [15]. A bias to the genomic RNA strand observed for CanCV and less pronouncedly for CanPLV as well as local sRNA hotspots/peaks observed for both viruses (Figure 1a) can be explained by differential sRNA stability and/or biases of Illumina sRNA sequencing [15].

The anulavirus GLPV-derived sRNAs also represented both strands of entire genome, with a bias to the positive-sense strand and with the 21 nt class being predominant (Figure 2; Datasets S1; Dataset S3), albeit relatively less abundant than that of *Partitiviridae*.

Unlike *Partitiviridae*, the 20 nt class accumulated at much higher levels and was almost as abundant as the 22 nt class (Figure 2b). We assume that 20 nt sRNAs were derived from the longer size classes produced by *C. sativa* homologs of DCL4 and DCL2. All the three major size classes had predominantly 5′U (60–70%) and 5′C (20–30%), suggesting their association with *C. sativa* homologs of AGO1 and AGO5, respectively, like in the case of *Partitiviridae*. A previous study on GLPV in grapevine leaves showed that viral sRNAs are derived from both strands of the entire virus genome and are dominated by the 21 and, less abundant, 22 nt classes [20], indicating similar antiviral RNAi responses in *V. vinifera* (family Vitaceae) and *C. sativa* (family Cannabinaceae). Moreover, in both cases, the RNA3 encoding coat and movement proteins was a major source of viral siRNAs, likely reflecting higher abundance of this RNA compared to RNA1 and RNA2 encoding non-structural proteins (MET and RdRP, respectively).

**Figure 3:**
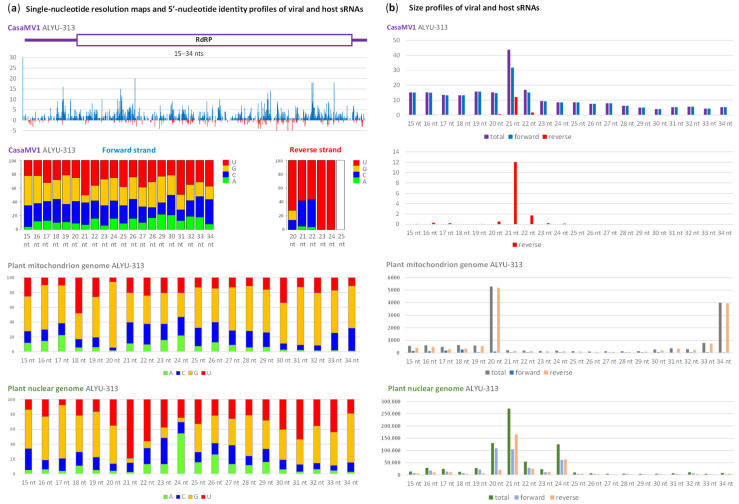
Illumina sequencing analysis of *Cannabis sativa* mitovirus 1 (CasaMV1) genome- and plant mitochondrion and nuclear genomes-derived small RNAs in the hemp plant ALYU-313. Here, 15–34 nt small (s)RNA reads were mapped with zero mismatches on the reconstructed genome sequence of GLPV isolate ALYU-313 and the reference sequences of *C. sativa* mitochondrion and nuclear genomes, sorted by size and 5′-terminal nucleotide identity and then counted. In panel (**a**), a histogram plots the combined numbers of 15–34 nt viral reads at each nucleotide position of the CasaMV1 genome: blue bars above the axis represent positive-sense strand reads starting at each respective position, while red bars below the axis represent complementary strand reads ending at each respective position. The viral genome organization is shown schematically above the histogram, with the ORF encoding RNA-dependent RNA polymerase (RdRP) boxed. Below the histogram, bar graphs show frequencies of 5′-terminal uridine (U), guanosine (G), cytosine (C) and adenosine (A) plotted in percentage of total sRNAs for each of the size classes. (**b**) Size profile of viral and host sRNAs. Bar graphs show counts in reads per million (RPM) of sRNAs derived from positive-sense (forward) and negative-sense (reverse) strands or both strands of the viral genome and from forward and reverse strands of the host plant genomes.

The mitovirus CasaMV1-derived sRNAs differed from those of other virome components in that they mostly represented the positive-sense strand and, on this strand, had a much wider size range (Figure 3; Dataset S3).

Nonetheless, on both strands of CasaMV1, the 21 nt size-class was the most abundant. On the negative-sense strand, the 21 nt and less abundant 22 nt sRNAs were predominant and were strongly enriched in 5′U and 5′A (Figure 3), similar to 21 and 22 nt siRNAs derived from other virome components. Interestingly, the positive-sense strand-derived sRNAs of these size classes were less pronouncedly enriched in 5′U and 5′C, while other size classes had less biased 5′-nucleotide frequencies, with 5′G-sRNAs (unknown to be preferred by any AGO) being more represented (Figure 3).

These findings, together with previous observation of a strong positive strand bias in fungal mitovirus-derived sRNAs [23], suggest that sRNAs of positive-sense polarity derived from CasMV1 genomic RNA are mostly produced by a DCL-independent (non-RNAi) degradation pathway or by a DCL1-like activity targeting secondary structures of the abundant positive-sense genomic RNA. On the other hand, a small fraction of those positive-sense sRNAs and the majority of negative-sense strand-derived sRNAs are likely generated by DCL4 and DCL2 from less abundant dsRNA intermediates of viral replication and then get associated with AGO1 and AGO5, similar to siRNAs derived from other virome components. The fact that CasMV1 is the only virus replicating in the mitochondria, while other virome components would replicate in the cytoplasm, raises questions about how dsRNA precursors of mitoviral siRNAs are exposed to the cytoplasmic DCL activities. Alternatively, both DCLs and AGOs would visit the mitochondria to target replicating CasaMV1. However, we found that mitochondrion genome-derived sRNAs have distinct size-class and 5′-nucleotide identity profiles, with the 22 nt class being predominant (having predominantly 5′G) and 21 and 22 classes being underrepresented with no substantial bias in 5′-terminal nucleotide frequencies (Figure 3). Notably, nuclear genome-derived sRNAs were found to have a profile typical for other land plants, with the dominant 21 and 24 nt classes enriched with 5′U and 5′A, respectively. These 21 and 24 nt classes of plant sRNAs are known to be populated mostly by the miRNAs generated by DCL1 and associated with AGO1 and the heterochromatic siRNAs generated by DCL3 and associated with AGO4 [11,12,15].

## 3. Materials and Methods

### 3.1. Plant Samples

Leaf samples were collected from dioecious and monoecious varieties of *Cannabis sativa* grown for inflorescence/cannabinoid and seed/fiber production (Table 1), respectively.

Seven samples were collected from vegetatively propagated female plants of the dioecious varieties Gerola (*n* = 4) and Perugina (*n* = 3) during a survey in province of Milano (Italy) in September 2021. All plants of the two varieties were sampled at the flowering stage and most of them exhibited virus-like symptoms, such as mosaic or spots on the leaves, leaf deformation, internode elongation or stunted growth; two mixed leaf samples from asymptomatic plants of each of the two varieties were also collected (Table 1). Eight additional samples from flowering female dioecious plants were collected in June 2022 in greenhouses in province of Milano. These plants exhibited virus-like symptoms such as variegation, deformation or wrinkling of the leaves (Table 1).

Four leaf samples from monoecious varieties of the industrial hemp were collected in April 2021 (*n* = 3) and June 2022 (*n* = 1). The plants were grown with photoperiod 16/8 h day/night, temperature 22/18 °C day/night and 70% humidity in a greenhouse of the faculty of Agronomy at the University of Milano. The samples were collected from one symptomless plant of the variety Felina 32 at around three weeks after seed germination and from three single plants with leaf deformations of the varieties USO 31, Futura 75 and Felina 32 at the beginning of the flowering stage around two months after seed germination.

In all cases, ~1 g of leaves from each plant were collected and kept at either −30 °C or −80 °C (Table 1). Examples of virus-like and other symptoms are shown in Figure S1.

### 3.2. RNA Extraction and Validation for Illumina Sequencing

Total RNA from the leaf samples was extracted using a CTAB-LiCl method as described previously [27]. The integrity of low-molecular-weight RNA was evaluated via electrophoresis on a 15% polyacrylamide-urea gel, followed by blot hybridization with a plant miR166-specific probe (Figure S2) as described previously [28]. An additional control of RNA integrity was performed prior to Illumina sequencing using a Fragment analyser for all the 19 samples, signs of degradation were detectable for 10 samples (those harvested in 2021 and stored at −30 °C; Table 1).

### 3.3. Illumina Sequencing and Bioinformatic Analysis of sRNAs

Small RNAs from all 19 samples (ALYU-297 to ALYU-315) were sequenced at Fasteris (www.fasteris.com; accessed on 1 November 2023) using Illumina Small RNA-Seq Gel-free protocol for cDNA library preparation and multiplexing cDNA libraries in one lane of NextSeq (1 × 75 nt run), which yielded 12′961′075 to 24′879′971 reads with Q30 ranging from 91.83 to 93.47. Demultiplexing and adapter trimming was performed, followed by sorting and counting reads by size. A histogram representation of the read length distribution showed that 21 and 24 nt classes dominate in many libraries. Samples showing signs of RNA degradation yielded a lower number of reads in the size range of 18 to 26 nts, with a more even and broad size distribution (Figure S3; Dataset S1).

To reconstruct virome components, we used a pipeline developed previously [14] and further improved [29]. Briefly, redundant or non-redundant reads ranging from 15 to 34 nts were assembled in contigs using Velvet 1.2.10 [30], followed by Oases 0.2.09 [31], with different k-mer lengths (13, 15, 17, 19 and 21) and a minimum contig length set to 50 nts. The resulting contigs were combined and mapped on *C. sativa* reference genomes available at the NCBI Genome resources (https://www.ncbi.nlm.nih.gov/genome/browse/#!/eukaryotes/11681/; accessed on 1 November 2023) using BWA-MEM 0.7.12 [32]. To discriminate between hemp varieties developed for cannabinoid or seed and fiber production, the contigs of the samples ALYU-297-to-303 and ALYU-308-to-315 were mapped on the merged reference genomes of cv. Abacus (assembly GCA_025232715.1) and cv. CBDRx-18 (GCA_900626175.2), while those of ALYU-304-to-307 were mapped on the reference genome of cv. Finola (assembly GCA_003417725.2). The unmapped non-host contigs were scaffolded using Seqman module of Lasergene DNASTAR 12.0.0 Core Suite (DNAStar, Madison, WI, USA). The resulting Seqman contigs were manually curated (if needed) and analysed with the NCBI blastn to search for viral contigs. If Seqman contigs represented incomplete viral genomes, a reference-based assembly of Oases contigs was performed using a reference sequence of the most-closely related viral isolate for which a complete genome was available. The reconstructed viral genome sequences were verified via BWA mapping of redundant sRNA reads ranging from 15 to 34 nts from the respective libraries. The resulting mapping data were then analysed using MISIS-2 [33] and corrected manually (if needed), followed by a new round of sRNA mapping to obtain the consensus sequence of each virus genome and identify SNPs (if any) present in the viral quasispecies population. Redundant sRNAs of each library were mapped on the reconstructed consensus genome sequences of all the virome components as well as the host genome (nuclear, chloroplast and mitochondrion), followed by sorting of the viral and host reads by size (15 to 35 nts, total 15–34 nt), polarity (forward, reverse, total) and 5′-nucleotide identity (5′A, 5′U, 5′G, 5′C) using an in-house script that generated the respective count tables (Dataset S1). Single-nucleotide resolution maps of viral sRNAs (Dataset S3) were generated using MISIS-2. The sRNA counts of each virome component in reads per million (RPM) are given in Table 1.

### 3.4. Reverse Transcription-Polymerase Chain Reaction (RT-PCR)

One microgram of total RNA was converted to cDNA using reverse transcriptase M-MLV (Promega, Madison, WI, USA) and random hexamer primers, following the manufacturer’s protocol. Then, 2 µL of cDNA were used to set up PCR with GoTaq (Promega, Madison, WI, USA), following the manufacturer’s instructions. A 2% agarose gel electrophoresis was performed to evaluate size and presence of the expected amplicons. We used previously designed primer pairs for detection of CanCV RdRP and CP genomic segments [18], LCV [7], BCTV [34], HLVd [35] and GLPV [20] as well as primer pairs designed here for detection of CasMV1 and CanPLV (see Table S1 for primer sequences and expected PCR amplicon sizes and Figure S4 for representative PCR results).

## Figures and Tables

**Table 1:** *Cannabis sativa* virome components identified through Illumina small RNA sequencing.

Sample/ Isolate	Cultivar/ Variety	Dio- or Mono-Ecious	CanCV (RPM *)	GLPV (RPM *)	CasaMV1 (RPM *)	CanPLV (RPM *)	Symptoms	Sampled in	Stored at	Signs of RNA Degradation
ALYU-297	Gerola	Dio			+ (58)		No	September 2021	−30 °C	Yes
ALYU-298	Gerola	Dio			+ (73)		Dwarfism and deformed leaves	September 2021	−30 °C	Yes
ALYU-299	Gerola	Dio					Dwarfism and deformed leaves	September 2021	−30 °C	Yes
ALYU-300	Gerola	Dio			+ (60)		Elongated internodes thin/narrow leaves	September 2021	−30 °C	Yes
ALYU-301	Perugina	Dio		+++ (1389)			No	September 2021	−30 °C	Yes
ALYU-302	Perugina	Dio		++++ (8734)			Yellow/orange spots on leaves	September 2021	−30 °C	Yes
ALYU-303	Perugina	Dio					Desiccated parts, dense new sprouts	September 2021	−30 °C	Yes
ALYU-304	Felina 32	Mono	+++ (2366)				No	June 2022	−80 °C	No
ALYU-305	USO 31	Mono					Leaves wrinkling and deformation	April 2021	−30 °C	Yes
ALYU-306	Felina 32	Mono	++ (588)		+ (39)		Leaves wrinkling and deformation	April 2021	−30 °C	Yes
ALYU-307	Futura 75	Mono					Leaves wrinkling and deformation	April 2021	−30 °C	Yes
ALYU-308	Feno A1	Dio				+ (22)	Variegation	June 2022	−80 °C	No
ALYU-309	Feno A2	Dio				++ (308)	Leaves wrinkling and deformation	June 2022	−80 °C	No
ALYU-310	Feno BB	Dio					Leaves wrinkling	June 2022	−80 °C	No
ALYU-311	K 1	Dio			++ (608)		Leaves deformation	June 2022	−80 °C	No
ALYU-312	K 3	Dio			++ (156)		Leaves deformation	June 2022	−80 °C	No
ALYU-313	K 4	Dio			++ (226)		Leaves deformation	June 2022	−80 °C	No
ALYU-314	Sweet 6	Dio			++ (190)	+ (26)	Leaves deformation	June 2022	−80 °C	No
ALYU-315	Queen sangria	Dio				++++ (6327)	Leaves wrinkling and deformation	June 2022	−80 °C	No

* RPM = reads per million; viral sRNAs were counted in reads per million of total (plant + viral) sRNA reads for each virus (CanCV, GLPV, CasaMV1, and CaPLV) identified in the respective samples and the resulting numbers are given in brackets. “+”, “++”, “+++” and “++++” indicate relative loads of viral siRNAs, based on the counts of sRNA reads.

## Data Availability

Complete nucleotide sequences of the reconstructed viral genomes are deposited in the NCBI GenBank under the following accession numbers: OR553229 (CanCV RNA1 isolate ALYU-304), OR553230 (CanCV RNA1 isolate ALYU-306), OR553231 (CanCV RNA2 isolate ALYU-304), OR553232 (CanCV RNA2 isolate ALYU-306), OR553233 (GLPV RNA1 isolate ALYU-302), OR553233 (GLPV RNA2 isolate ALYU-302), OR553235 (GLPV RNA3 isolate ALYU-302), OR553236 (CasaMV1 isolate ALYU-311-313), OR553237 (CasaMV1 isolate ALYU-314), OR553238 (CanPLV isolate ALYU-315) and OR553239 (CanPLV isolate ALYU-309). The raw Illumina-seq data were deposited to the NCBI SRA database under BioProject ID PRJNA1013875 (SAMN37312282: ALYU-297; SAMN37312283: ALYU-298; SAMN37312284: ALYU-299; SAMN37312285: ALYU-300; SAMN37312286: ALYU-301; SAMN37312287: ALYU-302; SAMN37312288: ALYU-303; SAMN37312289: ALYU-304; SAMN37312290: ALYU-305; SAMN37312291: ALYU-306; SAMN37312292: ALYU-307; SAMN37312293: ALYU-308; SAMN37312294: ALYU-309; SAMN37312295: ALYU-310; SAMN37312296: ALYU-311; SAMN37312297: ALYU-312; SAMN37312298: ALYU-313; SAMN37312299: ALYU-314; SAMN37312300: ALYU-315).

## Supplementary Materials

The following supporting information can be downloaded at: https://www.mdpi.com/article/10.3390/plants12233925/s1, Table S1: PCR primers used for detection of Cannabis virome components; Figure S1: Examples of virus-like and other symptoms of hemp plants; Figure S2: Small RNA blot hybridization analysis of total RNA from hemp plants; Figure S3: Size profiles of Illumina small RNA reads for 19 leaf samples (ALYU-297-315) of hemp plants; Figure S4: RT-PCR analysis of total RNA extracted from Cannabis leaf samples. Dataset S1: Counts of Illumina small RNA-seq reads from hemp plants; Dataset S2: Reconstructed genomes of Cannabis virome components and their comparison with other isolates of known viruses or related viruses; Dataset S3: Single-nucleotide resolution maps of viral small (s)RNAs.
