# Supplementary material for: Cannabis Virome Reconstruction and Antiviral RNAi Characterization through Small RNA Sequencing
Source: Plants (Basel). 2023 Nov 21;12(23):3925. doi: 10.3390/plants12233925 (PMC10707731; doi:10.3390/plants12233925)
Supplement: Supplementary file 1 [file plants-12-03925-s001.zip › Dataset S2.pdf]

Dataset S2. Reconstructed genomes of Cannabis virome components and their comparison with other isolates of known viruses or related viruses. (**A**) Cannabis cryptic virus (CanCV). (**B**) Cannabis partiti-like virus (CanPLV). (**C**) Grapevine line pattern virus (GLPV). (**D**) Canabis sativa mitovirus 1 (CasaMV1).

(A) Cannabis cryptic virus (CanCV)

```
>CanCV_RNA1_RdRp_304
AGATTTTTTATAAGCGCCCCCCGCCTTATTTAACTAAGAATTAACATTCGTCTAAGTTATCAAGCCTTCACAAAGCCTCTGCTCTAATTATTCACCACAACGTCTCACCATGCCTTACAACGCTGTCCGCAATTATCTTGCTGAACGTAT
GATCCGCACAAAAAGAGAATGGCAACTTTACCAATCCTCTAACCGTGATCCTGAATCGACTCTTGAACATTATCAAGATTCTGACTACTTACGTTATTACCAGAATGCCCGTTTTAACCTTAACCAAGAAGAACGATTCCGCGCCTTAAA
CAAAGAATACTCTACACTAGTTGAAGCATTTAGAACTGACAACCTACGAAAGCACCAACCTTATGAGCTTCACCAGCCCATTCCTCCTGATGCTGCCCCTATTCCTGACAAACGTCAGCCTGCCCCTGGAATTAAATTAGTCCCACTCAT
GTACCACTATGGACATGTTGTTCATGATACCCCTTCTACTACTGATGATCATACATCAAATGATGATTCTCAATCAGCACCATCCAGACTGTTAAAAACTACTGAATTTGGTTACTCCATTGACAAACAAATCTATGATCTCGTTTGCCG
TAGATATCCTACTTATTTATCAGTTATTAACATGTACTGTCGACCCCTCGGTACTGTAGATGCTACATTCTCTGACTTTAACAAAGAACAGATACCATCTAATCCGATTGATCCTGATAGGAAAGAACATGTTCTGAAACACATTTTTAA
GTTTCTGGACGCCACCCCTTACTTACCTGTCCACTTTGTTGACACTCAATTCTGCAAGACTCCTCTTGTTACTGGAACTGGCTACCATAACCGCTATTCTTTCAAACAGAAAGCACATGCAAAGTATTCTCACCCTGAAGAATATGCCAC
ATACCCTTCCTCTAAAGGCTACTTCTACAATGCTACTTATGAGAATGCCCGCACTTTGGTTCACTACATCAAACAAAATGGTCTACCATTTGAATTTGATTTTAATCTCTCTGAAGAATCCCTGACCGACGAACAAATTGATTTATTCGT
CAACAGATCCAATGCCTTTTTCAATGATTACCCGACGTTACTATTCACTCGTAATCACATTTCGAAGCGTACTGGACCACTAAAAGTCCGCCCTGTTTACGCTGTTGATGATATTTTTATCATTATTGAACTGATGTTGACCTTCCCTCT
CACTGTTCAAGCTCGAAAACCCTCATGCTGTATTATGTATGGCTTCGAAACCATTCGTGGTTCAAACCGATACATTGACAGACTTGCTCGTTCGTATTCGACCTTCTTTTCATTAGACTGGTCAAGTTATGATCAACGACTGCCCCGTGT
AATTACTGACATTTATTACACTGACTTCCTTCGAAGCCTGATCGTTATTAACCATGGATACCAACCCACGTATGAATACTCCTCTTACCCTGACCTTGATGAACATAAGCTCTATGATAGAATGAATAATCTTCTTTTCTTTCTTCATAC
ATGGTATAACAATATGACCTTCCTTCTCCCGGATGGTTTTGCATACCGCAGATCTTACTGTGGAGTCCCTTCTGGCCTCTACAATACTCAATACCTTGATTCTTTTGGCAATCTATTTTTAATAATTGATGCCATGATTGAATTCGGTTT
CACTGACTCTGAAATCGATGGATTTGTCCTCCTAATTTTAGGTGATGACAACACTGGTATGACACAGATGCACATTCATCGCATCTCCCAATTCATCAACTTTCTTGAAAAATATGCTTTAGAACGCTACAACATGGTCCTCTCTGCGAC
CAAATCTGTTCTAACAACTCTGCGCTCAAAAATTGAGACCCTTGGTTACCAATGTAACTATGGATCCCCTAAACGCGACATTGATAAACTCGTTGCCCAACTCTGTTTCCCTGAAAATGGACTCAAGCCACACACCATGTCTGCTAGAGC
TGTCGGAATCGCATATGCCGCTGCCGGCCAAGATTACGTATTTCACTCCTTTTGCCAGGATGTATATAATATGTTTAGGTCTTATTATAAACCCGATGCTCGTGCAAATCTATTCTTCCAACGTCAAGTACTCCAAAATCTTGAAGATGG
AATTCCTGATTTAGCCACACCGGTTGTGCCGCCATTCCCTTCATTATTTGAAGTCCGCGAGATGTACTCTGAATATAAAGGACCTCTCTCTTTTGAACCTAAATGGAACAAAGCTCACTTCATAAATGACCCGAATGACATCCCTCCTTT
CTCCAAAACTATGCGAGACTATGAAAAGGAACATAATCTTCCTGTTCGAGTTTCACCTACTTTTGAAACGGTCGTGCCTAGCACAAAAAATTTGCCGTAATTTTTTATCTTCGTTTTAAATTTCGTTGATTTTGCAAAT

>CanCV_RNA1_RdRp_306
AGATTTTTTATAAGCGCCCCCCGCCTTATTTAACTAAGAATTAACATTCGTCTAAGTTATCAAGCCTTCACAAAGCCTCTGCTCTAATTATTCACCACAACGTCTCACCATGCCTTACAACGCTGTCCGCAATTATCTTGCTGAACGTAT
GATCCGCACAAAAAGAGAATGGCAACTTTACCAATCCTCTAACCGTGATCCTGAATCGACTCTTGAACATTATCAAGATTCTGACTACTTACGTTATTACCAGAATGCCCGTTTTAACCTTAACCAAGAAGAACGATTCCGCGCCTTAAA
CAAAGAATACTCTACACTAGTTGAAGCATTTAGAACTGACAACCTACGAAAGCACCAACCTTATGAGCTTCACCAGCCCATTCCTCCTGATGCTGCCCCTATTCCTGACAAACGTCAGCCTGCCCCTGGAATTAAATTAGTCCCACTCAT
GTACCACTATGGACATGTTGTTCATGATACCCCTTCTACTACTGATGATCATACATCAAATGATGATTCTCAATCAGCACCATCCAGACTGTTAAAAACTACTGAATTTGGTTACTCCATTGACAAACAAATCTATGATCTCGTTTGCCG
TAGATATCCTACTTATTTATCAGTTATTAACTTGTACTGTCGACCCCTCGGTACTGTAGATGCTACATTCTCTGACTTTAACAAAGAACAGATACCATCTAATCCGATTGATCCTGATAGGAAAGAACATGTTCTGAAACACATTTTTAA
GTTTCTGGACGCCACCCCTTACTTACCTGTCCACTTTGTTGACACTCAATTCTGCAAGACTCCTCTTGTTACTGGAACTGGCTACCATAACCGCTATTCTTTCAAACAGAAAGCACATGCAAAGTATTCTCACCCTGAAGAATATGCCAC
ATACCCTTCCTCTAAAGGCTACTTCTACAATGCTACTTATGAGAATGCCCGCACTTTGGTTCACTACATCAAACAAAATGGTCTACCATTTGAATTTGATTTTAATCTCTCTGAAGAATCCCTGACCGACGAACAAATTGATTTATTCGT
CAACAGATCCAATGCCTTTTTCAATGATTACCCGACGTTACTATTCACTCGTAATCACATTTCGAAGCGTACTGGACCACTAAAAGTCCGCCCTGTTTACGCTGTTGATGATATTTTTATCATTATTGAACTGATGTTGACCTTCCCTCT
CACTGTTCAAGCTCGAAAACCCTCATGCTGTATTATGTATGGCTTCGAAACCATTCGTGGTTCAAACCGATACATTGACAGACTTGCCCGTTCGTATTCGACCTTCTTTTCATTAGACTGGTCAAGTTATGATCAACGACTGCCCCGTGT
AATTACTGACATTTATTACACTGACTTCCTTCGAAGCCTGATCGTTATTAACCATGGATACCAACCCACGTATGAATACTCCTCTTACCCTGACCTTGATGAACATAAGCTCTATGATAGAATGAATAATCTTCTTTTCTTTCTTCATAC
ATGGTATAACAATATGACCTTCCTTCTCCCGGATGGTTTTGCATACCGCAGATCTTACTGTGGAGTCCCTTCTGGCCTCTACAATACTCAATACCTTGATTCTTTTGGCAATCTATTTTTAATAATTGATGCCATGATTGAATTCGGTTT
CACTGACTCTGAAATCGATGGATTTGTCCTCCTAATTTTAGGTGATGACAACACTGGTATGACACAGATGCACATTCATCGCATCTCCCAATTCATCAACTTTCTTGAAAAATATGCTTTAGAACGCTACAACATGGTCCTCTCTGCGAC
CAAATCTGTTCTAACAACTCTGCGCTCAAAAATTGAGACCCTCGGTTACCAATGTAACTATGGATCCCCTAAACGCGACATTGATAAACTTGTTGCCCAACTCTGTTTCCCTGAAAATGGACTCAAGCCACACACCATGTCTGCTAGAGC
TGTCGGAATCGCATATGCCGCTGCCGGCCAAGATTACGTATTTCACTCCTTTTGCCAGGATGTATATAATATGTTTAGGTCTTATTATAAACCCGATGCTCGTGCAAATCTATTCTTCCAACGTCAAGTACTCCAAAATCTTGAAGATGG
AATTCCTGATTTAGCCACACCGGTTGTGCCGCCATTCCCTTCATTATTCGAAGTCCGCGAGATGTACTCTGAATACAAAGGACCTCTCTCTTTTGAACCTAAATGGAACAAAGCTCACTTCATAAATGACCCGAATGACATCCCTCCTTT
CTCCAAAACTATGCGAGACTATGAAAAGGAACATAATCTTCCTGTTCGAGTTTCACCTACTTTTGAAACGGTCGTGCCTAGCACAAAAAATTTGCCGTAATTTTTTATCTTCGTTTTAAATTTCGTTGATTTTGCAAAT
```

```
>CanCV_RNA2_CP_304
AGATTTTTCTAAAGCGCCCcgCCTTTAGTTATTAAGAATTTACATTCGTCTATATTATCAAGTCCTCGAAAAACCTCTGCTCTAATTTTAAGATGTCTTCCTCAACCATCACTTCCCGTCTCGATGCCCTCAAAAGCAAGAACGTGATAA
TTGAATTCAAAGAAGCGAAAACTCCTGTCCCCTCTGCTGACATTTTGCAACTCTTTGCTGACAACACCGCCGCCCGCTCTGACTCATCCAATGAATGGATTATTGATGTTTTCCCAAACTTCACTCCCATCCTGATGTACATTATGCTCA
ATGCGATTCGCCACGCGAACGCCACTGCTCAACGTGAACATTCCAAGTCATCTGTTGCCACCATTTGCATGTATCATCTGTCCATTGTCTATGGATTTTTCCTCATCAATGACCTCAATGTCCGTCCTGCTCCGTCTGCTCATGCCCGTA
TCTGGGCCGAATCCAACTGGAGATCTGAATTTGTGAAATTTCTGTCCACCTTGCCTGTACCTGAGTTCCTTATTCCCATCCTATCCCAGTTTCACGCCTTTGAAACCGCAAGAACAAAGAATGTTTTCTTTATTCCCTCTGCCGCCGGCT
ATGACCACGACCAATTCTTCGGACGTGTTTATCCTTTGAATATGTTCGCCGCAATTCATGACTGTACTGCCACACTGCCCGGAAACTCCTCCAAAATCGATGTCCTTAGAGACCTCTACTCCAGAGTACTCTACTCAATTACTGACCCAG
CCTACACCTGCGTCATTCCCGATCTTATTGGAATCACCCTTAGCGCTGCTGACATTACCCAAGCTCAATACATGAATTCAAAACTTTACCAAGTTTTCTCTTCTGTTATTAATCCTGTCCTGTTCCGCGACTTTCAGCGCCGATCCTCCC
TCGCCGCTCTATCATTCAAAGCTCCAACGTTTGCCTCTGCTAACGTTAACGCTTATGATCTGATGTTTTCAGCCACTTCCGCTAATCTGCGTGAATTAAAGGTTGTCCTACAATCAGTCCATGCCGTTATCTCTGATGATGTATCATGTA
AGACCAGCCTTGGTCAATTTATTGCTGATGCTACCACCTCAACAATCATTAAACATGGATACTCCACATACGCTCTTCCCACCTGGTCCCACAGTGAAGTCACCAACAAAGCTCAAACCTTTCGTGCCATTACTGCCCAACAACTAATCT
CTGAAGATGCCCGTGCCCAAGACTTCTGTTTCCTTCAACGCCCTACAGCTGAAATTCCTCACACCCACAACCTCGAAGACGTACGTTACGTACGCACCGATGCCCCCACTGAACCAGTCGCTCTGCCCCAGAATCACAGTATCATCCGTA
ACTTCCCTCTCTGCCTCAGAAACAATACGAATGCCGCCGTCTTTCCCCGCGTAGACAACAACACACTGATCAATTTTAGTGAAGAAATCCACACTGTTCCAAGCGTCTTAGTGCTCGACACCGATGGTGATCAAACCATCACTGCCCACT
TGCCACTACTTGCCGGAAAGATCATTGAATCTTTTGAACTCGATGGAACCACCATCGAAATGCCCAATGCCATGAAATCACTCGGCATGCAAAACTGCATGTTCGCTGACTCTGCCATTCCGTACAAGTATGTTCGCCCTGGCTCCGCTT
ACCATCCCGTTAATGCTGGAACCATACTGCCGCCCCGTTCTCGAGCTACTCCCAATTCCACTCCTCGCCTGCCTGCCTCGTCGTTCCTATACAATCGACTGCGTGTTAATCTCCCCCGATTCAACCTCGCTATCAATGAAGAACGCATTG
GCAACATTCTCCCAGGAATGACTCTACTTGATGGAGTCACTGTCCTCCGGTATGTTCAGTCCTTTCTAGGATTCAACACCGTCAGCCGTGCTGACAATGCCGACAATGGAGACAATGTCCCGGCGATGCCCCGGAACCTGCTTCTGGTCT
GGTCCCCGTACACGTACACTCCGTATGAATCTGATGACCTGCCTGAACCAGCACTTGGCGAATCAAGGCACTACTACTTGACAAACCTCCGTACCATCTTCGGAACGGACTACAATCTTGTTCAAGCCAAGCATCCCTATGAATCGCTCC
CCGTCGTTTAAGCAATCTCTCTCATCCTCTTAATGTTTAAGTTTTATGATGTTTGATTGGCCTCCTTTTCCGACAAACACGTGTATGCTACGTGTTTTGTTTTTTTTCGTTTTATTTCCTGAACTTTTTATTTTCAGTCTTTTTTAACGT
TATTTTCTACTTTAAG

>CanCV_RNA2_CP_306
AGATTTTTCTAAAGCGCCCcgCCTTTAGTTATTAAGAATTTACATTCGTCTATATTATCAAGTCCTCGAAAAACCTCTGCTCTAATTTTAAGATGTCTTCCTCAACCATCACTTCCCGTCTCGATGCCCTCAAAAGCAAGAACGTGATAA
TTGAATTCAAAGAAGCGAAAACTCCTGTCCCCTCTGCTGACATTTTGCAACTCTTTGCTGACAACACCGCCGCCCGCTCTGACTCATCCAATGAATGGATTATATATGTTTTCCCAAACTTCACTCCCATCCTGATGTACATTATGCTCA
ATGCGATTCGCCACGCGAACGCCACTGCTCAACGTGAACATTCCAAGTCATCTGTTGCCACCATTTGCATGTATCATCTGTCCATTGTCTATGGATTTTTCCTCATCAATGACCTCAATGTCCGTCCTGCTCCGTCTGCTCATGCCCGTA
TCTGGGCCGAATCCAACTGGAGATCTGAATTTGTGAAATTTCTGTCCACCTTGCCTGTACCTGAGTTCCTTATTCCCATCCTATCCCAGTTTCACGCCTTTGAAACCGCAAGAACAAAGAATGTTTTCTTTATTCCCTCTGCCGCCGGCT
ATGACCACGACCAATTCTTCGGACGTGTTTATCCTTTGAATATGTTCGCCGCAATTCATGACTGTACTGCCACACTGCCCGGAAACTCCTCCAAAATCGATGTCCTTAGAGACCTCTACTCCAGAGTACTCTACTCAATTACTGACCCAG
CCTACACCTGCGTCATTCCCGATCTTATTGGAATCACCCTTAGCGCTGCTGACATTACCCAAGCTCAATACATGAATTCAAAACTTTACCAAGTTTTCTCTTCTGTTATTAATCCTGTCCTGTTCCGCGACTTTCAGCGCCGATCCTCCC
TCGCCGCTCTATCATTCAAAGCTCCAACGTTTGCCTCTGCTAACGTTAACGCTTATGATCTGATGTTTTCAGCCACTTCCGCTAATCTGCGTGAATTAAAGGTTGTCCTACAATCAGTCCATGCCGTTATCTCTGATGATGTATCATGTA
AGACCAGCCTTGGTCAATTTATTGCTGATGCTACCACCTCAACAATCATTAAACATGGATACTCCACATATGCTCTTCCCACCTGGTCCCACAGTGAAGTCACCAACAAAGCTCAAACCTTTCGTGCCATTACTGCCCAACAACTAATCT
CTGAAGATGCCCGTGCCCAAGACTTCTGTTTCCTTCAACGCCCTACAGCTGAAATTCCTCACACCCACAACCTCGAAGACGTACGTTACGTACGCACCGATGCCCCCACTGAACCAGTCGCTCTGCCCCAGAATCACAGTATCATCCGTA
ACTTCCCTCTCTGCCTCAGAAACAATACGAATGCCGCCGTCTTTCCCCGCGTAGACAACAACACACTGATCAATTTTAGTGAAGAAATCCACACTGTTCCAAGCGTCTTAGTGCTCGACACCGATGGTGATCAAACCATCACTGCCCACC
TGCCACTACTTGCCGGAAAGATCATTGAATCTTTTGAACTCGATGGAACCACTATCGAAATGCCCAATGCCATGAAATCACTCGGCATGCAAAACTGCATGTTCGCTGACTCTGCCATTCCGTACAAGTATGTTCGCCCTGGCTCCGCTT
ACCATCCCGTTAATGCTGGAACCATACTGCCGCCCCGTTCTCGAGCTACTCCCAATTCCACTCCTCGCCTGCCTGCCTCGTCGTTCCTATACAATCGACTGCGTGTTAATCTCCCCCGATTCAACCTCGCTATCAATGAAGAACGCATTG
GCAACATTCTCCCAGGAATGACTCTACTTGATGGAGTCACCGTCCTCCGGTATGTTCAGTCCTTTCTAGGATTCAACACCGTCAGCCGTGCTGACAATGCCGATAATGGAGACAATGTCCCGGCGATGCCCCGGAACCTGCTTCTGGTCT
GGTCCCCGTACACGTACACTCCGTATGAATCTGATGACCTGCCTGAACCAGCACTTGGCGAATCAAGGCACTACTACTTGACAAACCTCCGTACCATCTTCGGAACGGACTACAATCTTGTTCAAGCCAAGCATCCTTATGAATCGCTCC
CCGTCGTTTAAGCAATCTCTCTCATCCTCTTAATGTTTAAGTTTTATGATGTTTGATTGGCCTCCTTTTCCGACAAACACGTGTATGCTACGTGTTTTGTTTTTTTTCGTTTTATTTCCTGAACTTTTTATTTTCAGTCTTTTTTAACGT
TATTTTCTACTTTAAG
```

>NC_031134.1 Cannabis cryptic virus isolate hemp09 RNA-dependent RNA polymerase gene, complete cds

>MT893743.1 Cannabis cryptic virus isolate CSU RNA-dependent RNA polymerase gene, partial cds

>JN196536.1 Cannabis cryptic virus isolate Fedora17 putative RNA polymerase gene, complete cds

>NC_031130.1 Cannabis cryptic virus isolate hemp09 coat protein gene, complete cds

>JN196537.1 Cannabis cryptic virus isolate Fedora17 putative coat protein gene, complete cds

```
CLUSTAL multiple sequence alignment by MUSCLE (3.8)

JN196536.1              -GATTTTTTATAAGCGCCCCCCGCCTTATTTAACTAAGAATTAACATTCGTCTAAGTTAT
NC_031134.1             AGATTTTTTATAAGCGCCCCGCGCCTTATTTAACTAAGAATTAACATTCGTCTAAGTTAT
CanCV_RNA1_RdRp_304     AGATTTTTTATAAGCGCCCCCCGCCTTATTTAACTAAGAATTAACATTCGTCTAAGTTAT
CanCV_RNA1_RdRp_306     AGATTTTTTATAAGCGCCCCCCGCCTTATTTAACTAAGAATTAACATTCGTCTAAGTTAT
MT893743.1              -------------------------TTATTTAACTAAGAATTAACATTCGTCTAAGTTAT
                                                 ***********************************

JN196536.1              CAAGCCTTCACAAAGCCTCTGCTCTAATTATTCACCACAACGTCTCACCATGCCTTACAA
NC_031134.1             CAAGCCTTCACAAAGCCTCTGCTCTAATTATTCACCACAACGTCTCACCATGCCTTACAA
CanCV_RNA1_RdRp_304     CAAGCCTTCACAAAGCCTCTGCTCTAATTATTCACCACAACGTCTCACCATGCCTTACAA
CanCV_RNA1_RdRp_306     CAAGCCTTCACAAAGCCTCTGCTCTAATTATTCACCACAACGTCTCACCATGCCTTACAA
MT893743.1              CAAGCCTTCACAAAGCCTCTGCTCTAATTATTCACCACAACGTCTCACCATGCCTTACAA
                        ************************************************************

JN196536.1              CGCTGTCCGCAATTATCTTGCTGAACGTATGATCCGCACAAAAAGAGAATGGCAACTTTA
NC_031134.1             CGCTGTCCGCAATTATCTTGCTGAACGTATGATCCGCACAAAAAGAGAATGGCAACTTTA
CanCV_RNA1_RdRp_304     CGCTGTCCGCAATTATCTTGCTGAACGTATGATCCGCACAAAAAGAGAATGGCAACTTTA
CanCV_RNA1_RdRp_306     CGCTGTCCGCAATTATCTTGCTGAACGTATGATCCGCACAAAAAGAGAATGGCAACTTTA
MT893743.1              CGCTGTCCGCAATTATCTTGCTGAACGTATGATCCGCACAAAAAGAGAATGGCAACTTTA
                        ************************************************************

JN196536.1              CCAATCCTCTAACCGTGATCCTGAATCGACTCTTGAACATTATCAAGATTCTGACTACTT
NC_031134.1             CCAATCCTCTAACCGTGATCCTGAATCGACTCTTGAACATTATCAAGATTCTGACTACTT
CanCV_RNA1_RdRp_304     CCAATCCTCTAACCGTGATCCTGAATCGACTCTTGAACATTATCAAGATTCTGACTACTT
CanCV_RNA1_RdRp_306     CCAATCCTCTAACCGTGATCCTGAATCGACTCTTGAACATTATCAAGATTCTGACTACTT
MT893743.1              CCAATCCTCTAACCGTGATCCTGAATCGACTCTTGAACATTATCAAGATTCTGACTACTT
                        ************************************************************
```

```
JN196536.1                ACGTTATTACCAGAATGCCCGTTTTAACCTTAACCAAGAAGAACGATTCCGCGCCTTAAA
NC_031134.1               ACGTTATTACCAGAATGCCCGTTTTAACCTTAACCAAGAAGAACGATTCCGCGCCTTAAA
CanCV_RNA1_RdRp_304       ACGTTATTACCAGAATGCCCGTTTTAACCTTAACCAAGAAGAACGATTCCGCGCCTTAAA
CanCV_RNA1_RdRp_306       ACGTTATTACCAGAATGCCCGTTTTAACCTTAACCAAGAAGAACGATTCCGCGCCTTAAA
MT893743.1                ACGTTATTACCAGAATGCCCGTTTTAACCTTAACCAAGAAGAACGATTCCGCGCCTTAAA
                          ************************************************************

JN196536.1                CAAAGAATACTCTACACTAGTTGAAGCATTAGAACTGACAACCTACGAAAGCACCAACC
NC_031134.1               CAAAGAATACTCTACACTAGTTGAAGCATTAGAACTGACAACCTACGAAAGCACCAACC
CanCV_RNA1_RdRp_304       CAAAGAATACTCTACACTAGTTGAAGCATTAGAACTGACAACCTACGAAAGCACCAACC
CanCV_RNA1_RdRp_306       CAAAGAATACTCTACACTAGTTGAAGCATTAGAACTGACAACCTACGAAAGCACCAACC
MT893743.1                CAAAGAATACTCTACACTAGTTGAAGCATTAGAACTGACAACCTACGAAAGCACCAACC
                          ************************************************************

JN196536.1                TTATGAGCTTCACCAGCCCATTCCTCCTGATGCTGCCCCTATTCCTGACAAACGTCAGCC
NC_031134.1               TTATGAGCTTCACCAGCCCATTCCTCCTGATGCTGCCCCTATTCCTGACAAACGTCAGCC
CanCV_RNA1_RdRp_304       TTATGAGCTTCACCAGCCCATTCCTCCTGATGCTGCCCCTATTCCTGACAAACGTCAGCC
CanCV_RNA1_RdRp_306       TTATGAGCTTCACCAGCCCATTCCTCCTGATGCTGCCCCTATTCCTGACAAACGTCAGCC
MT893743.1                TTATGAGCTTCACCAGCCCATTCCTCCTGATGCTGCCCCTATTCCTGACAAACGTCAGCC
                          ************************************************************

JN196536.1                TGCCCCTGGAATTAAATTAGTCCCACTCATGTACCACTATGGACATGTTGTTCATGATAC
NC_031134.1               TGCCCCTGGAATTAAATTAGTCCCACTCATGTACCACTATGGACATGTTGTTCATGATAC
CanCV_RNA1_RdRp_304       TGCCCCTGGAATTAAATTAGTCCCACTCATGTACCACTATGGACATGTTGTTCATGATAC
CanCV_RNA1_RdRp_306       TGCCCCTGGAATTAAATTAGTCCCACTCATGTACCACTATGGACATGTTGTTCATGATAC
MT893743.1                TGCCCCTGGAATTAAATTAGTCCCACTCATGTACCACTATGGACATGTTGTTCATGATAC
                          ************************************************************

JN196536.1                CCCTTCTACTACTGATGATCATACATCAAATGATGATTCTCAATCAGCACCATCCAGACT
NC_031134.1               CCCTTCTACTACTGATGATCATACATCAAATGATGATTCTCAATCAGCACCATCCAGACT
CanCV_RNA1_RdRp_304       CCCTTCTACTACTGATGATCATACATCAAATGATGATTCTCAATCAGCACCATCCAGACT
CanCV_RNA1_RdRp_306       CCCTTCTACTACTGATGATCATACATCAAATGATGATTCTCAATCAGCACCATCCAGACT
MT893743.1                CCCTTCTACTACTGATGATCATACATCAAATGATGATTCTCAATCAGCACCATCCAGACT
                          ************************************************************

JN196536.1                GTTAAAAACTACTGAATTTGGTTACTCCATTGACAAACAAATCTATGATCTCGTTTGCCG
NC_031134.1               GTTAAAAACTACTGAATTTGGTTACTCCATTGACAAACAAATCTATGATCTCGTTTGCCG
CanCV_RNA1_RdRp_304       GTTAAAAACTACTGAATTTGGTTACTCCATTGACAAACAAATCTATGATCTCGTTTGCCG
CanCV_RNA1_RdRp_306       GTTAAAAACTACTGAATTTGGTTACTCCATTGACAAACAAATCTATGATCTCGTTTGCCG
MT893743.1                GTTAAAAACTACTGAATTTGGTTACTCCATTGACAAACAAATCTATGATCTCGTTTGCCG
                          ************************************************************

JN196536.1                TAGATATCCTACTTATTTATCAGTTATTAACTTGTACTGTCGACCCCTCGGTACTGTAGA
NC_031134.1               TAGATATCCTACTTATTTATCAGTTATTAACTTGTACTGTCGACCCCTCGGTACTGTAGA
CanCV_RNA1_RdRp_304       TAGATATCCTACTTATTTATCAGTTATTAACATGTACTGTCGACCCCTCGGTACTGTAGA
CanCV_RNA1_RdRp_306       TAGATATCCTACTTATTTATCAGTTATTAACTTGTACTGTCGACCCCTCGGTACTGTAGA
MT893743.1                TAGATATCCTACTTATTTATCAGTTATTAACTTGTACTGTCGACCCCTCGGTACTGTAGA
                          ******************************* ****************************

JN196536.1                TGCTACATTCTCTGACTTTAACAAAGAACAGATACCATCTAATCCGATTGATCCTGATAG
NC_031134.1               TGCTACATTCTCTGACTTTAACAAAGAACAGATACCATCTAATCCGATTGATCCTGATAG
CanCV_RNA1_RdRp_304       TGCTACATTCTCTGACTTTAACAAAGAACAGATACCATCTAATCCGATTGATCCTGATAG
CanCV_RNA1_RdRp_306       TGCTACATTCTCTGACTTTAACAAAGAACAGATACCATCTAATCCGATTGATCCTGATAG
MT893743.1                TGCTACATTCTCTGACTTTAACAAAGAACAGATACCATCTAATCCGATTGATCCTGATAG
                          ************************************************************

JN196536.1                GAAAGAACATGTTCTGAAACACATTTTTAAGTTTCTGGACGCCACCCCTTACTTACCTGT
NC_031134.1               GAAAGAACATGTTCTGAAACACATTTTTAAGTTTCTGGACGCCACCCCTTACTTACCTGT
CanCV_RNA1_RdRp_304       GAAAGAACATGTTCTGAAACACATTTTTAAGTTTCTGGACGCCACCCCTTACTTACCTGT
CanCV_RNA1_RdRp_306       GAAAGAACATGTTCTGAAACACATTTTTAAGTTTCTGGACGCCACCCCTTACTTACCTGT
MT893743.1                GAAAGAACATGTTCTGAAACACATTTTTAAGTTTCTGGACGCCACCCCTTACTTACCTGT
                          ************************************************************

JN196536.1                CCACTTTGTTGACACTCAATTCTGCAAGACTCCTCTTGTTACTGGAACTGGCTACCATAA
NC_031134.1               CCACTTTGTTGACACTCAATTCTGCAAGACTCCTCTTGTTACTGGAACTGGCTACCATAA
CanCV_RNA1_RdRp_304       CCACTTTGTTGACACTCAATTCTGCAAGACTCCTCTTGTTACTGGAACTGGCTACCATAA
CanCV_RNA1_RdRp_306       CCACTTTGTTGACACTCAATTCTGCAAGACTCCTCTTGTTACTGGAACTGGCTACCATAA
MT893743.1                CCACTTTGTTGACACTCAATTCTGCAAGACTCCTCTTGTTACTGGAACTGGCTACCATAA
                          ************************************************************

JN196536.1                CCGCTATTCTTTCAAACAGAAAGCACATGCAAAGTATTCTCACCCTGAAGAATATGCCAC
NC_031134.1               CCGCTATTCTTTCAAACAGAAAGCACATGCAAAGTATTCTCACCCTGAGGAATATGCCAC
CanCV_RNA1_RdRp_304       CCGCTATTCTTTCAAACAGAAAGCACATGCAAAGTATTCTCACCCTGAAGAATATGCCAC
CanCV_RNA1_RdRp_306       CCGCTATTCTTTCAAACAGAAAGCACATGCAAAGTATTCTCACCCTGAAGAATATGCCAC
MT893743.1                CCGCTATTCTTTCAAACAGAAAGCACATGCAAAGTATTCTCACCCTGAAGAATATGCCAC
                          ************************************************ ***********

JN196536.1                ATACCCTTCCTCTAAAGGCTACTTCTACAATGCTACTTATGAGAATGCCCGCACTTTGGT
NC_031134.1               ATACCCTTCCTCTAAAGGCTACTTCTACAATGCTACTTATGAGAATGCCCGCACTTTGGT
CanCV_RNA1_RdRp_304       ATACCCTTCCTCTAAAGGCTACTTCTACAATGCTACTTATGAGAATGCCCGCACTTTGGT
CanCV_RNA1_RdRp_306       ATACCCTTCCTCTAAAGGCTACTTCTACAATGCTACTTATGAGAATGCCCGCACTTTGGT
MT893743.1                ATACCCTTCCTCTAAAGGCTACTTCTACAATGCTACTTATGAGAATGCCCGCACTTTGGT
                          ************************************************************

JN196536.1                TCACTACATCAAACAAAATGGTCTACCATTTGAATTTGATTTTAATCTCTCTGAAGAATC
NC_031134.1               TCACTACATCAAACAAAATGGTCTACCATTTGAATTTGATTTTAATCTCTCTGAAGAATC
CanCV_RNA1_RdRp_304       TCACTACATCAAACAAAATGGTCTACCATTTGAATTTGATTTTAATCTCTCTGAAGAATC
CanCV_RNA1_RdRp_306       TCACTACATCAAACAAAATGGTCTACCATTTGAATTTGATTTTAATCTCTCTGAAGAATC
MT893743.1                TCACTACATCAAACAAAATGGTCTACCATTTGAATTTGATTTTAATCTCTCTGAAGAATC
                          ************************************************************

JN196536.1                CCTGACCGACGAACAAATTGATTTATTCGTCAACAGATCCAATGCCTTTTTCAATGATTA
NC_031134.1               CCTGACCGACGAACAAATTGATTTATTCGTCAACAGATCCAATGCCTTTTTCAATGATTA
CanCV_RNA1_RdRp_304       CCTGACCGACGAACAAATTGATTTATTCGTCAACAGATCCAATGCCTTTTTCAATGATTA
CanCV_RNA1_RdRp_306       CCTGACCGACGAACAAATTGATTTATTCGTCAACAGATCCAATGCCTTTTTCAATGATTA
MT893743.1                CCTGACCGACGAACAAATTGATTTATTCGTCAACAGATCCAATGCCTTTTTCAATGATTA
                          ************************************************************

JN196536.1                CCCGACGTTACTATTCACTCGTAATCACATTTCGAAGCGTACTGGACCACTAAAAGTCCG
NC_031134.1               CCCGACGTTACTATTCACTCGTAATCACATTTCGAAGCGTACTGGACCACTAAAAGTCCG
CanCV_RNA1_RdRp_304       CCCGACGTTACTATTCACTCGTAATCACATTTCGAAGCGTACTGGACCACTAAAAGTCCG
CanCV_RNA1_RdRp_306       CCCGACGTTACTATTCACTCGTAATCACATTTCGAAGCGTACTGGACCACTAAAAGTCCG
MT893743.1                CCCGACGTTACTATTCACTCGTAATCACATTTCGAAGCGTACTGGACCACTAAAAGTCCG
                          ************************************************************

JN196536.1                CCCTGTTTACGCTGTTGATGATATTTTTATCATTATTGAACTGATGTTGACCTTCCCTCT
NC_031134.1               CCCTGTTTACGCTGTTGATGATATTTTTATCATTATTGAACTGATGTTGACCTTCCCTCT
CanCV_RNA1_RdRp_304       CCCTGTTTACGCTGTTGATGATATTTTTATCATTATTGAACTGATGTTGACCTTCCCTCT
CanCV_RNA1_RdRp_306       CCCTGTTTACGCTGTTGATGATATTTTTATCATTATTGAACTGATGTTGACCTTCCCTCT
MT893743.1                CCCTGTTTACGCTGTTGATGATATTTTTATCATTATTGAACTGATGTTGACCTTCCCTCT
                          ************************************************************

JN196536.1                CACTGGTCAAGCTCGAAAACCCTCATGCTGGA-TAAGGATGGC-TAGAAACCATTCG-GG
NC_031134.1               CACTGTTCAAGCTCGAAAACCCTCATGCTGTATTATGTACGGCTTCGAAACCATTCGTGG
CanCV_RNA1_RdRp_304       CACTGTTCAAGCTCGAAAACCCTCATGCTGTATTATGTATGGCTTCGAAACCATTCGTGG
CanCV_RNA1_RdRp_306       CACTGTTCAAGCTCGAAAACCCTCATGCTGTATTATGTATGGCTTCGAAACCATTCGTGG
MT893743.1                CACTGTTCAAGCTCGAAAACCCTCATGCTGTATTATGTATGGTCTAGAAACCATTCGTGG
                          ***** ************************ * ** * * **  * *********** **

JN196536.1                TTCAAACCGATACATTGACAGACTTGGTCGTTCGTATTCGACCTTCTTTTTCATAAACTG
NC_031134.1               CTCTAACCGATACATTGACAGACTTGCCCGTTCGTATTCGACCTTCTTTTCATTAGACTG
CanCV_RNA1_RdRp_304       TTCAAACCGATACATTGACAGACTTGCTCGTTCGTATTCGACCTTCTTTTCATTAGACTG
CanCV_RNA1_RdRp_306       TTCAAACCGATACATTGACAGACTTGCCCGTTCGTATTCGACCTTCTTTTCATTAGACTG
```

```
MT893743.1                TTCAAACCGATACATTGACAGACTTGCTCGTTCGTATTCGACCTTCTTTTCATTAGACTG
                           ** **********************  **********************   ** ****

JN196536.1                GGCAAGTTATGATCAACCAATGCCCCGTGTAATTACTGACATTTATTACACTGACTTCCT
NC_031134.1               GTCAAGTTATGATCAACGACTGCCCCGTGTAATTACTGACATTTATTACACTGACTTCCT
CanCV_RNA1_RdRp_304       GTCAAGTTATGATCAACGACTGCCCCGTGTAATTACTGACATTTATTACACTGACTTCCT
CanCV_RNA1_RdRp_306       GTCAAGTTATGATCAACGACTGCCCCGTGTAATTACTGACATTTATTACACTGACTTCCT
MT893743.1                GTCAAGTTATGATCAACGACTGCCCCGTGTAATTACTGACATTTATTACACTGACTTCCT
                          * *************** * ****************************************

JN196536.1                TCGAAGCCTGATCGTTATTAACCATGGATACCAACCCACGTATGAATACTCCTTTTACCC
NC_031134.1               TCGAAGCCTGATCGTTATTAACCATGGATACCAACCCACGTATGAATACTCCTCTTACCC
CanCV_RNA1_RdRp_304       TCGAAGCCTGATCGTTATTAACCATGGATACCAACCCACGTATGAATACTCCTCTTACCC
CanCV_RNA1_RdRp_306       TCGAAGCCTGATCGTTATTAACCATGGATACCAACCCACGTATGAATACTCCTCTTACCC
MT893743.1                TCGAAGCCTGATCGTTATTAACCATGGATACCAACCCACGTATGAATACTCCTCTTACCC
                          ***************************************************** ******

JN196536.1                TGACCTTGATGAACATAAGCTCTATGATAGAAAGAATAATCTTCTTTTCTTTCTTCATAC
NC_031134.1               TGACCTTGATGAACATAAGCTCTATGATAGAATGAATAATCTTCTTTTCTTTCTTCATAC
CanCV_RNA1_RdRp_304       TGACCTTGATGAACATAAGCTCTATGATAGAATGAATAATCTTCTTTTCTTTCTTCATAC
CanCV_RNA1_RdRp_306       TGACCTTGATGAACATAAGCTCTATGATAGAATGAATAATCTTCTTTTCTTTCTTCATAC
MT893743.1                TGACCTTGATGAACATAAGCTCTATGATAGAATGAATAATCTTCTTTTCTTTCTTCATAC
                          ******************************** ***************************

JN196536.1                ATGGTATAACAATATGACCTTCCTTCTCCCGGATGGTTTTGCATACCGCAGATCTTACTG
NC_031134.1               ATGGTATAACAATATGACCTTCCTTCTCCCGGATGGTTTTGCATACCGCAGATCTTACTG
CanCV_RNA1_RdRp_304       ATGGTATAACAATATGACCTTCCTTCTCCCGGATGGTTTTGCATACCGCAGATCTTACTG
CanCV_RNA1_RdRp_306       ATGGTATAACAATATGACCTTCCTTCTCCCGGATGGTTTTGCATACCGCAGATCTTACTG
MT893743.1                ATGGTATAACAATATGACCTTCCTTCTCCCGGATGGTTTTGCATACCGCAGATCTTACTG
                          ************************************************************

JN196536.1                TGGAGTCCCTTCTGGCCTCTACAATACTCAATACCTTGATTCTTTTGGCAATCTATTTTT
NC_031134.1               TGGAGTCCCTTCTGGCCTCTACAATACTCAATACCTTGATTCTTTTGGCAATCTATTTTT
CanCV_RNA1_RdRp_304       TGGAGTCCCTTCTGGCCTCTACAATACTCAATACCTTGATTCTTTTGGCAATCTATTTTT
CanCV_RNA1_RdRp_306       TGGAGTCCCTTCTGGCCTCTACAATACTCAATACCTTGATTCTTTTGGCAATCTATTTTT
MT893743.1                TGGAGTCCCTTCTGGCCTCTACAATACTCAATACCTTGATTCTTTTGGCAATCTATTTTT
                          ************************************************************

JN196536.1                AATAATTGATGCCATGATTGAATTCGGTTTCACTGACTCTGAAATCGATGGATTTGTCCT
NC_031134.1               AATAATTGATGCCATGATTGAATTCGGTTTCACTGACTCTGAAATCGATGGATTTGTCCT
CanCV_RNA1_RdRp_304       AATAATTGATGCCATGATTGAATTCGGTTTCACTGACTCTGAAATCGATGGATTTGTCCT
CanCV_RNA1_RdRp_306       AATAATTGATGCCATGATTGAATTCGGTTTCACTGACTCTGAAATCGATGGATTTGTCCT
MT893743.1                AATAATTGATGCCATGATTGAATTCGGTTTCACTGACTCTGAAATCGATGGATTTGTCCT
                          ************************************************************

JN196536.1                CCTAATTTTAGGTGATGACAACACTGGTATGACACAGATGCACATTCATCGCATCTCCCA
NC_031134.1               CCTAATTTTAGGTGATGACAACACTGGTATGACACAGATGCACATTCATCGCATCTCCCA
CanCV_RNA1_RdRp_304       CCTAATTTTAGGTGATGACAACACTGGTATGACACAGATGCACATTCATCGCATCTCCCA
CanCV_RNA1_RdRp_306       CCTAATTTTAGGTGATGACAACACTGGTATGACACAGATGCACATTCATCGCATCTCCCA
MT893743.1                CCTAATTTTAGGTGATGACAACACTGGTATGACACAGATGCACATTCATCGCATCTCCCA
                          ************************************************************

JN196536.1                ATTCATCAACTTTCTTGAAAAATATGCTTTAGAACGCTACAACATGGTCCTCTCTGCGAC
NC_031134.1               ATTCATCAACTTTCTTGAAAAATATGCTTTAGAACGCTATAACATGGTCCTCTCTGCGAC
CanCV_RNA1_RdRp_304       ATTCATCAACTTTCTTGAAAAATATGCTTTAGAACGCTACAACATGGTCCTCTCTGCGAC
CanCV_RNA1_RdRp_306       ATTCATCAACTTTCTTGAAAAATATGCTTTAGAACGCTACAACATGGTCCTCTCTGCGAC
MT893743.1                ATTCATCAACTTTCTTGAAAAATATGCTTTAGAACGCTACAACATGGTCCTCTCTGCGAC
                          ************************************** *********************

JN196536.1                CAAATCTGTTCTAACAACTCTGCGCTCAAAAATTGAGACCCTCGGTTACCAATGTAACTA
NC_031134.1               CAAATCTGTTCTAACAACTCTGCGCTCAAAAATTGAGACCCTCGGTTACCAATGTAACTA
CanCV_RNA1_RdRp_304       CAAATCTGTTCTAACAACTCTGCGCTCAAAAATTGAGACCCTTGGTTACCAATGTAACTA
CanCV_RNA1_RdRp_306       CAAATCTGTTCTAACAACTCTGCGCTCAAAAATTGAGACCCTCGGTTACCAATGTAACTA
MT893743.1                CAAATCTGTTCTAACAACTCTGCGCTCAAAAATTGAGACCCTCGGTTACCAATGTAACTA
                          ****************************************** *****************

JN196536.1                TGGATCCCCTAAACGCGACATTGATAAACTTGTTGCCCAACTCTGTTTCCCTGAAAATGG
NC_031134.1               TGGATCCCCTAAACGCGACATTGATAAACTTGTTGCCCAACTCTGTTTCCCTGAAAATGG
CanCV_RNA1_RdRp_304       TGGATCCCCTAAACGCGACATTGATAAACTCGTTGCCCAACTCTGTTTCCCTGAAAATGG
CanCV_RNA1_RdRp_306       TGGATCCCCTAAACGCGACATTGATAAACTTGTTGCCCAACTCTGTTTCCCTGAAAATGG
MT893743.1                TGGATCCCCTAAACGCGACATTGATAAACTTGTTGCCCAACTCTGTTTCCCTGAAAATGG
                          ****************************** *****************************

JN196536.1                ACTCAAGCCACACACCATGTCTGCTAGAGCTGTCGGAATCGCATATGCCGCTGCCGGCCA
NC_031134.1               ACTCAAGCCACACACCATGTCTGCTAGAGCTGTCGGAATCGCATATGCCGCTGCCGGCCA
CanCV_RNA1_RdRp_304       ACTCAAGCCACACACCATGTCTGCTAGAGCTGTCGGAATCGCATATGCCGCTGCCGGCCA
CanCV_RNA1_RdRp_306       ACTCAAGCCACACACCATGTCTGCTAGAGCTGTCGGAATCGCATATGCCGCTGCCGGCCA
MT893743.1                ACTCAAGCCACACACCATGTCTGCTAGAGCTGTCGGAATCGCATATGCCGCTGCCGGCCA
                          ************************************************************

JN196536.1                AGATTACGTATTTCACTCCTTTTGCCAGGATGTATATAATATGTTTAGGTCTTATTATAA
NC_031134.1               AGATTACGTATTTCACTCCTTTTGCCAGGATGTATATAATATGTTTAGGTCTTATTATAA
CanCV_RNA1_RdRp_304       AGATTACGTATTTCACTCCTTTTGCCAGGATGTATATAATATGTTTAGGTCTTATTATAA
CanCV_RNA1_RdRp_306       AGATTACGTATTTCACTCCTTTTGCCAGGATGTATATAATATGTTTAGGTCTTATTATAA
MT893743.1                AGATTACGTATTTCACTCCTTTTGCCAGGATGTATATAATATGTTTAGGTCTTATTATAA
                          ************************************************************

JN196536.1                ACCCGATGCTCGTGCAAATCTATTCTTCCAACGTCAAGTACTCCAAAATCTTGAAGATGG
NC_031134.1               ACCCGATGCTCGTGCAAATCTATTCTTCCAACGTCAAGTACTCCAAAATCTTGAAGATGG
CanCV_RNA1_RdRp_304       ACCCGATGCTCGTGCAAATCTATTCTTCCAACGTCAAGTACTCCAAAATCTTGAAGATGG
CanCV_RNA1_RdRp_306       ACCCGATGCTCGTGCAAATCTATTCTTCCAACGTCAAGTACTCCAAAATCTTGAAGATGG
MT893743.1                ACCCGATGCTCGTGCAAATCTATTCTTCCAACGTCAAGTACTCCAAAATCTTGAAGATGG
                          ************************************************************

JN196536.1                AATTCCTGATTTAGCCACACCGGTTGTGCCGCCATTCCCTTCATTATTCGAAGTCCGCGA
NC_031134.1               AATCCCTGATTTAGCCACACCGGTTGTGCCGCCATTCCCTTCATTATTCGAAGTCCGCGA
CanCV_RNA1_RdRp_304       AATTCCTGATTTAGCCACACCGGTTGTGCCGCCATTCCCTTCATTATTTGAAGTCCGCGA
CanCV_RNA1_RdRp_306       AATTCCTGATTTAGCCACACCGGTTGTGCCGCCATTCCCTTCATTATTCGAAGTCCGCGA
MT893743.1                AATTCCTGATTTAGCCACACCGGTTGTGCCGCCATTCCCTTCATTATTCGAAGTCCGCGA
                          *** ******************************************** ***********

JN196536.1                GATGTACTCTGAATACAAAGGACCTCTCTCTTTTGAACCTAAATGGAACAAAGCTCACTT
NC_031134.1               GATGTACTCTGAATACAAAGGACCTCTCTCTTTTGAACCTAAATGGAACAAAGCTCACTT
CanCV_RNA1_RdRp_304       GATGTACTCTGAATATAAAGGACCTCTCTCTTTTGAACCTAAATGGAACAAAGCTCACTT
CanCV_RNA1_RdRp_306       GATGTACTCTGAATACAAAGGACCTCTCTCTTTTGAACCTAAATGGAACAAAGCTCACTT
MT893743.1                GATGTACTCTGAATACAAAGGACCTCTCTCTTTTGAACCTAAATGGAACAAAGCTCACTT
                          *************** ********************************************

JN196536.1                CATAAATGACCCGAATGACATCCCTCCTTTCTCCAAAACTATGCGAGACTATGAAAAGGA
NC_031134.1               CATAAATGACCCGAATGACATCCCTCCTTTCTCCAAAACTATGCGAGACTATGAAAAGGA
CanCV_RNA1_RdRp_304       CATAAATGACCCGAATGACATCCCTCCTTTCTCCAAAACTATGCGAGACTATGAAAAGGA
CanCV_RNA1_RdRp_306       CATAAATGACCCGAATGACATCCCTCCTTTCTCCAAAACTATGCGAGACTATGAAAAGGA
MT893743.1                CATAAATGACCCGAATGACATCCCTCCTTTCTCCAAAACTATGCGAGACTATGAAAAGGA
                          ************************************************************

JN196536.1                ACATAATCTTCCTGTTCGAGTTTCACCTACTTTTGAAACGGTCGTGCCTAGCACAAAAAA
NC_031134.1               ACATAATCTTCCTGTTCGAGTTTCACCTACTTTTGAAACGGTCGTGCCTAGCACAAAAAA
CanCV_RNA1_RdRp_304       ACATAATCTTCCTGTTCGAGTTTCACCTACTTTTGAAACGGTCGTGCCTAGCACAAAAAA
CanCV_RNA1_RdRp_306       ACATAATCTTCCTGTTCGAGTTTCACCTACTTTTGAAACGGTCGTGCCTAGCACAAAAAA
MT893743.1                ACATAATCTTCCTGTTCGAGTTTCACCTACTTTTGAAACGGTCGTGCCTAGCACAAAAAA
                          ************************************************************

JN196536.1                TTTGCCGTAATTTTTTATCTTCGTTTTTAAATTTTCGTTGATTTTGCAAATTTATATAAA
```

```
NC_031134.1               TTTGCCGTAATTTTTTATCTTCGTTTTTAAATTTTCGTTGATTTTGCAAATTTATAT---
CanCV_RNA1_RdRp_304       TTTGCCGTAATTTTTTATCTTCGTTTTTAAATTTTCGTTGATTTTGCAAAT---------
CanCV_RNA1_RdRp_306       TTTGCCGTAATTTTTTATCTTCGTTTTTAAATTTTCGTTGATTTTGCAAAT---------
MT893743.1                TTTGCCG-----------------------------------------------------
                          *******

JN196536.1                AAAAAAAAAAAAAAAAAAAAAAAA
NC_031134.1               ------------------------
CanCV_RNA1_RdRp_304       ------------------------
CanCV_RNA1_RdRp_306       ------------------------
MT893743.1                ------------------------


---------------

CanCV_RNA2_CP_304       AGATTTTTCTAAAGCGCCCCGCCTTTAGTTATTAAGAATTTACATTCGTCTATATTATCA
JN196537.1              -GATTTTTCTAAAGCGCCCCGCCTTTAGTTATTAAGAATTTACATTCGTCTATATTATCA
CanCV_RNA2_CP_306       AGATTTTTCTAAAGCGCCCCGCCTTTAGTTATTAAGAATTTACATTCGTCTATATTATCA
NC_031130.1             AGATTTTTCTAAAGCGCCCCGCCTTTAGTTATTGAGAATTTACATTCGTCTATATTATCA
                         ******************************** **************************

CanCV_RNA2_CP_304       AGTCCTCGAAAAACCTCTGCTCTAATTTTAAGATGTCTTCCTCAACCATCACTTCCCGTC
JN196537.1              AGTCCTCGAAAAACCTCTGCTCTAATTTTAAGATGTCTTCCTCAACCATCACTTCCCGTC
CanCV_RNA2_CP_306       AGTCCTCGAAAAACCTCTGCTCTAATTTTAAGATGTCTTCCTCAACCATCACTTCCCGTC
NC_031130.1             AGTCCTCGAAAAACCTCTGCTCTAATTTTAAGATGTCTTCCTCAACCATCACTTCCCGTC
                        ************************************************************

CanCV_RNA2_CP_304       TCGATGCCCTCAAAAGCAAGAACGTGATAATTGAATTCAAAGAAGCGAAAACTCCTGTCC
JN196537.1              TCGATGCCCTCAAAAGCAAGAACGTGATAATTGAATTCAAAGAAGCGAAAACTCCTGTCC
CanCV_RNA2_CP_306       TCGATGCCCTCAAAAGCAAGAACGTGATAATTGAATTCAAAGAAGCGAAAACTCCTGTCC
NC_031130.1             TCGATGCCCTCAAAAGCAAGAACGTGATAATTGAATTCAAAGAAGCGAAAACTCCTGTCC
                        ************************************************************

CanCV_RNA2_CP_304       CCTCTGCTGACATTTTGCAACTCTTTGCTGACAACACCGCCGCCCGCTCTGACTCATCCA
JN196537.1              CCTCTGCTGACATTTTGCAACTCTTTGCTGACAACACCGCCGCCCGCTCTGACTCACCCA
CanCV_RNA2_CP_306       CCTCTGCTGACATTTTGCAACTCTTTGCTGACAACACCGCCGCCCGCTCTGACTCATCCA
NC_031130.1             CCTCTGCTGACATTTTGCAACTCTTTGCTGACAACACCGCCGCCCGCTCTGACTCATCCA
                        ******************************************************** ***

CanCV_RNA2_CP_304       ATGAATGGATTATTGATGTTTTCCCAAACTTCACTCCCATCCTGATGTACATTATGCTCA
JN196537.1              ATGAATGGATTATTGATGTTTTCCCAAACTTCACTCCCATCCTGATGTACATTATGCTCA
CanCV_RNA2_CP_306       ATGAATGGATTATATATGTTTTCCCAAACTTCACTCCCATCCTGATGTACATTATGCTCA
NC_031130.1             ATGAATGGATTATTGATGTTTTCCCAAACTTCACTCCCATCCTGATGTACATTATGCTCA
                        *************  *********************************************

CanCV_RNA2_CP_304       ATGCGATTCGCCACGCGAACGCCACTGCTCAACGTGAACATTCCAAGTCATCTGTTGCCA
JN196537.1              ATGCGATTCGCCACGCGAACGCCACTGCTCAACGTGAACATTCCAAGTCATCTGTTGCCA
CanCV_RNA2_CP_306       ATGCGATTCGCCACGCGAACGCCACTGCTCAACGTGAACATTCCAAGTCATCTGTTGCCA
NC_031130.1             ATGCGATTCGCCACGCGAACGCCACTGCTCAACGTGAACATTCCAAGTCATCTGTTGCCA
                        ************************************************************

CanCV_RNA2_CP_304       CCATTTGCATGTATCATCTGTCCATTGTCTATGGATTTTTCCTCATCAATGACCTCAATG
JN196537.1              CCATTTGCATGTATCATCTGTCCATTGTCTATGGATTTTTCCTCATCAATGACCTCAATG
CanCV_RNA2_CP_306       CCATTTGCATGTATCATCTGTCCATTGTCTATGGATTTTTCCTCATCAATGACCTCAATG
NC_031130.1             CCATTTGCATGTATCATCTGTCCATTGTCTATGGATTTTTCCTCATCAATGACCTCAATG
                        ************************************************************

CanCV_RNA2_CP_304       TCCGTCCTGCTCCGTCTGCTCATGCCCGTATCTGGGCCGAATCCAACTGGAGATCTGAAT
JN196537.1              TCCGTCCTGCTCCGTCTGCTCATGCCCGTATCTGGGCCGAATCCAACTGGAGATCTGAAT
CanCV_RNA2_CP_306       TCCGTCCTGCTCCGTCTGCTCATGCCCGTATCTGGGCCGAATCCAACTGGAGATCTGAAT
NC_031130.1             TCCGTCCTGCTCCGTCTGCTCATGCCCGTATCTGGGCCGAATCCAACTGGAGATCTGAAT
                        ************************************************************

CanCV_RNA2_CP_304       TTGTGAAATTTCTGTCCACCTTGCCTGTACCTGAGTTCCTTATTCCCATCCTATCCCAGT
JN196537.1              TTGTGAAATTTCTGTCCACCTTGCCTGTACCTGAGTTCCTTATTCCCATCCTATCCCAGT
CanCV_RNA2_CP_306       TTGTGAAATTTCTGTCCACCTTGCCTGTACCTGAGTTCCTTATTCCCATCCTATCCCAGT
NC_031130.1             TTGTGAAATTTCTGTCCACCCTGCCTGTACCTGAGTTCCTTATTCCCATCCTATCCCAGT
                        ******************** ***************************************

CanCV_RNA2_CP_304       TTCACGCCTTTGAAACCGCAAGAACAAAGAATGTTTTCTTTATTCCCTCTGCCGCCGGCT
JN196537.1              TTCACGCCTTTGAAACCGCAAGAACAAAGAATGTTTTCTTTATTCCCTCTGCCGCCGGCT
CanCV_RNA2_CP_306       TTCACGCCTTTGAAACCGCAAGAACAAAGAATGTTTTCTTTATTCCCTCTGCCGCCGGCT
NC_031130.1             TTCACGCCTTTGAAACCGCAAGAACAAAGAATGTTTTCTTTATTCCCTCTGCCGCCGGCT
                        ************************************************************

CanCV_RNA2_CP_304       ATGACCACGACCAATTCTTCGGACGTGTTTATCCTTTGAATATGTTCGCCGCAATTCATG
JN196537.1              ATGACCACGACCAATTCTTCGGACGTGTTTATCCTTTGAATATGTTCGCCGCAATTCATG
CanCV_RNA2_CP_306       ATGACCACGACCAATTCTTCGGACGTGTTTATCCTTTGAATATGTTCGCCGCAATTCATG
NC_031130.1             ATGACCACGACCAATTCTTCGGACGTGTTTATCCTTTGAATATGTTCGCCGCAATTCATG
                        ************************************************************

CanCV_RNA2_CP_304       ACTGTACTGCCACACTGCCCGGAAACTCCTCCAAAATCGATGTCCTTAGAGACCTCTACT
JN196537.1              ACTGTACTGCCACACTGCCCGGAAACTCCTCCAAAATCGATGTCCTTAGAGACCTCTACT
CanCV_RNA2_CP_306       ACTGTACTGCCACACTGCCCGGAAACTCCTCCAAAATCGATGTCCTTAGAGACCTCTACT
NC_031130.1             ACTGTACTGCCACACTGCCCGGAAACTCCTCCAAAATCGATGTCCTTAGAGACCTCTACT
                        ************************************************************

CanCV_RNA2_CP_304       CCAGAGTACTCTACTCAATTACTGACCCAGCCTACACCTGCGTCATTCCCGATCTTATTG
JN196537.1              CCAGAGTACTCTACTCAATTACTGACCCAGCCTACACCTGCGTCACTCCCGATCTTATTG
CanCV_RNA2_CP_306       CCAGAGTACTCTACTCAATTACTGACCCAGCCTACACCTGCGTCATTCCCGATCTTATTG
NC_031130.1             CCAGAGTACTCTACTCAATTACTGACCCAGCCTACACCTGCGTCATTCCCGATCTTATTG
                        ********************************************* **************

CanCV_RNA2_CP_304       GAATCACCCTTAGCGCTGCTGACATTACCCAAGCTCAATACATGAATTCAAAACTTTACC
JN196537.1              GAATCACCCTTAGCGCTGCTGACATTACCCAAGCTCAATACATGAATTCAAAACTTTACC
CanCV_RNA2_CP_306       GAATCACCCTTAGCGCTGCTGACATTACCCAAGCTCAATACATGAATTCAAAACTTTACC
NC_031130.1             GAATCACCCTTAGCGCTGCTGACATTACCCAAGCTCAATACATGAATTCAAAACTTTACC
                        ************************************************************

CanCV_RNA2_CP_304       AAGTTTTCTCTTCTGTTATTAATCCTGTCCTGTTCCGCGACTTTCAGCGCCGATCCTCCC
JN196537.1              AAGTTTTCTCTTCTGTTATTAATCCTGTCCTGTTCCGCGACTTTCAGCGCCGATCCTCCC
CanCV_RNA2_CP_306       AAGTTTTCTCTTCTGTTATTAATCCTGTCCTGTTCCGCGACTTTCAGCGCCGATCCTCCC
NC_031130.1             AAGTTTTCTCTTCTGTTATTAATCCTGTCCTGTTCCGCGACTTTCAGCGCCGATCCTCCC
                        ************************************************************

CanCV_RNA2_CP_304       TCGCCGCTCTATCATTCAAAGCTCCAACGTTTGCCTCTGCTAACGTTAACGCTTATGATC
JN196537.1              TCGCCGCTCTATCATTCAAAGCTCCAACGTTTGCCTCTGCTAACGTTAACGCTTATGATC
CanCV_RNA2_CP_306       TCGCCGCTCTATCATTCAAAGCTCCAACGTTTGCCTCTGCTAACGTTAACGCTTATGATC
NC_031130.1             TCGCCGCTCTATCATTCAAAGCTCCAACGTTTGCCTCTGCTAACGTTAACGCTTATGATC
                        ************************************************************

CanCV_RNA2_CP_304       TGATGTTTTCAGCCACTTCCGCTAATCTGCGTGAATTAAAGGTTGTCCTACAATCAGTCC
JN196537.1              TGATGTTTTCAGCCACTTCCGCTAATCTGCGTGAATTAAAGGTTGTCCTACAATCAGTCC
CanCV_RNA2_CP_306       TGATGTTTTCAGCCACTTCCGCTAATCTGCGTGAATTAAAGGTTGTCCTACAATCAGTCC
NC_031130.1             TGATGTTTTCAGCCACTTCCGCTAATCTGCGTGAATTAAAGGTTGTCCTACAATCAGTCC
                        ************************************************************

CanCV_RNA2_CP_304       ATGCCGTTATCTCTGATGATGTATCATGTAAGACCAGCCTTGGTCAATTTATTGCTGATG
JN196537.1              ATGCCGTTATCTCTGATGATGTATCATGTAAGACCAGCCTTGGTCAATTTATTGCTGATG
CanCV_RNA2_CP_306       ATGCCGTTATCTCTGATGATGTATCATGTAAGACCAGCCTTGGTCAATTTATTGCTGATG
NC_031130.1             ATGCCGTTATCTCTGATGATGTATCATGTAAGACCAGCCTTGGTCAATTTATTGCTGATG
                        ************************************************************
```

```
CanCV_RNA2_CP_304      CTACCACCTCAACAATCATTAAACATGGATACTCCACATACGCTCTTCCCACCTGGTCCC
JN196537.1             CTACCACCTCAACAATCATTAAACATGGATACTCCACATATGCTCTTCCCACCTGGTCCC
CanCV_RNA2_CP_306      CTACCACCTCAACAATCATTAAACATGGATACTCCACATATGCTCTTCCCACCTGGTCCC
NC_031130.1            CTACCACCTCAACAATCATTAAACATGGATACTCCACATACGCTCTTCCCACCTGGTCCC
                       **************************************** *******************

CanCV_RNA2_CP_304      ACAGTGAAGTCACCAACAAAGCTCAAACCTTTCGTGCCATTACTGCCCAACAACTAAT
JN196537.1             ACAGTGAAGTCACCAACAAAGCTCAAACCTTTCGTGCCATTACTGCCCAACAACTAAT
CanCV_RNA2_CP_306      ACAGTGAAGTCACCAACAAAGCTCAAACCTTTCGTGCCATTACTGCCCAACAACTAAT
NC_031130.1            ACAGTGAAGTCACCAACAAAGCTCAAACCTTTCGTGCCATTACTGCCCAACAACTAAT
                       **********************************************************

CanCV_RNA2_CP_304      CTCTGAAGATGCCCGTGCCCAAGACTTCTGTTTCCTTCAACGCCCTACAGCTGAAATTCC
JN196537.1             CTCTGAAAATGCCCGTGCCCAAGAATCCTGTTTCCTTCAACGCCCTACAGCTGAAATTCC
CanCV_RNA2_CP_306      CTCTGAAGATGCCCGTGCCCAAGACTTCTGTTTCCTTCAACGCCCTACAGCTGAAATTCC
NC_031130.1            CTCTGAAGATGCCCGTGCCCAAGACTTCTGTTTCCTTCAACGCCCTGCAGCTGAAATTCC
                       ******* **************** * ******************* *************

CanCV_RNA2_CP_304      TCACACCCACAACCTCGAAGAC-GTACGTTACGTACGCACCGATGCCCCCACTGAACCAG
JN196537.1             TCAACCCCACAACCTCGGAAACAGTACGTTACGTACGCACCGATGCCCCCACTGGA-CAG
CanCV_RNA2_CP_306      TCACACCCACAACCTCGAAGAC-GTACGTTACGTACGCACCGATGCCCCCACTGAACCAG
NC_031130.1            TCACACCCACAACCTCGAAGAC-GTACGTTACGTACGCACCGATGCCCCCACTGAACCAG
                       ***  ************ * ** ******************************* * ***

CanCV_RNA2_CP_304      TCGCTCTGCCCCAGAATCACAGTATCATCCGTAACTTCCCTCTCTGCCTCAGAAACAATA
JN196537.1             TCCCTTTGCCCCAGAATCACAGTATCATCCGTAACTTCCCTCTCTGCCTCAGAAACAATA
CanCV_RNA2_CP_306      TCGCTCTGCCCCAGAATCACAGTATCATCCGTAACTTCCCTCTCTGCCTCAGAAACAATA
NC_031130.1            TCGCTCTGCCCCAGAATCACAGTATCATCCGTAACTTCCCTCTCTGCCTCAGAAACAATG
                       ** ** *****************************************************

CanCV_RNA2_CP_304      CGAATGCCGCCGTCTTTCCCCGCGTAGACAACAACACACTGATCAATTTTAGTGAAGAAA
JN196537.1             CGAATGCCGCCGTCTTTCCCCGCGTAGACAACAACACACTGATCAATTTTAGTGAAGAAA
CanCV_RNA2_CP_306      CGAATGCCGCCGTCTTTCCCCGCGTAGACAACAACACACTGATCAATTTTAGTGAAGAAA
NC_031130.1            CGAATGCCGCCGTCTTTCCCCGCGTAGACAACAACACACTGATCAATTTTAGTGAAGAAA
                       ************************************************************

CanCV_RNA2_CP_304      TCCACACTGTTCCAAGCGTCTTAGTGCTCGACACCGATGGTGATCAAACCATCACTGCCC
JN196537.1             TCCACACTGTTCCAAGCGTCTTAGTGCTCGACACCGATGGTGATCAAACCATCACTGCCC
CanCV_RNA2_CP_306      TCCACACTGTTCCAAGCGTCTTAGTGCTCGACACCGATGGTGATCAAACCATCACTGCCC
NC_031130.1            TCCACACTGTTCCAAGCGTCTTAGTGCTCGACACCGATGGTGATCAAACCATCACTGCCC
                       ************************************************************

CanCV_RNA2_CP_304      ACTTGCCACTACTTGCCGGAAAGATCATTGAATCTTTTGAACTCGATGGAACCACCATCG
JN196537.1             ACCTGCCACTACTTGCCGGAAAGATCATTGAATCTTTTGAACTCGATGGAACCACTATCG
CanCV_RNA2_CP_306      ACCTGCCACTACTTGCCGGAAAGATCATTGAATCTTTTGAACTCGATGGAACCACTATCG
NC_031130.1            ACCTGCCACTACTTGCCGGAAAGATCATTGAATCTTTTGAACTCGATGGAACCACTATCG
                       ** **************************************************** ****

CanCV_RNA2_CP_304      AAATGCCCAATGCCATGAAATCACTCGGCATGCAAAACTGCATGTTCGCTGACTCTGCCA
JN196537.1             AAATGCCCAATGCCATGAAATCACTCGGCATGCAAAACTGCATGTTCGCTGACTCTGCCA
CanCV_RNA2_CP_306      AAATGCCCAATGCCATGAAATCACTCGGCATGCAAAACTGCATGTTCGCTGACTCTGCCA
NC_031130.1            AAATGCCCAATGCCATGAAATCACTCGGCATGCAAAACTGCATGTTCGCTGACTCTGCCA
                       ************************************************************

CanCV_RNA2_CP_304      TTCCGTACAAGTATGTTCGCCCTGGCTCCGCTTACCATCCCGTTAATGCTGGAACCATAC
JN196537.1             TTCCGTACAAGTATGTTCGCCCTGGCTCCGCTTACCATCCCGTTAATGCTGGAACCATAC
CanCV_RNA2_CP_306      TTCCGTACAAGTATGTTCGCCCTGGCTCCGCTTACCATCCCGTTAATGCTGGAACCATAC
NC_031130.1            TTCCGTACAAGTATGTTCGCCCTGGCTCCGCTTACCATCCCGTTAATGCTGGAACCATAC
                       ************************************************************

CanCV_RNA2_CP_304      TGCCGCCCCGTTCTCGAGCTACTCCCAATTCCACTCCTCGCCTGCCTGCCTCGTCGTTCC
JN196537.1             TGCCGCCCCGTTCTCGAGCTACTCCCAATTCCACTCCTCGCCTGCCTGCCTCGTCGTTCC
CanCV_RNA2_CP_306      TGCCGCCCCGTTCTCGAGCTACTCCCAATTCCACTCCTCGCCTGCCTGCCTCGTCGTTCC
NC_031130.1            TGCCGCCCCGTTCTCGAGCTACTCCCAATTCCACTCCTCGCCTGCCTGCCTCGTCGTTCC
                       ************************************************************

CanCV_RNA2_CP_304      TATACAATCGACTGCGTGTTAATCTCCCCCGATTCAACCTCGCTATCAATGAAGAACGCA
JN196537.1             TATACAATCGACTGCGTGTTAATCTCCCCCGATTCAACCTCGCTATCAATGAAGAACGCA
CanCV_RNA2_CP_306      TATACAATCGACTGCGTGTTAATCTCCCCCGATTCAACCTCGCTATCAATGAAGAACGCA
NC_031130.1            TATACAATCGACTGCGTGTTAATCTCCCCCGATTCAACCTCGCTATCAATGAAGAACGCA
                       ************************************************************

CanCV_RNA2_CP_304      TTGGCAACATTCTCCCAGGAATGACTCTACTTGATGGAGTCACTGTCCTCCGGTATGTTC
JN196537.1             TTGGCAACATTCTCCCAGGAATGACTCTACTTGATGGAGTCACTGTCCTCCGGTATGTTC
CanCV_RNA2_CP_306      TTGGCAACATTCTCCCAGGAATGACTCTACTTGATGGAGTCACCGTCCTCCGGTATGTTC
NC_031130.1            TTGGCAACATTCTCCCAGGAATGACTCTACTTGATGGAGTCACTGTCCTCCGGTATGTTC
                       ******************************************* ****************

CanCV_RNA2_CP_304      AGTCCTTTCTAGGATTCAACACCGTCAGCCGTGCTGACAATGCCGACAATGGAGACAATG
JN196537.1             AGTCCTTTCTAGGATTCAACACCGTCAGCCGTGCTGACAATGCCGATAATGGAGACAATG
CanCV_RNA2_CP_306      AGTCCTTTCTAGGATTCAACACCGTCAGCCGTGCTGACAATGCCGATAATGGAGACAATG
NC_031130.1            AGTCCTTTCTAGGATTCAACACCGTCAGCCGTGCTGACAATGCCGATAATGGAGACAATG
                       ********************************************* **************

CanCV_RNA2_CP_304      TCCCGGCGATGCCCCGGAACCTGCTTCTGGTCTGGTCCCCGTACACGTACACTCCGTATG
JN196537.1             TCCCGGCGATGCCCCGGAACCTGCTTCTGGTCTGGTCCCCGTACACGTACACTCCGTATG
CanCV_RNA2_CP_306      TCCCGGCGATGCCCCGGAACCTGCTTCTGGTCTGGTCCCCGTACACGTACACTCCGTATG
NC_031130.1            TCCCGGCGATGCCCCGGAACCTGCTTCTGGTCTGGTCCCCGTACACGTACACTCCGTATG
                       ************************************************************

CanCV_RNA2_CP_304      AATCTGATGACCTGCCTGAACCAGCACTTGGCGAATCAAGGCACTACTACTTGACAAACC
JN196537.1             AATCTGATGACCTGCCTGAACCAGCACTTGGCGAATCAAGGCACTACTACTTGACAAACC
CanCV_RNA2_CP_306      AATCTGATGACCTGCCTGAACCAGCACTTGGCGAATCAAGGCACTACTACTTGACAAACC
NC_031130.1            AATCTGATGACCTGCCTGAACCAGCACTTGGCGAATCAAGGCACTACTACTTGACAAACC
                       ************************************************************

CanCV_RNA2_CP_304      TCCGTACCATCTTCGGAACGGACTACAATCTTGTTCAAGCCAAGCATCCCTATGAATCGC
JN196537.1             TCCGTACCATCTTCGGAACGGACTACAATCTTGTTCAAGCCAAGCATCCTTATGAATCGC
CanCV_RNA2_CP_306      TCCGTACCATCTTCGGAACGGACTACAATCTTGTTCAAGCCAAGCATCCTTATGAATCGC
NC_031130.1            TCCGTACCATCTTCGGAACGGACTACAACCTTGTTCAAGCCAAGCATCCCTATGAATCGC
                       **************************** ******************** **********

CanCV_RNA2_CP_304      TCCCCGTCGTTTAAGCAATCTCTCTCATCCTCTTAATGTTTAAGTTTTATGATGTTTGAT
JN196537.1             TCCCCGTCGTTTAAGCAATCTCTCTCATCCTCTTAATGTTTAAGTTTTATGATGTTTGAT
CanCV_RNA2_CP_306      TCCCCGTCGTTTAAGCAATCTCTCTCATCCTCTTAATGTTTAAGTTTTATGATGTTTGAT
NC_031130.1            TCCCCGTCGTTTAAGCAATCTCTCTCATCCTCTTAATGTTTAAGTTTTATGATGTTTGAT
                       ************************************************************

CanCV_RNA2_CP_304      TGGCCTCCTTTTCCGACAAACACGTGTATGCTACGTGTTTTGTTTTTTTTCGTTTTATTT
JN196537.1             TGGCCTCCTTTTCCGACAAACACGTGTATGCTACGTGTTTTGTTTTTTTTCGTTTTATTT
CanCV_RNA2_CP_306      TGGCCTCCTTTTCCGACAAACACGTGTATGCTACGTGTTTTGTTTTTTTTCGTTTTATTT
NC_031130.1            TGGCCTCCTTTTCCGACAAACACGTGTATGCTACGTGTTTTGTTTTTTTTCGTTTTATTT
                       ************************************************************

CanCV_RNA2_CP_304      CCTGAACTTTTATTTTCAGTCTTTTTTAACGTTATTTTCTACTTTAAG-----------
JN196537.1             CCTGAACTTTTATTTTCAGTCTTTTTTAACGTTATTTTCTACTTTAAGAAAAAAAAAAA
CanCV_RNA2_CP_306      CCTGAACTTTTATTTTCAGTCTTTTTTAACGTTATTTTCTACTTTAAG-----------
NC_031130.1            CCTGAACTTTTATTTTCAGTCTTTTTTAACGTTATTTTCTACTTTAAG-----------
                       ************************************************

CanCV_RNA2_CP_304      --------------
JN196537.1             AAAAAAAAAAAAAA
CanCV_RNA2_CP_306      --------------
```

NC_031130.1   --------------

(B) Cannabis partiti-like virus (CanPLV)

>CanPLV NewPartiti_315
CCGTTTTACCAACGCGGAAAGCTTACACTTTCATACCTTGCGTTTAATGTTTTATACGTAAGCAAGCCGG**ATG**GCGTATCGCACTACTTCAAGTCACGGGACTTCAACTTCATATTCTTCATTTCGACGGCCTAAAGCGATATCGCGATT
ACCCCGTAGTCCCGGGTTCGCATTTGATCTGTCTCAACCTCGAGAAGATCCACTAGCGCGACGTGCTGTTGAGCATGTCTTTGGGAAAGCCTATGTTGACTCGTGCTTCGCTCGTCTCCATCGAAGTACTATGTCCTATGAGGCGCTGGT
GTCTGACTTATTCGAGTTTGATCGGTTGCTTCCCAATCGCCCAGTCGATGATCCAGTCTATGCGATGGTTCTCGAGTCGGTGAGGCAGGACTTACACGCACCGAGATTACTCGTACCACATACTATCGGAGCGGTGACCAAACTTCCCGA
CTTTCCCCGGCAGAAGTCTCCAGGTTTGCCGTATAAGAACATGGGTTATAAGACCAAAGGTGAGGTTGTCGATGACACCTACCAGTTAGAGGAGATTAACAGGCGCTGGCACTACATTGGCTTCGGGAGTGATCGAGGACCGTTACCGGA
TACCTGTCTATTCGCAAGATCTCAGGTATGTGACGTGTCCAAGCAAAAGGTTCGGGGCGTTTGGGGCTATCCCCTTTTCGTCTATCTTGAAGAAGCCCGATTCTTCTATCCGATACTGGAGCACCTAAAGAGTAAAGGTCATAACTTCCC
AATTGGCTATGGTTGTGAAATCAGTAAGGGTGGTCTTGTCGCCATATCCGAGATGGCGCTTCGTGTGCGCGGTGGCAAGTATATGATGACTGATTGGAGTAAATTTGATAAATCTATACCTCCTTGGCTGATTCGGGATGCCTTTTCTCT
TTTGGCTGAATTAATTGACTTCGACCACGTTGTTGATTCCGAGGGTTTGCAATGGCATGTCCGAGGATATCGTAGCAAGGCACGTTGGAAGAAGATTGTCGACTATTTTGTCGAGACTCCAGTCCGTACCTGTAAGGGTGAGCGTTTCCT
TGTTAGATCTGGTGTGCCTTCGGGGTCGTGCTTCACGAACGTTATTGATACTATCATTAACTGTATTGTTACCCGGTACATCTCTTATAATACTGTGGGTTCTTTTCCTCTTGGTGAAATGTACCTTGGGGACGATGGTGTGTGTGTTTT
CTCTTCGTCCAGTCTGATTGATCTGCGTAGTATGGCCGCTCTTGCTCACGAGCGCTTTGGCCTTGTTCTTAGCCCCGATAAATCATATGTTACTACCCAAGCTGAGAACATCCACTTCCTTGGTTACTACAATAGGCCCATCGGCATGCC
TAACAGAGATCAGGCTCTTCTGATTGTATCGTTTGTTCATCCTGAGAAAAGTCGCGAGACTGCTGTCGAGTGCGCTGCTGCTGCGCTTGGCCAGTTGTGGGCAAATTTTGATCCTCTAATTGCTGAGTCATGGTTTAGGGTCCTGTGCTA
TGTCTCAAACAGTTACGAGGTCGAATTCGGTGACGTTCTCATCAAGTTGCGTCAGACTGCGCATAGACACAAGTATTTATCCCATATCGGTCGTAGCTTGAGCCAGCTCACTGTTCCAACCCCGTGTGAGTTTGGCGGTATTTTAGAAAT
CTATGCTTCTCCTGTTCCCTCTCGCTACTGCGATTTGAGGTACTGGGACTACGGAGACTTATATAGGAGGGCTAGGTTAAGGTGGTTGGAGGCCCTTGATCTGGGGGAT**TAA**TTGAATTCCAGATTTGACGTAGTGTGGAAAGACACCAA
CTGCAAGGTCTACAGGACCCTGCAGGGATTCT

>CanPLV NewPartiti_309
ccgtTTTACCAACGCGGGAAGCTTACACTTTTATACCTTGCGTTTAATGTTTTATACGTAAGCAAGCCGG**ATG**GCGTATCGCACTACTTCAAGTCACGGGACTTCAACTTCATATTCTTCATTTCGACGGCCAAAAGCGATATCGCGATT
ACCCCGTAGTCCCGGGTTCGCATTTGATCTGTCTCAACCTCGAGAAGATCCACTAGCGCGACGTGCAGTTGAGCATGTCTTTGGGAAAGCCTATGTTGACTCGTGCTTCGCTCGTCTCCATCGAAGTACTATGTCCTATGAGGCGCTGGT
GTCTGACTTATTCGAGTTTGATCGGTTGCTTCCtTGTCGCCCAGTCGATGATCCAGTCTATGCGATGGTTCTCGAGTCTGTGAGGCAGGACTTACACGCACCGAGATTACTCGTACCACATACTATCGGAGCGGTGACCAAACTTCCCGA
CTTTCCCCGGCAGAAGTCTCCGGGTTTGCCGTATAAGAACATGGGTTATAAGACCAAAGGTGAGGTTGTCGATGACACCTACCAGTTAGAGGAGATTAACAGGCGCTGGCACTACATTGGCTTCGGGCGTGATCGAGGGCCGTTACCGGA
TACCTGCCTATTTGCAAGATCTCAGGTATGTGACGTGTCCAAGCAAAAGGTTCGGGGCGTTTGGGGTTATCCCCTTTTCGTCTATCTTGAAGAAGCCCGATTCTTCTATCCGATACTGGAGCACCTAAAGAGTAAGGGTCATAACTTCCC
AATTGGCTATGGTTGTGAAATCAGTAAGGGTGGTCTTGTCGCCATATCCGAGATGGCGCTTCGTGTGCGCGGTGGCAAGTATATGATGACTGATTGGAGTAAATTTGACAAATCCATACCTCCTTGGCTGATTCGGGATGCCTTTTCTCT
TTTGGCTGAATTAATTGACTTCGACCACGTTGTTGATTCCGAGGGTTTGCAGTGGCATGTCCGAGGATATCGTAGCAAGGCACGTTGGAAGAAGATTGTCGACTATTTTGTCGAGACTCCAGTCCGTACCTGTAAGGGCGAGCGTTTCCT
TGTTAGATCTGGTGTACCGTCGGGATCGTGCTTCACGAACGTTATTGATACCATCATTAACTGTATTGTTACCCGGTACATCTCTTATAATACTGTGGGTTCTTTTCCTCTTGGTGAAATGTACCTTGGGGACGATGGTGTGTGTGTTTT
CTCTTCGTCCAGTCTGGTTGATCTGAGTAGTATGGCCGCTCTTGCTCACGAGCGCTTTGGCCTTGTTCTTAGTCCTGACAAATCATATGTTACTACCCAAGCTGAGAACATCCACTTCCTTGGATACTACAATAGGccCATCGGCATGCC
TAACAGAGATCAGGCTCTTCTAATTGTATCGTTTGTTCATCCTGAGAAAAGTCGCGAGACTGCTGTCGAGTGCGCTGCTGCTGCGCTTGGCCAGTTGTGGGCAAATTTTGATCCTCTAATTGCTGAGTCATGGTTTAGGGTCCTGTGCTA
TGTCTCAAACAGTTATGAGGTCGAATTCGGTGACGTTCTCATCAAGTTGCGTCAGACTGCGCATAGACACAAGTATTTATCCCATATCGGTCGTAGCTTGAGCCAGCTCACTGTTCCAACCCCGTGTGAGTTTGGCGGTATATTAGAGAT
CTATGCTTCTCCTGTTCCCTCTCGCTATTGCGATTTGAGGTATTGGGACTACGGAGACTTATATAGGAGGGCTAGGTTAAGGTGGTTGGAGGCCCTTGATCTGGGGGAT**TAA**TTGAATTCCAGATTTGACGTAGTGTGGAAAGACACCAA
CTGCAAGGTCTACAGgaccctgcagggattct

>MW063112.1 Maize associated partiti-like virus isolate MAPLV.19P-149 RNA-dependent RNA polymerase (RdRp) gene, partial cds

>MF372918.1 Maize associated partiti-like virus isolate SS9A RdRp gene, partial cds

>MW063111.1 Maize associated partiti-like virus isolate MAPLV.19P-96-98 RNA-dependent RNA polymerase (RdRp) gene, partial cds

CLUSTAL multiple sequence alignment by MUSCLE (3.8)

```
CanPLV_309          -CCGTTTTACCAACGCGGGAAGCTTACACTTTTATACCT-----TGCGTTTAATG---TT
CanPLV_315          -CCGTTTTACCAACGCGGAAAGCTTACACTTTCATACCT-----TGCGTTTAATG---TT
MW063111.1          CCCGTTTTA-CAACGTGGAA----TTCATTTGTCCAGCTGGACCTACGCTTTAAGAATCG
MW063112.1          CCCGTTTTA-CAACGTGGAA----TTCATTTGTCCAGCTGGACCTACGCTTTAAGAATCG
MF372918.1          CCCGTTTTA-CAACGTGGAA----TTCATTTGTCCAGCTGGACCTACGCTTTAAGAATCG
                     ******** ***** ** *    * ** **    * **     * ** ** * *

NewPartiti_309      TTATACGTAAG---CAAGCCGGATGGCGTATCGCACTACTTCAAGTCACGGGACTTCAAC
NewPartiti_315      TTATACGTAAG---CAAGCCGGATGGCGTATCGCACTACTTCAAGTCACGGGACTTCAAC
MW063111.1          TTGTGCGTGGGTTTTAGACCGGATGGTCGACCACATTTGC---GGTGATGGGGCCTCAAC
MW063112.1          TTGTGCGTGGGTTATAGACCGGATGGTCGACCACATTAGC---GGTGATGGGGCCTCAAC
MF372918.1          TTGTGCGTGGGTTATAGACCGGATGGTCGACCACATTAGC---GGTGATGGGGCCTCAAC
                    ** * ***  *    *  ********   * * ** *       ** * *** * *****

NewPartiti_309      TTCATATTCTTCATTTCGACGGCCAAAAGCGATATCGCGATTACCCCGTAGTCCCGGGTT
NewPartiti_315      TTCATATTCTTCATTTCGACGGCCTAAAGCGATATCGCGATTACCCCGTAGTCCCGGGTT
MW063111.1          CTC-----CTCC-------CGCTCTCAGGTTATAATTCGGCTCCCTAGGAAGGAAGGGTT
MW063112.1          CTC-----CTCC-------CGCTCTCAGGTTATAATTCGGCTCCCTAGGAAGGAAGGGTT
MF372918.1          CTC-----CTCC-------CGCTCTCAGGTTATAATTCGGCTCCCTAGGAAGGAAGGGTT
                     **     ** *       **  *  * *  ***   **  * **  * *     *****

NewPartiti_309      CGCATTTGATCTGTCTCAACCTCGAGAAGATCCACTAGCGCGACGTGCAGTTGAGCATGT
NewPartiti_315      CGCATTTGATCTGTCTCAACCTCGAGAAGATCCACTAGCGCGACGTGCTGTTGAGCATGT
MW063111.1          TGCCTTTGATCTTGCCCCAAGTCGGGAAGATCCACTAGCGCGCCGTGCTGCAGAGCACGT
MW063112.1          TGCTTTTGATCTTGCCCCGAGTCGGGAAGATCCACTAGCGCGCCGTGCTGCAGAGCACGT
MF372918.1          TGCCTTTGATCTTGCCCCGAGTCGGGAAGATCCACTAGCGCGCCGTGCTGCAGAGCACGT
                     ** ********  * *    *** ***************** ***** *  ***** **

NewPartiti_309      CTTTGGGAAAGCCTATGTTGACTCGTGCTTCGCTCGTCTCCATCGAAGTACTATGTCCTA
NewPartiti_315      CTTTGGGAAAGCCTATGTTGACTCGTGCTTCGCTCGTCTCCATCGAAGTACTATGTCCTA
MW063111.1          GTTTGGGAAGTCGTACGTTGACGGGTGCTTTCAGAGGCTACACAGGAGCACCCTGTCCTA
MW063112.1          GTTTGGGAAGTCGTATGTTGACGGGTGCTTTCAGAGGCTACACAGAAGCACCCTGTCCTA
MF372918.1          GTTTGGGAAGTCGTATGTTGACGGGTGCTTTCAGAGGCTACATAGGAGCACCCTGTCCTA
                     ********  * ** ******  ******     * ** **  * ** **  *******

NewPartiti_309      TGAGGCGCTGGTGTCTGACTTATTCGAGTTTGATCGGTTGCTTCCTTGTCGCCCAGTCGA
NewPartiti_315      TGAGGCGCTGGTGTCTGACTTATTCGAGTTTGATCGGTTGCTTCCCAATCGCCCAGTCGA
MW063111.1          CGATGCGCTGGTGTCTGATATCATGGGCTACGAGCGCGTTCATCCGCGCCGCCGGCACAG
MW063112.1          CGATGCGCTGGTGTCTGACATCATGGGCTACGAGCGCGTTCATCCGCGCCGCCGGCACAG
MF372918.1          CGATGCGCTGGTGTCTGACATCATGGGCTACGAGCGCGTTCATCCGCGCCGCCGGCACAG
                     ** **************  *  * *  *  ** **  * * ***    ****    *

NewPartiti_309      TGATCCAGTCTATGCGATGGTTCTCGAGTCTGTGAGGCAGGACTTACACGCACCGAGATT
NewPartiti_315      TGATCCAGTCTATGCGATGGTTCTCGAGTCGGTGAGGCAGGACTTACACGCACCGAGATT
MW063111.1          TGATCCCGTGTACGCTATGGCTCTTGAGTCTGTGCGCCGGGATTTCTCGTCAACGGCGAA
MW063112.1          TGATCCCGTGTACGCTATGGCTCTCGAGTCTGTGCGCCGGGATTTCTCGTCAACGGCGAA
MF372918.1          TGATCCCGTGTACGCTATGGCTCTTGAGTCTGTGCGCCGGGATTTCTCGTCAACGGCGAA
                    ****** ** ** ** **** *** ***** *** * * *** **     ** **

NewPartiti_309      ACTCGTACCACATACTATCGGAGCGGTGACCAAACTTCCCGACTTTCCCCGGCAGAAGTC
NewPartiti_315      ACTCGTACCACATACTATCGGAGCGGTGACCAAACTTCCCGACTTTCCCCGGCAGAAGTC
MW063111.1          GCTCATCCCACACACCATGGGGGCTGTGCCGCATCTCGCTGACTTCCCTCGGCAGAAAAG
MW063112.1          GCTCATCCCACACACCATGGGGGCTGTGCCGCATCTCGCTGACTTCCCTCGGCAGAAAAG
MF372918.1          GCTCATCCCACACACCATGGGGGCCGTGCCGCATCTTGCTGACTTCCCTCGGCAGAAAAG
                     *** * ***** ** ** ** ** *** *  * **  * ***** ** ********

NewPartiti_309      TCCGGGTTTGCCGTATAAGAACATG---GGTTATAAGACCAAAGGTGAGGTTGTCGATGA
NewPartiti_315      TCCAGGTTTGCCGTATAAGAACATG---GGTTATAAGACCAAAGGTGAGGTTGTCGATGA
MW063111.1          CGCCGGCCTACCTTACAAGAACTGGCCCGGTTACCGATCCAAGGGTGATGTCGTAGATGA
MW063112.1          CGCCGGCCTACCTTACAAGAATTGGCCCGGTTACCGATCCAAGGGTGATGTCGTAGATGA
MF372918.1          CGCCGGCCTACCTTACAAGAATTGGCCCGGTTACCGATCCAAGGGTGATGTCGTAGATGA
                      * **  * ** ** *****   *   *****     **** ***** ** ** *****

NewPartiti_309      CACCTACCAGTTAGAGGAGATTAACAGGCGCTGGCACTACATTGGCTTCGGGCGTGATCG
NewPartiti_315      CACCTACCAGTTAGAGGAGATTAACAGGCGCTGGCACTACATTGGCTTCGGGAGTGATCG
MW063111.1          TCCTGATGAACTATGGAAGATCAATACTCTATGGCACCACATAAGCGCCCGGAAGGCCCA
MW063112.1          TCCTGATGAATTATGGAAGATCAATACTCTATGGCACCACATAAGCGCCCGGAAGGCCCA
MF372918.1          TCCTGATGAATTATGGAAGATCAATACTCTATGGCACCACATAAGCGCCCGGAAGGCCCA
                      *  *  *  **  * **** ** *  *  ****** ****  **  * **   *  *

NewPartiti_309      AGGGCCGTTACCGGATACCTGCCTATTTGCAAGATCTCAGGTATGTGACGTGTCCAAGCA
NewPartiti_315      AGGACCGTTACCGGATACCTGTCTATTCGCAAGATCTCAGGTATGTGACGTGTCCAAGCA
MW063111.1          CAAGCCCCTTGAAGATGTATGCTTATTTGCACGCTCACAGGTTTGTTCTGTTGAGAAGCA
MW063112.1          TAAGCCCCTTGAGGATGTATGTTTATTTGCACGCTCGCAGGTTTGTCCTGTTGAGAAGCA
MF372918.1          CAAGCCCCTTGAAGATGTATGTTTATTTGCACGCTCGCAGGTTTGTCCTGTTGAGAAGCA
                        **  *    ***   **  **** *** * ** ***** ***   **    *****

NewPartiti_309      AAAGGTTCGGGGCGTTTGGGGTTATCCCCTTTTCGTCTATCTTGAAGAAGCCCGATTCTT
```

```
NewPartiti_315   AAAGGTTCGGGGCGTTTGGGGCTATCCCCTTTTCGTCTATCTTGAAGAAGCCCGATTCTT
MW063111.1       AAAGATTCGTGGCGTGTGGGGGTACCCTATTGAGGTGTACCTAGAAGAAGCCAGATTCTT
MW063112.1       AAAGATTCGTGGCGTGTGGGGGTACCCTATTGAGGTGTACCTAGAAGAAGCCAGATTCTT
MF372918.1       AAAGATTCGTGGCGTGTGGGGGTACCCTATTGAGGTGTACCTAGAAGAAGCCAGATTCTT
                 **** **** ***** ***** ** **  **   ** ** ** ********* *******

NewPartiti_309   CTATCCGATACTGGAGCACCTAAAGAGTAAGGGTCATAACTTCCCAATTGGCTATGGTTG
NewPartiti_315   CTATCCGATACTGGAGCACCTAAAGAGTAAAGGTCATAACTTCCCAATTGGCTATGGTTG
MW063111.1       CTACCCTCTGATGGACTGGTTGAAGTCGCCGGAATGTAAATTTCCCCTATCTTATGGCCT
MW063112.1       CTACCCTCTGATGGACTGGTTAAAGTCGGCGGAATGTAAATTTCCTTTATCTTATGGCCT
MF372918.1       CTACCCTCTGATGGACTGGTTAAAGTCGGCGGAATGTAAATTTCCTCTATCTTATGGCCT
                 *** **  *  ****     * ***      *    *** ** **  *    *****

NewPartiti_309   TGAAATCAGTAAGGGTGGTCTTGTCGCCATATCCGAGATGGCGCTTCGTGTGCGCGGTGG
NewPartiti_315   TGAAATCAGTAAGGGTGGTCTTGTCGCCATATCCGAGATGGCGCTTCGTGTGCGCGGTGG
MW063111.1       TGAGATGGCAAATGGAGGGATGGTTGCGATAGCCGATCTTGCCCAACGGCTCGGCACCGA
MW063112.1       TGAGATGGCAAATGGAGGGATGGTTGCGATAGCCGACCTTGCCCAGCGGCTCGGCACCGA
MF372918.1       TGAGATGGCAAATGGAGGGATGGTTGCGATAGCCGATCTTGCCCAGCGGCTCGGCACCGA
                 *** **    ** ** **  * ** ** *** ****  * ** *  **  *  **   *

NewPartiti_309   ---CAAGTATATGATGACTGATTGGAGTAAATTTGACAAATCCATACCTCCTTGGCTGAT
NewPartiti_315   ---CAAGTATATGATGACTGATTGGAGTAAATTTGATAAATCTATACCTCCTTGGCTGAT
MW063111.1       CTCCAAGTACTTAATGATAGACTGGTCCAAGTTTGATAAGTCTATTCCGCCATGGCTAAT
MW063112.1       CTCCAAGTACTTAATGATAGACTGGTCTAAGTTTGACAAGTCTATTCCGCCATGGCTAAT
MF372918.1       CTCCAAGTATTTAATGATAGACTGGTCCAAGTTTGACAAGTCTATTCCGCCATGGCTAAT
                    ******  * ****  ** ***   ** ***** ** ** ** ** ** ***** **

NewPartiti_309   TCGGGATGCCTTTTCTCTTTTGGCTGAATTAATTGACTTCGACCACGTTGTTGATTCCGA
NewPartiti_315   TCGGGATGCCTTTTCTCTTTTGGCTGAATTAATTGACTTCGACCACGTTGTTGATTCCGA
MW063111.1       CCGCGACGCTTTTGATATAATAGCAGATGTCATAGATTTCGACCATGTCAGAGATTCTGA
MW063112.1       CCGCGACGCTTTTGATATAATAGCAGATGTCATAGACTTCGACCATGTCAGAGATTCTGA
MF372918.1       CCGCGACGCTTTTGATATAATAGCAGATGTCATAGACTTCGACCATGTCAGAGATTCTGA
                  ** ** ** ***  * *  * ** **  * ** ** ******** **    ***** **

NewPartiti_309   GGGTTTGCAGTGGCATGTCCGAGGATATCGTAGCAAGGCACGTTGGAAGAAGATTGTCGA
NewPartiti_315   GGGTTTGCAATGGCATGTCCGAGGATATCGTAGCAAGGCACGTTGGAAGAAGATTGTCGA
MW063111.1       AGGCAAGGTTTGGCCTGTCCGAGCCTATCGGAGTCGACGGCGTTGGAAGAAGTTAATTGA
MW063112.1       AGGCAAGGTTTGGCCTGTCCGAGCCTATCGGAGTCGACGACGTTGGAAGAAGTTAATTGA
MF372918.1       AGGCAAGGTTTGGCCTGTCCGAGCCTATCGGAGTCGACGACGTTGGAAGAAGTTGATTGA
                  **   *   **** ********  ***** **       ************ *  * **

NewPartiti_309   CTATTTTGTCGAGACTCCAGTCCGTACCTGTAAGGGCGAGCGTTTCCTTGTTAGATCTGG
NewPartiti_315   CTATTTTGTCGAGACTCCAGTCCGTACCTGTAAGGGTGAGCGTTTCCTTGTTAGATCTGG
MW063111.1       CTATTTTATTGAGACACCAGTCCGTACCTGCAAAGGTGAGCGCTTCTTGGTTCGAGGAGG
MW063112.1       CTATTTTATTGAGACACCAGTCCGTACCTGTAAAGGTGAGCGCTTCTTGGTTCGAGGAGG
MF372918.1       CTATTTTATTGAGACACCAGTCCGTACCTGTAAAGGTGAGCGCTTCTTGGTTCGAGGTGG
                 ******* * ***** ************** ** ** ***** *** * *** **   **

NewPartiti_309   TGTACCGTCGGGATCGTGCTTCACGAACGTTATTGATACCATCATTAACTGTATTGTTAC
NewPartiti_315   TGTGCCTTCGGGGTCGTGCTTCACGAACGTTATTGATACTATCATTAACTGTATTGTTAC
MW063111.1       TGTACCGTCTGGGTCTTGCTTCACGAACGTAATTGACTCAATCATTAATTGTGTTGTGAG
MW063112.1       TGTACCGTCTGGGTCTTGCTTCACGAACGTAATTGATTCAATCATTAATTGTGTTGTGAG
MF372918.1       TGTACCGTCTGGGTCTTGCTTCACGAACATAATTGACTCAATCATTAATTGTGTTGTGAG
                 *** ** ** ** ** ************ * *****  * ******** *** **** *

NewPartiti_309   CCGGTACATCTCTTATAATACTGTGGGTTCTTTTCCTCTTGGTGAAATGTACCTTGGGGA
NewPartiti_315   CCGGTACATCTCTTATAATACTGTGGGTTCTTTTCCTCTTGGTGAAATGTACCTTGGGGA
MW063111.1       CCGGTACATTTCCTATCAGACGACCGGAGCCTTCCCCCTCGGAGAACTCTACCTTGGGGA
MW063112.1       CCGGTACATTTCCTATCAGACGACCGGAGCCTTCCCCCTCGGAGAACTCTACCTTGGGGA
MF372918.1       CCGGTACATTTCCTATCAGACGACCGGAGCCTTCCCCCTCGGAGAACTCTACCTTGGGGA
                 ********* ** *** * **    **  * ** ** ** ** *** * ***********

NewPartiti_309   CGATGGTGTGTGTGTTTTCTCTTCGTCCAGTCTGGTTGATCTGAGTAGTATGGCCGCTCT
NewPartiti_315   CGATGGTGTGTGTGTTTTCTCTTCGTCCAGTCTGATTGATCTGCGTAGTATGGCCGCTCT
MW063111.1       TGATGGTGTGTGTTTCTTTCCCCGATCCTCCTATCTCGATTTGGACAATTTAGCGAAAGT
MW063112.1       TGATGGTGTGTGTTTCTTTCCCCGATCCTCCTATCTCGATTTGGATAATTTAGCGAAAGT
MF372918.1       TGATGGTGTGTGTTTCTTTCCCCGATCCTCCTATCTCGATTTGGACAATTTAGCGAAAGT
                  ************ * **  *    ***       * *** **   * * * **     *

NewPartiti_309   TGCTCACGAGCGCTTTGGCCTTGTTCTTAGTCCTGACAAATCATATGTTACTACCCAAGC
NewPartiti_315   TGCTCACGAGCGCTTTGGCCTTGTTCTTAGCCCCGATAAATCATATGTTACTACCCAAGC
MW063111.1       TGCCTTTGACTGCTTCGGATTAGTTCTTAACAGAGACAAATCATATGTGACATCTGTTGT
MW063112.1       TGCCTTTGACTGCTTCGGATTAGTTCTTAACAGAGACAAATCATACGTGACATCTGTCGT
MF372918.1       TGCCTTTGACTGCTTCGGGTTAGTTCTTAACAGAGACAAATCATACGTGACATCTGTCGT
                 ***    **  **** **  * *******     ** ******** ** **  *    *

NewPartiti_309   TGAGAACATCCACTTCCTTGGATACTACAATAGGCCCATCGGCATGCCTAACAGAGATCA
NewPartiti_315   TGAGAACATCCACTTCCTTGGTTACTACAATAGGCCCATCGGCATGCCTAACAGAGATCA
MW063111.1       TGAGAACATCCACTTCATTGGCTATCATAACGCACCACATTGTGCGCCCAATCGGGATCA
MW063112.1       TGAGAACATCCACTTCATTGGCTATCATAACGCACCACATTGTGCGCCCAATCGGGATCA
MF372918.1       TGAGAACATCCACTTCATTGGCTATCATAACGCACCACATTGTGCGCCCAATCGGGATCA
                 **************** **** **  * **    **     *   *** **  * *****

NewPartiti_309   GGCTCTTCTAATTGTATCGTTTGTTCATCCTGAGAAAAGTCGCGAGACTGCTGTCGAGTG
NewPartiti_315   GGCTCTTCTGATTGTATCGTTTGTTCATCCTGAGAAAAGTCGCGAGACTGCTGTCGAGTG
MW063111.1       GGATTTACTGGTGGTTTCCTTCATCCACCCCGAGCGATCACGAAGAACTGCAGAAGAGTG
MW063112.1       GGATTTACTGGTGGTTTCCTTCATCCACCCTGAGCGATCACGAAGAACTGCAGAGGAGTG
MF372918.1       GGATTTACTGGTGGTTTCCTTCATCCACCCCGAGCGATCACGAAGAACTGCAGAAGAGTG
                 ** * * **  * ** ** **  * ** ** ***  *   **    ***** *  *****

NewPartiti_309   CGCTGCTGCTGCGCTTGGCCAGTTGTGGGCAAATTTTGATCCTCTAATTGCTGAGTCATG
NewPartiti_315   CGCTGCTGCTGCGCTTGGCCAGTTGTGGGCAAATTTTGATCCTCTAATTGCTGAGTCATG
MW063111.1       TGCCGCTGCCGCGCTAGGGCAACTCTGGGCTAACTTTGACCCCAGAATAGCTGCGCTTTG
MW063112.1       TGCCGCTGCCGCGCTAGGGCAACTCTGGGCTAACTTTGACCCCAGAATAGCTGCGCTTTG
MF372918.1       TGCCGCTGCCGCGCTAGGGCAACTCTGGGCTAACTTTGACCCCAGAATAGCTGCGCTTTG
                  ** ***** ***** ** **  * ***** ** ***** **   *** **** *   **

NewPartiti_309   GTTTAGGGTCCTGTGCTATGTCTCAAACAGTTATGAGGTCGAATTCGGTGACGTTCTCAT
NewPartiti_315   GTTTAGGGTCCTGTGCTATGTCTCAAACAGTTACGAGGTCGAATTCGGTGACGTTCTCAT
MW063111.1       GTACCGCGTCGTGATCTTTGTGTCCGATGCATTCTGTGTTACACATGATGATATCGTGGT
MW063112.1       GTACCGCGTCGTGATCTTTGTGTCCGATGCATTCTGTGTTACACATGATGATATCGTGAT
MF372918.1       GTACCGCGTCGTGATCTTTGTGTCCGATGCATTCTGTGTTACACATGATGATATCGTGAT
                 **   * *** **  ** *** **  *    *     **   *   * ***  *  *  *

NewPartiti_309   CAAGTTGCGTCAGACTGCGCATAGACACAAGTATTTATCCCATATCGGTCGTAGCTTGAG
NewPartiti_315   CAAGTTGCGTCAGACTGCGCATAGACACAAGTATTTATCCCATATCGGTCGTAGCTTGAG
MW063111.1       TCAGCTCAGACGGACAGCCCACCGTCATAAATATCTTTCGCATCTTGGGCGCCATTT--T
MW063112.1       TCAGCTCAGACGGACAGCCCACCGTCATAAATATCTTTCGCATCTTGGGCGCCATTT--T
MF372918.1       TCAGCTCAGACGGACAGCCCACCGTCATAAATATCTTTCGCATCTTGGGCGCCATTT--T
                   ** *  * * *** ** **  * ** ** *** * ** *** * ** **    **

NewPartiti_309   CCAGCTC--ACTGTTCCAACCCCGTGTGAGTTTGGCGGTATATTAGAGATCTATGCTTCT
NewPartiti_315   CCAGCTC--ACTGTTCCAACCCCGTGTGAGTTTGGCGGTATTTTAGAAATCTATGCTTCT
MW063111.1       CCATCCCTGACGATACCCTCTCCCAATGAGTGGGGTCATCTGTTAGAGGTACTTCCTGCG
MW063112.1       CCATCTCTGACGATACCCTCTCCCAATGAGTGGGGTCACTTGTTAGAGGTACTTCCTGCG
MF372918.1       CCATCTCTGACGATACCCTCTCCTAATGAGTGGGGTCACTTGTTAGAGGTACTTCCTGCG
                 *** * *  **  * **  * **   *****  **     * *****  *   * ** *

NewPartiti_309   CCTGTTCC-------CTCTCGCTATTGCGATTTGAGGTATTGGGACTACGGAGACTTATA
NewPartiti_315   CCTGTTCC-------CTCTCGCTACTGCGATTTGAGGTACTGGGACTACGGAGACTTATA
MW063111.1       CCCGTTCCGAAGCGGCTTTTGCCAATTCGAC----GGTAT---GAGTATGGCCTCCTCTG
MW063112.1       CCCGTTCCGAAGCGGCTTTTGCCAATTCGAC----GGTAT---GAGTATGGCCTCCTCTG
MF372918.1       CCCGTTCCGAAGCGGCTTTTGCCAATTCGAC----GGTAT---GAGTATGGCCTCCTCTG
```

```
                    ** *****       ** * ** * * ***     ****    ** ** **   * * *

NewPartiti_309      TA-GGAGGGCTAG------------------GTTAAGGTGG--TTGGAGGCCCTTGATCT
NewPartiti_315      TA-GGAGGGCTAG------------------GTTAAGGTGG--TTGGAGGCCCTTGATCT
MW063111.1          CGCGGAAGGTTGGAAACGGCTCCACACTCCAGTTGAGGTGGATGTGGATAGTCCTGAGTA
MW063112.1          CGCGGAAGGTTGGAAAAGGCTCCACACTCCAGTTGAGGTGGATGTGGATAGCCCTGAGTA
MF372918.1          CGCGGAAGGTTGGAAAAGGCTCCACACTCCAGTTGAGGTGGATGTGGATAGCCCTGAGTA
                       *** ** * *                  *** ******   ****    * ***

NewPartiti_309      GGGGGATTAATTGAATTCCAGATT-------------------------TGACGTAGTGT
NewPartiti_315      GGGGGATTAATTGAATTCCAGATT-------------------------TGACGTAGTGT
MW063111.1          AGACTCTAAACTGCTCACCGCGTCCTTAGCTATGACGTTAAACTGACTATGACGTAGCGT
MW063112.1          AGGCTCTAAACTGCTCACCGCGTCCTTAGCTATGACGTTAAACTGACTATGACGTAGCGT
MF372918.1          AGGCTCTAAACTGCTCACCGCGTCCTTAGCTATGACGTTAAACTGACTATGACGTAGCGT
                     *    * ** **    **   *                          ******** **

NewPartiti_309      GGAAAGACACCAACTGCAAGGTCTACAGGACCCTGCAGGGATTCT
NewPartiti_315      GGAAAGACACCAACTGCAAGGTCTACAGGACCCTGCAGGGATTCT
MW063111.1          GGAAAGACACCAACAG---------------------------CT
MW063112.1          GGAAAGACACCAACAG---------------------------CT
MF372918.1          GGAAAGACACCAACAG---------------------------CT
                    ************** *                           **
```

>MW826382.1 Partitiviridae sp. isolate XZN141309 segment RNA1 genomic sequence

CLUSTAL multiple sequence alignment by MUSCLE (3.8)

```
MW826382.1          -----------------------TACATGCTTA---CCTACTTTCAA-GCTTTAT-----
NewPartiti_309      CCGTTTTACCAACGCGGGAAGCTTACACTTTTATACCTTGCGTTTAATGTTTTATACGTA
NewPartiti_315      CCGTTTTACCAACGCGGAAAGCTTACACTTTCATACCTTGCGTTTAATGTTTTATACGTA
                                           ****   * *   * * * ** ** * *****

MW826382.1          AGCTAGCCGGATGGCGAA-----------------ACGG-----------TATACTCCTC
NewPartiti_309      AGCAAGCCGGATGGCGTATCGCACTACTTCAAGTCACGGGACTTCAACTTCATATTCTTC
NewPartiti_315      AGCAAGCCGGATGGCGTATCGCACTACTTCAAGTCACGGGACTTCAACTTCATATTCTTC
                    *** ************ *                 ****            *** ** **

MW826382.1          CTTCTGCAAGAGCGAATGAAACCGTTGTTA-GAATCCCTCCTGCCAAGGGATTCGCATTT
NewPartiti_309      ATTTCG-----ACGGCCAAAAGCGATATCGCGATTACCCCGTAGTCCCGGGTTCGCATTT
NewPartiti_315      ATTTCG-----ACGGCCTAAAGCGATATCGCGATTACCCCGTAGTCCCGGGTTCGCATTT
                     **  *      **    *** ** * *   ** * ** * *      ** *********

MW826382.1          GATCTGTCCCCTTCACGGGAAGATCCCCTGGCGCGCCGTGCTGCCGAGCACGTCTTTGGA
NewPartiti_309      GATCTGTCTCAACCTCGAGAAGATCCACTAGCGCGACGTGCAGTTGAGCATGTCTTTGGG
NewPartiti_315      GATCTGTCTCAACCTCGAGAAGATCCACTAGCGCGACGTGCTGTTGAGCATGTCTTTGGG
                    ******** *   * ** ******** ** ***** ***** *  ***** ********

MW826382.1          AAGACATACGTAGACGACTGCTTTTCTCGCCTTCACCGCAGTCAGCTCTCGTATGATGCG
NewPartiti_309      AAAGCCTATGTTGACTCGTGCTTCGCTCGTCTCCATCGAAGTACTATGTCCTATGAGGCG
NewPartiti_315      AAAGCCTATGTTGACTCGTGCTTCGCTCGTCTCCATCGAAGTACTATGTCCTATGAGGCG
                    **  * ** ** ***   *****  **** ** ** ** ***    * ** ***** ***

MW826382.1          CTGGTTGATGATTTGATGGGGT---ATCAGAGGCAACATGCTCGCGCTTGTCGTGATGAT
NewPartiti_309      CTGGTGTCTGACTTATTCGAGTTTGATCGGTTGCTTCCTTGTCGC-CCAGTC--GATGAT
NewPartiti_315      CTGGTGTCTGACTTATTCGAGTTTGATCGGTTGCTTCCCAATCGC-CCAGTC--GATGAT
                    *****   *** **  * * **   *** *  **  *    **** *  ***  ******

MW826382.1          CCTGTGTATCAGATGGCGTACCAGTCGATCCGTCTAGACCTTCAGGCAAAGAGCAAGCTG
NewPartiti_309      CCAGTCTATGCGATGGTTCTCGAGTCTGTGAGGCAGGACTTACACGCACCGAGATTACTC
NewPartiti_315      CCAGTCTATGCGATGGTTCTCGAGTCGGTGAGGCAGGACTTACACGCACCGAGATTACTC
                    ** ** ***  *****    * ****  *  * *  *** * ** ***  ***    **

MW826382.1          ATACCTTACACTATCGGGGCCGTGCCTAAGCTAGCTGACTTTCCAAGAGCTAAGAGCCCC
NewPartiti_309      GTACCACATACTATCGGAGCGGTGACCAAACTTCCCGACTTTCCCCGGCAGAAGTCTCCG
NewPartiti_315      GTACCACATACTATCGGAGCGGTGACCAAACTTCCCGACTTTCCCCGGCAGAAGTCTCCA
                     ****  * ******** ** *** * ** **  * ******** *    ***   **

MW826382.1          GGACTACCCTGGAAAAACGAAGGTTTCCACACTAAGGGGGAGGTAGTGGATAGCGACGAG
NewPartiti_309      GGTTTGCCGTATAAGAACATGGGTTATAAGACCAAAGGTGAGGTTGTCGATGACACCTAC
NewPartiti_315      GGTTTGCCGTATAAGAACATGGGTTATAAGACCAAAGGTGAGGTTGTCGATGACACCTAC
                    **  * ** *  ** ***   ****   * ** ** ** ***** ** ***  *  * *

MW826382.1          AATGTCCGTGCTATCAACGTTCTTTGGCACCATATTGGTGCGGGTATCAAACATGACCCA
NewPartiti_309      CAGTTAGAGGAGATTAACAGGCGCTGGCACTACATTGGCTTCGGGCGTGATCGAGGGCCG
NewPartiti_315      CAGTTAGAGGAGATTAACAGGCGCTGGCACTACATTGGCTTCGGGAGTGATCGAGGACCG
                     *  *    *  ** ***   *  ****** * *****    **      * *  *  **

MW826382.1          CTGCCTGACACTTGTTTGTTCGCTCGATCTCAGGTTTGCTCCGTTGAGAAAGAGAAGATC
NewPartiti_309      TTACCGGATACCTGCCTATTTGCAAGATCTCAGGTATGTGACGTGTCCAAGCAAAAGGTT
NewPartiti_315      TTACCGGATACCTGTCTATTCGCAAGATCTCAGGTATGTGACGTGTCCAAGCAAAAGGTT
                     * ** ** ** **  * ** **  ********** **   ***    **  * *** *

MW826382.1          AGAGGTGTGTGGGGATACCCTCTCGCTGTGTATATGGAAGAGGCCCGGTTTTTCTATCCA
NewPartiti_309      CGGGGCGTTTGGGGTTATCCCCTTTTCGTCTATCTTGAAGAAGCCCGATTCTTCTATCCG
NewPartiti_315      CGGGGCGTTTGGGGCTATCCCCTTTTCGTCTATCTTGAAGAAGCCCGATTCTTCTATCCG
                     * ** ** ***** ** ** **    ** *** * ***** ***** ** ********

MW826382.1          ATTATGGATCATCTAAAAGGGTCTGGGCATAAGTTTCCGATCGGCTATGGCTTTGAGATC
NewPartiti_309      ATACTGGAGCACCTAAAGAGTAAGGGTCATAACTTCCCAATTGGCTATGGTTGTGAAATC
NewPartiti_315      ATACTGGAGCACCTAAAGAGTAAAGGTCATAACTTCCCAATTGGCTATGGTTGTGAAATC
                    **  **** ** *****  *    ** ***** ** ** ** ******** * *** ***

MW826382.1          GGTAAAGGTGGAATGGTTGGTTTATCTGACATGGTTCTGAGGCATCCAGAATCTAAGTGT
NewPartiti_309      AGTAAGGGTGGTCTTGTCGCCATATCCGAGATGGCGCTTCGTGTGCGCGGTGGCAAGTAT
NewPartiti_315      AGTAAGGGTGGTCTTGTCGCCATATCCGAGATGGCGCTTCGTGTGCGCGGTGGCAAGTAT
                     **** *****  * ** *   **** ** ****  **  *    *  *     **** *

MW826382.1          CTTATGACTGACTGGTCTCAATTTGACAAATCTGTCCCCGCCTGGCTGATTCGTGATGCT
NewPartiti_309      ATGATGACTGATTGGAGTAAATTTGACAAATCCATACCTCCTTGGCTGATTCGGGATGCC
NewPartiti_315      ATGATGACTGATTGGAGTAAATTTGATAAATCTATACCTCCTTGGCTGATTCGGGATGCC
                     * ******** ***  * ******* *****  * **  * *********** *****

MW826382.1          TTTTCCCTCTTGGCTGAGTTGATCGATTTCGACCATGTGTTAGATTCTGAAGGCTTGGTC
NewPartiti_309      TTTTCTCTTTTGGCTGAATTAATTGACTTCGACCACGTTGTTGATTCCGAGGGTTTGCAG
NewPartiti_315      TTTTCTCTTTTGGCTGAATTAATTGACTTCGACCACGTTGTTGATTCCGAGGGTTTGCAA
                    ***** ** ******** ** ** ** ******** **  * ***** ** ** ***

MW826382.1          TGGCCAGTCCGAGCTGTTAGGAGTCGACGCA-GATGGAAGAAGCTCGTTGATTACTTCGT
NewPartiti_309      TGGCATGTCCGAGGATATCGTAG-CAAGGCACGTTGGAAGAAGATTGTCGACTATTTTGT
NewPartiti_315      TGGCATGTCCGAGGATATCGTAG-CAAGGCACGTTGGAAGAAGATTGTCGACTATTTTGT
                    ****  *******    * * ** * * *** * ********* * ** ** ** ** **

MW826382.1          CGAGACACCTGTACGCACTTGCAAAGGCGATCGCTTTCTCGTTCGCGCTGGAGTGCCTTC
NewPartiti_309      CGAGACTCCAGTCCGTACCTGTAAGGGCGAGCGTTTCCTTGTTAGATCTGGTGTACCGTC
NewPartiti_315      CGAGACTCCAGTCCGTACCTGTAAGGGTGAGCGTTTCCTTGTTAGATCTGGTGTGCCTTC
                    ****** ** ** ** ** ** ** ** ** ** ** ** *** *  **** ** ** **

MW826382.1          TGGCTCGTGTTTCACCAATATCATTGATACTATTATTAACTGCTTGGTGTCACGATATCT
NewPartiti_309      GGGATCGTGCTTCACGAACGTTATTGATACCATCATTAACTGTATTGTTACCCGGTACAT
NewPartiti_315      GGGGTCGTGCTTCACGAACGTTATTGATACTATCATTAACTGTATTGTTACCCGGTACAT
                     ** ***** ***** **  * ******** ** ********  * **  * ** **  *
```

```
MW826382.1          ATGTTACCAAACTATCGGCCGTTTTCCCCTTGGTGAGATGTACCTGGGCGATGATGGGGT
NewPartiti_309      CTCTTATAATACTGTGGGTTCTTTTCCTCTTGGTGAAATGTACCTTGGGGACGATGGTGT
NewPartiti_315      CTCTTATAATACTGTGGGTTCTTTTCCTCTTGGTGAAATGTACCTTGGGGACGATGGTGT
                     * ***  * *** * **   ****** ******** ******** ** ** ***** **

MW826382.1          AGTCGTTCTTCCGTCTGACTCTGTGGTTAATCTAGACGCCATGGCTGACCTTGCGCTCTT
NewPartiti_309      GTGTGTTTTCTCTTCGTCCAGTCTGGTTGATCTGAGTAGTATGGCCGCTCTTGCTCACGA
NewPartiti_315      GTGTGTTTTCTCTTCGTCCAGTCTGATTGATCTGCGTAGTATGGCCGCTCTTGCTCACGA
                        *** *  * **   *  * ** ** ****       ***** *  ***** * *

MW826382.1          GAAGTTTGGATTTTCTCTCTCACGTGACAAATCTTTCGTTACGACGAACGCTGAGAATAT
NewPartiti_309      GCGCTTTGGCCTTGTTCTTAGTCCTGACAAATCATATGTTACTACCCAAGCTGAGAACAT
NewPartiti_315      GCGCTTTGGCCTTGTTCTTAGCCCCGATAAATCATATGTTACTACCCAAGCTGAGAACAT
                    *   *****  **  ***    *  ** ***** *  ***** **  * ******** **

MW826382.1          CCACTTCTTGGGCTATCATAACCGACCACTTGGTATGCCAAACAGAGATCAGTCCCTTCT
NewPartiti_309      CCACTTCCTTGGATACTACAATAGGCCCATCGGCATGCCTAACAGAGATCAGGCTCTTCT
NewPartiti_315      CCACTTCCTTGGTTACTACAATAGGCCCATCGGCATGCCTAACAGAGATCAGGCTCTTCT
                    ******* * ** **  * **  * **  * ** ***** ************ * *****

MW826382.1          TATTGTTTCTTTTGTCCATCCTGAGCGCACAAGGAGAACCGCTGAAGAATGCGCAGCCGC
NewPartiti_309      AATTGTATCGTTTGTTCATCCTGAGAAAAGTCGCGAGACTGCTGTCGAGTGCGCTGCTGC
NewPartiti_315      GATTGTATCGTTTGTTCATCCTGAGAAAAGTCGCGAGACTGCTGTCGAGTGCGCTGCTGC
                     ***** ** ***** *********   *   *    ** ****  ** ***** ** **

MW826382.1          CGCTTTAGGCCAATTATGGGCTAACTTTGATCCCAGGCTTGCGACTATGTGGTACCGAGT
NewPartiti_309      TGCGCTTGGCCAGTTGTGGGCAAATTTTGATCCTCTAATTGCTGAGTCATGGTTTAGGGT
NewPartiti_315      TGCGCTTGGCCAGTTGTGGGCAAATTTTGATCCTCTAATTGCTGAGTCATGGTTTAGGGT
                     **  * ***** ** ***** ** ********     ****        ****   * **

MW826382.1          CTTAGTCTTTGTTGCAGACGCCTTTTTGGTAAGCTTTGACGAGGTACTTGTGCAGCTCAA
NewPartiti_309      CCTGTGCTATGTCTCAAACAGTTATGAGGTCGAATTCGGTGACGTTCTCATCAAGTTGCG
NewPartiti_315      CCTGTGCTATGTCTCAAACAGTTACGAGGTCGAATTCGGTGACGTTCTCATCAAGTTGCG
                    * *   ** ***  ** **   *    ***    ** *  ** ** **  *  ** *

MW826382.1          GCGAACCGCCCATCGCCATAAATATTTGTCACATATTGGGCGAAACTTCAACACTCTTAC
NewPartiti_309      TCAGACTGCGCATAGACACAAGTATTTATCCCATATCGGTCGTAGCTTGAGCCAGCTCAC
NewPartiti_315      TCAGACTGCGCATAGACACAAGTATTTATCCCATATCGGTCGTAGCTTGAGCCAGCTCAC
                     *  ** ** *** * ** ** ***** ** ***** ** ** * *** * *   ** **

MW826382.1          TGTTCCTCGTCCAGACGAGTTTGATAATATCCTCGACGTGCTTCCGTCTCCAGTTCCCTC
NewPartiti_309      TGTTCCAACCCCGTGTGAGTTTGGCGGTATATTAGAGATCTATGCTTCTCCTGTTCCCTC
NewPartiti_315      TGTTCCAACCCCGTGTGAGTTTGGCGGTATTTTAGAAATCTATGCTTCTCCTGTTCCCTC
                    ******    **    *******    ***  * **  *   *  * ***** ********

MW826382.1          CCGTCTACCACCTTCTTCTCGACGGTAT---GACTATGGTGAGCTCGCGCGTGAAGCATG
NewPartiti_309      TCG-CTATTGCGATTT-----GAGGTATTGGGACTACGGAGA-----CTTATATAGGAGG
NewPartiti_315      TCG-CTACTGCGATTT-----GAGGTACTGGGACTACGGAGA-----CTTATATAGGAGG
                     ** ***   *  * *       ****    ***** ** **     *   *  ** * *

MW826382.1          GCTATTTTGGTCGAGCGTTTCAGAAGATGATGTTGACAATCCAGGCTAGTTACCTGGGAT
NewPartiti_309      GCTAGGTT---------------AAGGTG--GTTGG------AGGCCCTTGATCTGGGGG
NewPartiti_315      GCTAGGTT---------------AAGGTG--GTTGG------AGGCCCTTGATCTGGGGG
                    ****  **               *** ** ****       ****   * * *****

MW826382.1          GATGGTAGACC-----ATCTGACGTAGTGTGGAAAGACACCAACTGGTAAGCCA------
NewPartiti_309      ATTAATTGAATTCCAGATTTGACGTAGTGTGGAAAGACACCAACTGCAAGGTCTACAGGA
NewPartiti_315      ATTAATTGAATTCCAGATTTGACGTAGTGTGGAAAGACACCAACTGCAAGGTCTACAGGA
                      *  * **       ** ***************************  * * *

MW826382.1          ---------------
NewPartiti_309      CCCTGCAGGGATTCT
NewPartiti_315      CCCTGCAGGGATTCT
```

(C) Grapevine line pattern virus (GLPV)

Host: ***Cannabis sativa***

>GLPV_RNA1_alyu302
GTTTGATGCAACTCTTTTGGTTCCAATTCCAATTGATTGTATTGCTCTTACGTACCATTTCTCAGCGACGAG**ATG**GCTTTTAGTTTTAATGTTCGTGACCTTGTTGACGCCGAGGGGAGTCAGGCTATCGGTGTCAAAGCTCTTGTAGAT
AATTTAGCAACGCAGATGGCTAGTGATCAAATAGAATATAGTAAGCGCGCGAAGCAAGTGTTTATACGTCAGCAGTTAAGCGTGCAAGAAGGGGAAAAAATTCGGAAACGTTTTGGAGGAGCTTATGAATTGAAGTTGACACAGGAATTT
ACAGCACCCCATTCGTTTGCTGCCGCGCTTAGACAATGTGAAACATTGGAATGTTTGGATTTCTTTCCCGAAGGAGAGATTCTTGATTTCGGTGGGTCCTGGTTGTTTCATTGGAAGAGGGGCTCTCAAGTCCATAGTTGTTGCCCCATT
TTAGACGCTAGGGACGCAGCCAGACATCAGGAACGTTTGGTTCATATGGAGAAAATGGTTCGTAGGTCAAGTTCGAAGTATTGCGAACTACCTGGACCGAATTTCTGTTGTCTGAAGGCTGAAGATTGCGAAGTGCAATGTCCTTATGCT
ATTTCGATTCATGGTGGGTATGATATGGGCTTTCAGACATTATGTTCCGCTATGCATAAACATGGTTGTGTTATGTTGCGCGGTACCATGATGTTTGATTCGGAGATGCTTCTTTATGAATCTGGGTTTATCTCAGATTTGAATTGTGTC
TGGTCTAAGCGTAACGGCATAGTGGCTTTTGATTTTCGAGATGAATCTACGTTGTCTTATACCCACTCTTTTAAGAACGTTAAATCTTTCCTTACTGACCAAAGTCATGTCATTGGTACTACCGCATACATTTTGGAGCGATGTACCATT
GAATTTGGGATAATGTCTTATAAAATTACCGCTGTTTCTGGTGATATACCGAGGACAAGGATAAGACACTGTATCTGGTTCCCGCGAACTAGAGATTATCTACATATTAATGTCGTGGATAATTCTAAAGTGTCTCACCGCCTAGCTTGG
CGGAAAGTCAAGGTTAAACTAGACACGGTAAGAGAAGTTGAGGAAATTGCTTTCCGATGCTTCAAAGACCAAAAGCCCTGGGAGGAGAATTTACGTTTAATTGCTTCCACCTTATCTGCAAAGTCATCAACGATTATCGTTAATGGTATG
AGTATGATGGCTGGTGAACGATTAGATCCTACAGATTATCACTATGTAGCCTTTTCTTTGCTACTTAATTGTAAGCGAAAGTTTGAAGAGTTACAATGTGTATATAAGAAAATCGTTTGGCAGGGATGGTGGAATCATTGTATGAAATGG
TGGTCTGATTCGACTAGATTTCAAGATTTTTTAGCCGCTTGTTTCCCTTCTTTATTAGATTATTATCATTCTAAAGTTTTTGTAGAAAAAATTTGTGAGTGTCAGGTATTTGAGTCTGAGCTTGAGGCGTTGTCCGGTTCCAAGTCTTGG
GAGGATGATGCCTATTCTGTGTTAAATAATAATTTACTTTTGTGTGCTTTAGCTTTAAGTAAGACAAAAGAAAAAGAAAAAGAAAAAGAAATTCAGGATGAAAAGCCTGATGTAGAGGGAGAAAAACCTGTGCATTCACAACCCAACACT
CATGTGGTTGGTGATGTGCCTCCTAAAGTCGTCATTAAGGATATTGATTCTCGACCGGGTAGAATGGAGTTGAGGGCCCAAGCAAAGGACGAGTTCGTGGCCTACACACGCAAGTTACATTCGAATGCTGCGGCTAATTTAGCACGCCTT
TGGAAATTGTGTGGTGGGAGTGGATCAGATAACTTTGTTTCCACGAACTGTCTTAACGTTTTGAAGTTGGGGGATTCATTAATTAACTTTCATAAACGTTCTGGTTGGATTTTTGAGACCAATGAAACTTATCAGATAGGATTCAATGGA
AATGGTCTCGGTGTTAAATCACCTGACGAGAATTACATTGTTGATAAGACATGTATTCTCGATAATCTAGCCAGTATTGCTGAAGGTGCTGATAAAGCGATGAATTCCTCTGCTACATTCGTGCTTATGGACGGTGTCGCTGGTTGCGGT
AAGACTACTAGCATTTGTGACCAATTTAATGCTGAAACTGATCTAATAGTCACCGCCAACCGTAAGTCTTCGGAAGAGATTCGTGACAAACTTTTTAAGGATAGCCCTGATTTAGGTAAGCGGTTTGTTCGTACTGCAGATTCTGTGCTT
ATGCACGATTGTATAACCGCTCCTCGTATTCTCTTTGATGAATGCGGGCTATTACATTTTGGACAATTGGTTGCTGTGGCAGGGAAAGTGCAAGCAAAGGTTGTTCTTGGTTTTGGTGATTCTGAACAAATTTCCTTTATAAACCGAGAT
AAAACTTTTCAAATGAAATATTCACAGCTGTCCGTCGATGAGTCTACTGTCGTGAAGCATACTTACCGATGCCCGAAAGATGTCGTGCATCTCGTAAAAACAGTAATTAAGGGGAAGGTGCGACACTCAAAATATCATGGTTGGGAATCT
AAGTCCAATGTGGTGCGGTCTGTAAGTGCGCCTATTGATGTCGTCGGGTTACCAGGAGTGAAACTGGAGCCTGATTGGGTGTATCTTACAATGACACAGTATGATAAGGCAAGCCTGCTTAGCAAAGCAAAGGAATTACATTTAAATATG
TCTGATAAGATAAAAACCGTCCATGAAGCCCAAGGAATTTCAGTTCCAAAGGTAAGATTGGTACGGTTAAAGAATACAAAATGTGATCTCTTTTCTGCTGAAGCTCACTGTTTAGTTGCGCTTACTCGACATACTCGTAGATTTGAATAT
CTTAGAGTTCCTGGGTTAAAGGGTGACTTAATTGAAGAGGCTTGTAAAGCATCATTAAGCCTTCAAATT**TAA**TTTTGAAGGTAATATAAGTTGAATGAGTACTATAACTCATAGTCGTTGTTGTAACGCAGTTGATTGGGTACTATAACC
CAAAGTCGTGGTTGACACGCCTTTTGCTTATATAAGAGATATAAGTAATTGAGTATAGGAATCCGTTCTTCCCGTCGGATGCATCTGTTCGAGTCTCATGAGGCTCATTAGGAACAGCTATACGCTGATGCAGCAATGCTGCATCGGTCT
CTAGGGGAGACCA

>GLPV_RNA2_alyu302
GTTTGATGCAACTCTTTTGGTTCCAATTCCAATTGATTGTATTGCTCTTACACACCGTTTCTCAACGCCGAA**ATG**GATTTGTTTTCAATGTTTAATTCTGGTCTTCTTACCGCTGATTGTGAGTCTTTAATTAGATATACTAAGTTAGGT
TTCGAGGCAGATACTTTTGATCAGGCATCTTGGGATATTAATCTTGAGATGAGTGAGGTCCTTTCGGAACAGCTAAACGTTTCGGTCGATAGACTTTTAGTTGCTCTGTTTAATGGACTGATCTAGCTGATCTTGAGCCTGATCTCGAA
GTGTGTCATGAAGGTTTAGGTTTTGAGTCTGCACATATTGTCGATTGTGTTTCGATGACTGATTATATGGACTCATTTCAGATTGAGCCTTTAGTTTCAGTTGTCGTGCCTGATCCTGTAGATACTTTAGTAGAGGACCTAGAGTCGGAC
TTCCCGTTGGTGCATTCTGAGCCGGAAGTACGATTGATGGAGACTAGCTATGGGAATCACTTCACGCTTGTACAGGAAGAGTTTATTGAAGAGTGTCCTGCTGCAGCTATTAATATTGCCACTTGCGAACCAGTCATGCAGATCACTGAG
TGTGATCTTGAATTACCAATGCCTATCTTGACTGGTCGCAGTGATCGGGTTTCACTCGAAAAGTTGCAATGCACTGTGGATGCTATTCTACCAACTCATGCCCGATTCGATGACACTTTTCATCAAGCTTTCGTGGAAAATGGAGATATT
AGTCTCGATTTTGACAGAATTCGGTTGAAACAATACGCCGGTGATTGGTATAAGGATCCTGATGGGTTTTACCAACCCAATCTCCTTTCTGGAGGTTGTGCTAGGCGTATTGGTTCTCAAAAAGAGGCTTTAATTGCTATACGTAAGCGT
AATGCTGATGTACCTGAATTAGCCGGTTCTGTTAATATTGATGAAGTTGCTAAATGGGCAGCTGATAATTTCATGAAAGCTTTTGTTGTTAATGTCAATCCTTTAGTACAAGTGATGACACGCATGCAGGCCTATATGTCTAAATGGGCG
GATAGGGTCGATCCTCTCTTTGTTCTGAGTGAAACAAATTTACAACGCTATCAACATATGATAAAGACTGATGTTAAGCCTGTTGTAGCGGATGGAATGAACTTAGAGAGAGCCGTACCAGCCACCATCACTTTCCATGATAAGATGGTA
TGCTCTAATTTTTCGCCTTGGTTTACTGCACTTTTCGACGGATTTCAGAAGTCTTTAAACTCACGTGTTCGGATTCCTTCCGGACCAATCTCTACTCTTGAGATGAGATATGGGTTTAAAAATAAGTATTATGTTGAAATAGATCTGTCG
AAATTTGATAAGTCTCAAGGTGAATTACATTTAGAGTTTCAACGGCTGATCCTTCTGCGTCTTGGGTTACCAGCACACCTTGTGAATTGGTGGTGTGATTTACATATAAGGTCTTTCATGAGTGATCCTAATGCTGGTGTGGCGTTCCAA
TGCGCATACCAAAGAAGAACTGGTGATGCTTTCACATATTTTGGCAACACACTGGTAACAATGGCTGAATTTGCTTGTTGCTTTGATTGCTCACAGTTTGAGTGTATGCTGTTTTCCGGAGATGACTCTTTAGCTGTCTCTAACAATCCA
ATTACCGGAGATACCGATCTGTTCGCCAGTCTTTTTAATATGGAGAGTAAGGTTATGGCTAATCCAGTACCATATATTTGCTCAAAATTTTTAATAGAGGACAGTTTTGGTAACTCTTTCTCAGTTCCTGATCCCATTCGTGAGTTTCAA
CGTTTGGGAAAAAAGAAAATTCAAGTTCAAAAGAATCATGATGCACTCTTTGAACAATATCAGGGATTTCGAGATCGTATGAAATATCTTAGACACTTGGATGATGGAATGATTGATCAATTAAAGGTGTACTTTGATATGAAATACAAT
AAAGGTAGAGATTGTATTGATGACTTTTTGGGTGGTTGTATTTATTATGCTGATAATTTCAAGCATTTTTGCGAATTATACGACACCCCTCGTGCTACTGAAGTCAGAGCCTTGGTCGCTTTAGTTGATAAGGGCTCATTGCGCCTCCCG
TCTCGTATT**TAA**ATTATGCCTTTCCTTTCTGTTGATGTCGTTTGTCGATTCTGGGAAGCTGGGTTTACTCTTGCTACTTCCGATCAAATTTGGTTGTTAGAAAGTTTGAAACCACTTCCAAAGGAATTAAGGGTCACGATTCTTGAGAAA
TATCTCATGAATCAGGTGTTAGTTGAATGAGTACTATAACTCATAGTCGTTGCTGAAACGCAGTTGATTGGGTACTATAACCCAAAGTCGTGGTTGACACGCCTTTTGCTTATATAAGAGATTTAAGTAATTGAGTATAGGAATCCGTTC
TTCCCGTCGGATGCATCTGTTCGAGTCTCATGAGGCTCATTAGGAACAGCTATACGCTGATGCAGCAATGCTGCATCGGTCTCTAGGGGAGACCA

>GLPV_RNA3_alyu302
GTAGTCTTTTCTTTTGCTTTAAGTTTGCATAGCATAAGTTGAGTCAATATTTGCCTGCAATAACTGGTTTTCAAGTCTTCGAATCTTAGTTGATTTGCTTGATTTCCGGTGTGTGTGAGCCCAAGTGGAATTCCAGTTCTGCTTCCCTCA
CTGTTTGTCAGGTATTCGAGGCCTTTTTTAAGGCTAGCTGCTCCGATCCATTGATCTTGTTGAAAACCGCGCTGGGTGTAGGTCCCCGTTTGTCAAAGGACTAGCATATGCGCAGCTTTTTATATAAGAATTGAGGTTGACATCTCGAGG
TCTTAGCCTTCGAGCTCTCTTTGGATTGTTGTGACTTACCTTTTTTAAGGTCTCGTCTACAAGCAAATTTGTAAATAGACT**ATG**TCGATTCTAACTCGTTCCTTCAGCTCTCGCTCCCTGACTAAGCTTGATGGTACTATTAATGAGTAT
CTCGGCTCGAAAGAGTTCGAGGATGATATGCGTCAACAAGCTGGTTATGGCCGTTGGAAGGGTCTTAAGGTTGGTGGTGGTGAACGCTCATTTGTTTTGGTTCCAGAGAACTCGTACAATAAGCTCACTGGTCTGTTTCGTGCAGATTAT
GATAAAGGGTTGGTACCGAGTAAAGGATACATGCACCTTAAATGGGTCATGGTTTTCTTGGTCTCGCATGTACCTAAGTCGAGTGGAGGCTCTGTTACCTTATCATTAAAGGATCCTGGATGGTCCCTCACCGATCCACTACCTGATACC
AGTTTTGAAATGGCTTTAAGCTCACTTCCGAGAGTCTGTCTTTTAACGACAGATTACGATCTTCCATTGTCGAGGAAGCCTATCAAACTGGGAGACAGAGGCGAGATGCGAAGGATGTTCTTATTGTCAGCGAAGGTGACTGGCTTAGTT
GGAGCAGGAGATGCGTTATCGCTTTTTCCCATTTGGGATTGCGATTTTCGTTCTTCTTGCAACAACTATGAGGTTGTTCCGTGCACTTCTGTGCCTATTACCAGATGCATCCGTTCGGATGTTCTGAGTTGCGTTGCCGAATTAAATCGG
TACGTAAAAGGAACTCTCTTATCCTTACCCCGGAGTTCGGTCTCTGGGGCAAGGTTTGCCGCGCCTGAGCTGATAAATCAAAACGAGTCTGAGTCTGATTCTCGCACTGCTGTGGTGCCTGAGTCTGCGGGTGATCGTTCTGCCCCTCCG
CAGGGATTAGCGCAGAGCCCCGCTTTGTTTAATGGTGTCGAGGATGTGTCTAAGGAAGTAATTCCGAAGATACCTTAATCCTTTCTGTCCATGCATTTAGTTTAGTTGCATGTAGGTCGGGTAGGAGGAAAGTGATAGATACAGTTTTGA
GTTCTGCAGCCATTCAAGGGGTTGGTTCTCAAGAGGTTATCCTCTTAAAGAAGATTGTTGAAAATGAGTAGGTTGATGTTTGCGATGGAGATAGATTCATGTCTATCCCAGTTCGTGTTCAACTCTCAGTTACTTGATTTCTACCCTTGC

```
ATTTAGATAGTTTGGCATTGTATATTCTTATCTTAGCATGAGTCAAAATCCTAATCGACGAAGAAATAGGGCAAACCAGAATAGATCGCGCCGGCAACGGCAAGCTCAAGACGCTGCTGCGTTTCGTGCGCTTTCGTCTTCTTCTAGATT
GGGTCCGGTCACATCCGGTATGGTTGATGCGGGTGGTATCCCGGTTACTCCAGGTTTTGTGGTCACTTCGGTGACATTGAACGAGCCTTTTCAGTGGGCTGGTGCATGCACAGGTGCCTGGACTCTCTTCCGAGAGCGTAGCTTGGTGAT
TCCTCCTGCTCTTCCTCCCGAGACTCGCCTGAATAATGTGATCTTAAGAGTGACACCCACCCCTGGGTTAAGTTCTTTTGATATTTGGGCGGCGATTGCTGCGGCTAAGGGCTCTCTTCCGACTGCGGCGGATTTTGATGCTCTTAATGT
GCCGTTTTTGACAATGAACGGTACGTCTCGTATTCGGCATGTGGTTATACCATTTGCTAATCGTACGGTGGCTGACCTCTCGCAGCAACGTGTCTGGCTTGGTGCCAGAACTGGGACAGTGACTGCTGATAACTTAGTTGTTGGCTTGGT
GAGGGTGTTTATTGAACACCGCCCAATACCCCAAGTACAAGTTGTCCCGGTATGAGGTACATGGTTTTAACAGTTTCCTTAAACTGTAAGCGTCGTTGCGACGTGATACCTGATACGGATGAGTGTGGGAATCTCATTCGAGTGAAGCAC
TACTCCCTCCGTGCCGAAGCGGGAGTATGTGGTGTGGTTCAGGTGTGGGTGTTCAAGTTTTAACAGTTTCTTTAAACTGTAGTCGTTGCTGAAACGCAGTTGATCGAGTACTATAACTCGAAGTCGTGGTTGACACGCCAGTTACTTATA
CAAGAGGTATAAGTGATTGAGTATAGGAATCCATTCTCCTCGTTGGATGCATCTGTCCGAATCTCATGAGATTCATTAGGGACAGCTATACGCTGATGCAGCAATGCTGCATCGGTCTCTAGGGGAGACCA
```

Alignment of 5’ and 3’-termini of genomic RNAs

5’-terminus

```
GLPV_RNA3_alyu302      GT---AGTCTTTTCTTTTGCTTTAAGTTTGCATAGCATAAGTTG-----AGTCAATATTT
GLPV_RNA1_alyu302      GTTTGATGCAACTCTTTTGGTTCCAATTCCAATTGATTGTATTGCTCTTACGTACCATTT
GLPV_RNA2_alyu302      GTTTGATGCAACTCTTTTGGTTCCAATTCCAATTGATTGTATTGCTCTTACACACCGTTT
                       **   *  *   ******* **  * **   ** *  *   ***     *   *   ***

GLPV_RNA3_alyu302      GCCTGCAATAA--------CTGGTTTTCAAGTCTTCG----------------------
GLPV_RNA1_alyu302      CTCAGCGACGAGATGGCTTTTAGTTTTAATGTTCGTGACCTTGTTGACGCCGAGGGGAGT
GLPV_RNA2_alyu302      CTCAACGCCGAAATGGAT-TTGTTTTCAATGTTT-------------------------
                         *  *    *         *  ***  * **
```

3’-terminus with a conserved tRNA-like structure ending with the universal CCA required for aminoacylation

```
GLPV_RNA3_alyu302      TTCAGG------------TGTGGGTGTTCAAGTTTTAACAGTTTCTTTAAACTGTAGTCG
GLPV_RNA1_alyu302      -CCTTCAAATTTAATTT-TGAAGGTAATATAAGTTGAATGAGTACTATAACTCATAGTCG
GLPV_RNA2_alyu302      TTCTTGAGAAATATCTCATGAATCAGGTGTTAGTTGAATGAGTACTATAACTCATAGTCG
                         *               **       *     ** **    * ** ***    ******

GLPV_RNA3_alyu302      TTGCTGAAACGCAGTTGATCGAGTACTATAACTCGAAGTCGTGGTTGACACGCCAGTTAC
GLPV_RNA1_alyu302      TTGTTGTAACGCAGTTGATTGGGTACTATAACCCAAAGTCGTGGTTGACACGCCTTTTGC
GLPV_RNA2_alyu302      TTGCTGAAACGCAGTTGATTGGGTACTATAACCCAAAGTCGTGGTTGACACGCCTTTTGC
                       *** ** ************ * ********** * *******************  ** *

GLPV_RNA3_alyu302      TTATACAAGAGGTATAAGTGATTGAGTATAGGAATCCATTCTCCTCGTTGGATGCATCTG
GLPV_RNA1_alyu302      TTATATAAGAGATATAAGTAATTGAGTATAGGAATCCGTTCTTCCCGTCGGATGCATCTG
GLPV_RNA2_alyu302      TTATATAAGAGATTTAAGTAATTGAGTATAGGAATCCGTTCTTCCCGTCGGATGCATCTG
                       ***** ***** * ***** ***************** **** * *** ***********

GLPV_RNA3_alyu302      TCCGAATCTCATGAGATTCATTAGGGACAGCTATACGCTGATGCAGCAATGCTGCATCGG
GLPV_RNA1_alyu302      TTCGAGTCTCATGAGGCTCATTAGGAACAGCTATACGCTGATGCAGCAATGCTGCATCGG
GLPV_RNA2_alyu302      TTCGAGTCTCATGAGGCTCATTAGGAACAGCTATACGCTGATGCAGCAATGCTGCATCGG
                       * *** *********  ******** **********************************

GLPV_RNA3_alyu302      TCTCTAGGGGAGACCA
GLPV_RNA1_alyu302      TCTCTAGGGGAGACCA
GLPV_RNA2_alyu302      TCTCTAGGGGAGACCA
                       ****************
```

Host: **grapevine**

>MT319109.1 Grapevine line pattern virus isolate Baco22A segment RNA1, complete sequence

>MT319110.1 Grapevine line pattern virus isolate Baco22A segment RNA2, complete sequence

>MT319111.1 Grapevine line pattern virus isolate Baco22A segment RNA3, complete sequence

Alignment of 5’ and 3’-termini of genomic RNAs

5’-terminus

```
MT319111.1 RNA3 GT----AGTCTTTTCTTTTGCTTTAAGT-------------------TTGCATAGCAT--
MT319109.1 RNA1 GTTTGATGCAACTCTTTTTGGTTCAATTCCAATTGATTGTATTGCTCTTACACACCATTT
MT319110.1 RNA2 GTTTGATGCAACTCTTTTTGGTTCAATTCCAATTGATTGTATTGCTCTTACACACCGTTT
                **     *    *  ***** ** ** *                   ** ** * * *

MT319111.1 RNA3 ----AAGTTGAGTCAATATTTGCCTACAATAAC------TGGTTTTCAAGTCTTCGA---
MT319109.1 RNA1 CTCAGCGACGAGATGGCTTTTAGTTTTAATGTTCG----TGACCTTGTTGACGCCGAGGG
MT319110.1 RNA2 CTCAACGCCGAAATGG-ATTTGTTTTCAATGTTTAATTCTGGTCTTCTTACCGCTGA---
                      *  **       ***   *  ***         **   **     *   **
```

3’-terminus with a conserved tRNA-like structure NOT ending with the universal CCA required for aminoacylation

```
MT319111.1      TGCGGGTGTTCAAGTTTTAACAGTTTCTTTAAACTGTAGTCGTTGCTGAAACGCAGTTGA
MT319109.1      --------------------------CTATAACTCATAGTCGTTGTTGTAACGCAGTTGA
MT319110.1      ----------------------------CTAAACTGTAGTCGTTGCTGAAACGCAGTCGA
                                             ***    ********* ** ******** **

MT319111.1      TCGGGTACTATAACTCGAAGTCGTGGTTGACACGCCAGTTACTTATACAAGAGGTATAAG
MT319109.1      TTGGGTACTATAACCCAAAGTCGTGGTTGACACGCCTTTTGCTTATATAAGAGATGTAAG
MT319110.1      TTGGGTACTATAACCCAAAGTCGTGGTTGACACGCCTTTTGCTTATATAAGAGATGTAAG
                * ************ * *******************  ** ****** ***** * ****

MT319111.1      TGATTGAGTATAGGAATCCATT-CTCCTCGTTGGATGCATCTGTCCGAATCTCATGAGAT
MT319109.1      TAATTGAGTATAGGAATCCGTTTCTTCCCGTCGGATGCATCTGTTCGAGTCTCATGAGGC
MT319110.1      TAATTGAGTATAGGAATCCGTTGCTTCCCGTCGGATGCGTCTGTTCGAGTCTCATGAGAC
                * ***************** ** ** * *** ****** ***** *** *********

MT319111.1      TCATTAGGGACAGCTATACGCTGATGCAGCAATGCTGCATCGGTCTCTAGGGGAGACC
MT319109.1      TCATTAGGAACAGCTATACGTCAATGCAGCAATGCTGCATTGGTCTCTAGGGGAGACC
MT319110.1      TCATTAGGAACAGCTATACGTCAATGCAGCAATGCTGCATTGGTCTCTAGGGGAGACC
                ******** ***********   ***************** *****************
```

Host: ***Rehmannia glutinosa***

>MZ395976.1 Grapevine line pattern virus isolate Rg24 segment RNA1, complete sequence

>MZ395977.1 Grapevine line pattern virus isolate Rg24 segment RNA2, complete sequence

>MZ395978.1 Grapevine line pattern virus isolate Rg24 segment RNA3, complete sequence

Alignment of 5’ and 3’-termini of genomic RNAs

5’-terminus

```
MZ395978.1 RNA3 -------------GTAGTCTTTTCTTTTGCTTTAAGTTTGCATAGCAT------AAGTTG
MZ395976.1 RNA1 TTTTGGTTCAATTCCAATTGATTGTATTGCT-----CTTACGTACCATTTCTCAGCGACG
MZ395977.1 RNA2 -------------CCAATTGATTGTATTGCT-----CTTACACACCGTTTCTCAACGCCG
                               * *   ** * *****      ** *  * * *        *  *

MZ395978.1      AGTCAATATTTGCCTGCAATAACTGGTTTTCAAGTTCTCGAATCTTAGTTGATTTGCTTG
MZ395976.1      AGATGGCTTTTAGTTTTAATGTTCG----TGACCTTGTTGACGCCGAGGGGAGTCAGGCT
MZ395977.1      AAATGG-ATTTGTTTTCAATGTTTAATTCTGGTCTTCTTACCGCTGATT-----------
                *       ***   *  ***         *    ** *     *  *
```

3’-terminus with a conserved tRNA-like structure NOT ending with the universal CCA required for aminoacylation

```
MZ395978.1      GTGTGGTTC---AGGTGTGAGTGTTCAAGT---------TTTAACAGTTTCTTTAAACTG
MZ395976.1      -------TCTTCAAATTTAATTT-TGAAGATGATATTAGTTGAATGAGTACTATAACTCA
MZ395977.1      TCATGATTCTTGAGAAGCATCTCATGAATCAGGTGTTAGTTGAATGAGTACTATAACTCA
                       **   *        *  * **           ** **    * ** ***

MZ395978.1      TAGTCGTTGCTGAAACGCAGTTGATCGAGTACTATAACTCGAAGTCGTGGTTGACACGCC
```

```
MZ395976.1      TAGTCGTTGTTGCAACGCAGTCGATTGGGTACTATAACCCAAAGTCGTGGTTGACACGCC
MZ395977.1      TAGTCGTTGTTGCAACGCAGTCGATTGGGTACTATAACCCAAAGTCGTGGTTGACACGCC
                ********* ** ******** *** * ********** * *******************

MZ395978.1      AGTTGCTTATACAAGAGGTATAAGTGATTGAGTATAGGAATCCATT-CTCCTCGTTGGAT
MZ395976.1      TTTTGCTTATATAAGAGATGTAAGTAATTGAGTATAGCAATCCGTTGCTTCCCGTCGGAT
MZ395977.1      TTTTGCTTATATAAGAGATGTAAGTAATTGAGTATAGGAATCCGTTGCTTCCCGTCGGAT
                  ********* ***** * ***** *********** ***** ** ** * *** ****

MZ395978.1      GCATCTGTCCGAATCTCATGAGATTCATTAGGGACAGCTATACGCTAATGCAGCAATGCT
MZ395976.1      GCATCTGTTCGAGTCTCATGAGGCTCATTAGGAACAGCTATACGTCAATGCAGCAATGCT
MZ395977.1      GCATCTGTTCGAGTCTCATGAGGCTCATTAGGAACAGCTATACGTCAATGCAGCAATGCT
                ******** *** *********  ******** ***********  **************

MZ395978.1      GCATCGGTCTCTAGGGGAGACC
MZ395976.1      GCATTGGTCTCTAGGGGAGACC
MZ395977.1      GCATTGGTCTCTAGGGGAGACC
                **** *****************
```

Host: ***Cannabis sativa***

>MW888424.1 Grapevine line pattern virus isolate GLPV-Lar segment RNA1 methyltransferase gene, complete cds

>MW888423.1 Grapevine line pattern virus isolate GLPV-Lar segment RNA2 RNA-dependent RNA polymerase gene, complete cds

>MW888422.1 Grapevine line pattern virus isolate GLPV-Lar segment RNA3 movement protein gene, complete cds

Alignment of 5'- and 3'-termini

5'-terminus

```
MW888422.1 RNA3 CTT---TTAAGTTTGCATAGCATAAGTTG-----AGTCAATATTTGCCTGCAAT-AACTG
MW888424.1 RNA1 TTTTGGTTCAATTCCAATTGATTGTATTGCTCTTACATACCATTTCTCAGCGACGAGATG
MW888423.1 RNA2 ------TTCAATTCCAATTGATTGTATTGCTCTTACACACCGTTTCTCAACGCCGAAATG
                      ** * **   ** *  *   ***     *   *   ***  *  *    *  **

MW888422.1      GTTTTCAAGTCTTCGAATCTTAGT--TGATTTGCTTGATTTCCG---------------
MW888424.1      GCTTTTAG--TTTTAATGTTCG----TGACCTTGTTGACGCCGAGGGGAGTCAGGCTATC
MW888423.1      GATTT---GTTTTCAATGTTTAATTCTGGTCTTCTTACCGCTGA---------------
                * ***      **  *   *      **   *  **
```

3'-terminus with a conserved tRNA-like structure NOT ending with the universal CCA required for aminoacylation

```
MW888422.1      G--TTC-----AGGTGT----------GGGTGTTCAAGTTTTAACAGTTTCTTTAAACTG
MW888424.1      -CCTTC-----AAATTTAATTTGA---AGGTATTATAAGTTGAATGAGTACTATAACTCA
MW888423.1      A--TTCTTGAGAAATATCTCATGAATCAGGTGTT---AGTTGAATGAGTACTATAACTCA
                   ***     *  * *           *** **     ** **    * ** ***

MW888422.1      TAGTCGTTGCTGAAACGCAGTTGATCGAGTACTATAACTCGAAGTCGTGGTTGACACGCC
MW888424.1      TAGTCGTTGTTATAACGCAGTTGATTGGGTACTATAACCCAAAGTCGTGGTTGACACGCC
MW888423.1      TAGTCGTTGTTGCAACGCAGTTGATTGGGTACTATAACCCAAAGTCGTGGTTGACACGCC
                ********* *  ************ * ********** * *******************

MW888422.1      AGTTACTTATACAAGAGGTATAAGTGATTGAGTATAGGAATCCATTCTCCTCGTTGGATG
MW888424.1      TTTTGCTTATATAAGAGATTTAAGTAATTGAGTATAGGAATCCGTTCTTCCCGTCGGATG
MW888423.1      TTTTGCTTATATAAGAGATTTAAGTAATTGAGTATAGGAATC------------------
                  ** ****** ***** * ***** ***************

MW888422.1      CATCTGTCCGAATCTCATGAGATTCATCAGGGACAGCTATACGCTGATGCAGCAATGCTG
MW888424.1      CATCTGTTCGAGTCTCATGAGGCTCATTAGGAACAGCTATACGCTGATGCAGCAATGCTG
MW888423.1      ------------------------------------------------------------


MW888422.1      CATCGGTCTCTAGGGGA
MW888424.1      CATCGGTCTCT------
MW888423.1      -----------------
```

(D) Canabis sativa mitovirus 1 (CasaMV1)

>CasaMV1_K_alyu311_312_313
TGGTACCATCGGGAGCTTTCATGTTCCCGACGGGGTGTGGTTTCCCACCACCCGACACATTTGAGTACATAGTGAAGATAATTATCAAAATTAAAACTTATCTTCCATGGAAATTATGTGTTTACCTCCAAGAAGGCATCCACACGGGTT
GATGGATATATTGATTAATCGGTATATCCACAATAACTTGTGGACAACCTTAACAGGTTACTAGACCCAATAATGAATCTAGTAACACTGGATCCAGTATAGGGATATCCTGGACGCCCCCCATCATTCGGACTTGATCCGAAGGTTAAG
GAGAGAAGGATTCTACCGGAGGCTACTCCACCAAGTTGCGGTACTGAGTTTAATTCGTTGTTATACTAGATTTCCTCCTAGTTATTAGAGGATTTAGTCTCTTTTGACTAGGATTGAATCTAATATAATAATAAAATT**ATG**CTCTTTACC
AACTTTGTGGAGCGCCTAAGGCAAGAACCTTGGCGTGGAATGTTTGAACATTTCAGGTTACTGCGTGGGTTCCTTATCAGAATCCACACAGTAATCGCCAGTCACCCTACTCTTGAGGTTGCGATCGCTTCAATTAGCTTCGCCCCGCAAA
TGTAAACATATAAGTAAACACTCTGGTCTTCTTTTCACTGCGCTTTATTTAAAGCAGTGCTCCGTCTCTTTACAAAGGTATTATTCCCAATGTGAGGAGTTGGAGGGTGACTCCATGAGTGTATATGTTTCACTGAGCCGTTCAGGCATA
CCGCGGATCATCCCGAAGCATCACCGTAACCAAATTAGGTTAAGGAATGCTCGTTCAGATTATCTCGTTCGAGTCTATTTATCTTGGTTTTCTGTAGCTAAGATCGTGAAACTAGCGAAGCCTGTTAAAGCTTCTCTGTTTCAGTCTATC
GTACAAGAGCCAAAAGATATTGATTCGGTACGAGGAGTGTTAGGAAGAATGAAACAGAACTTCCCTATACTCCAGCGTTCGTATCTCCCCTGGCTCACTAAAGTACCTTTAAAGAAAGGTATCAAGTGGGTTCCTACGTGGAAGTCCACT
CCAAATGATGATAGGCAGTTCCCTTCTCCTAATGTTGAGGATCGGGTTCCGACTATCTTTACTTCTTTGAAGCATGAGATGGCGGCATTCGCTCACCAACTCAATGTCATTCATTCATTTGAGGGTGTGTTTTCTCCAGGTATCCTCTTT
CGTGAGAGAACCATGTGGCCTCTCCAATATAGAGAGAATACTCGGATATGCAATGAAGATTTGGATTATTATGAGCGCTCTACCTTAGCGGTAGGTTTTCAATCTATTGCGTCTGCTTATGATACCAATTGGTTGCATTTCGGTAGGATT
GCTCAGTCGGTGGAAGGAGGAGGAAAACGACGTTTATTTGGCATAGGAAACTATGTTAAACAGCGTCTTCTCCAACCTGTGCATGATTGGGCTATGCAGGTTTTATCCCGCATACCCATGGATGGGACCTTTGATCAAAGTGCTCCCATA
CATCGGTTAGCCAGACTAGAGACAACAGGCTTTATAGCTTCGTTCGATTTAAAATCTGCTACTGACCGATGGCCAGTTTCGGTTATTCATGATGTGTTCGCACTCATGTTTAGCCCAACATTGGCTTCATGTGTGGTCAATGGTTGTCTT
GCTTTAAACGTCTTTTCGGTGTTGCCTCCGTTTGTACGGAAGCCATCAAAAGTATGTTTTAAAACAGGCCAGCCATTAGGTTATTTATCCTCTTGGCCGTTGTTTGCGTTATCCCACCACTATATTGTGTGGTTGGCGGCAAAGCAAGCT
TATCCTAATCTGGATGTGTTCGACCGGTATGCCCTTCTGGGTGACGATATTGTCATTGCAGATAGGGCAGTGGCAGAACAGTATCGACAGATATTAGAAGAACTGGGGGTAACCATATCATTCTCAAAATCAATTGTGAGTGATTATGGT
TGCCTTGAGTTCGCGAAGCAGTTTTGGGTGAAAGACATAAAAGTGAATCTTTCACCCGTATCTGCTCCTTCTGTTCTAGTATCGAGAACGGTCATGGGAATCACTCAGTTGGCTGATAAGTATGAGGTTAAATCAGCCTCAGGCTTATTA
CGCCTTGCTGGTGCTGGTTTCCGTGTTCGATCTCAGATGATGTCTCTCAAGTTAGCTTCAAAGTGGAGACGTCTTCTTGTTGTGGCTAGTAAGCCGCCGAAGAAGTTCGAATCAGCTTTGCATTGGTGGGTTGCAGGAGGTAAACCTCTC
AATCCTTATGTGAAGGCGATAATGGTTCGTCTGTTCATTAGTCAACTGAAGCCTAAAGAACCTCAGTTGCCTATGAACCGCATGTTCGAGGGTGAAGCGACAGTATCTGAATATATGCTTACGCGCAATTGGGTTTCCCAATGGCTAAAG
TGGTTATCTTGGTATTGTCGTGTAGCTCTTGGTCCAGATCCAACAATATCTGCATTGCTGGATGCTCCTGTAGTGGCATCAAAATGGCAGCGCTCAACTTTTGATATTACTATCAAGAAGTTTGGGTTGCTATGGAGGTGTTACGATATA
GGAATTCAGTACACTGTTGATTGGTATCCAGGAGTCCTGGAACCGTTAATAGTGGGTCTCTTACCGTGTTGGTATAATTTGAGTTACCAACCTGTAGGATATTCTGTATCTATCGTTGATGGTATTGGCTATGTATCGCACCATAGTGTC
GCTGTTTACCTAATCAGTAAACAG**TGA**GTAAATAGGGTGACTGCATGTAAGAAATTAGGTACATCTGAGTACATAGTATCCTGTACAAGTACTCTTCTTCGGAAGGGGGGGGTGTGCAGGATCATAGGAACCGAATGATTAATCGGTTCCT
AAACC

>CasaMV1_Sweet6_alyu314
TGGTACCATCGGGAGCTTTCATGTTCCCGACGGGGTGTGGTTTCCCACCACCCGACACATTTGAGTACATAGTGAAGATAATTATCAAAATTAAAACTTATCTTCCATGGAAATTATGTGTTTACCTCCAAGAAGGCATCCACACGGGTT
GATGGATATATTGATTAATCGGTATATCCACAATAACTTGTGGACAACCTTAACAGGTTACTAGACCCAATAATGAATCTAGTAACACTGGATCCAGTATAGGGATATCCTGGACGCCCCCCATCATTCGGACTTGATCCGAAGGTTAAG
GAGAGAAGGATTCTACCGGAGGCTACTCCACCAAGTTGCGGTACTGAGTTTAATTCGTTGTTATACTAGATTTCCTCCTAGTTATTAGAGGATTTAGTCTCTTTTGACTAGGATTGAATCTAATATAATAATAAAATT**ATG**CTCTTTACC
AACTTTGTGGAGCGCCTAAGGCAAGAACCTTGGCGTGGAATGTTTGAACATTTCAGGTTACTGCGTGGGTTCCTTATCAGAATCCACACAGTAATCGCCAGTCACCCTACTCTTGAGGTTGCGATCGCTTCAATTAGCTTCGCCCCGCAAA
TGTAAACATATAAGTAAACACTCTGGTCTTCTTTTCACTGCGCTTTATTTAAAGCAGTGCTCCGTCTCTTTACAAAGGTATTATTCCCAATGTGAGGAGTTGGAGGGTGACTCCATGAGTGTATATGTTTCACTGAGCCGTTCAGGCATA
CCGCGGATCATCCCGAAGCATCACCGTAACCAAATTAGGTTAAGGAATGCTCGTTCAGATTATCTCGTTCGAGTCTATTTATCTTGGTTTTCTGTAGCTAAGATCGTGAAACTAGCGAAGCCTGTTAAAGCTTCTCTGTTTCAGTCTATC
GTACAAGAGCCAAAAGATATTGATTCGGTACGAGGAGTGTTAGGAAGAATAAAACAGAACTTCCCTATACTCCAGCGTTCGTATCTCCCCTGGCTCACTAAAGTACCTTTAAAGAAAGGTATCAAGTGGGTTCCTACGTGGAAGTCCACT
CCAAATGATGATAGGCAGTTCCCTTCTCCTAATGTTGAGGATCGGGTTCCGACTATCTTTACTTCTTTGAAGCATGAGATGGCGGCATTCGCTCACCAACTCAATGTCATTCATTCATTTGAGGGTGTGTTTTCTCCAGGTATCCTCTTT
CGTGAGAGAACCATGTGGCCTCTCCAATATAGAGAGAATACTCGGATATGCAATGAAGATTTGGATTATTATGAGCGCTCTACCTTAGCGGTAGGTTTTCAATCTATTGCGTCTGCTTATGATACCAATTGGTTGCATTTCGGTAGGATT
GCTCAGTCGGTGGAAGGAGGAGGAAAACGACGTTTATTTGGCATAGGAAACTATGTTAAACAGCGTCTTCTCCAACCTGTGCATGATTGGGCTATGCaggTTTTATCCCGCATACCCATGGATGGGACCTTTGATCAAAGTGCTCCCATA
CATCGGTTAGCCAGACTAGAGACAACAGGCTTTATAGCTTCGTTCGATTTAAAATCTGCTACTGACCGATGGCCAGTTTCGGTTATTCATGATGTGTTCGCACTCATGTTTAGCCCAACATTGGCTTCATGTGTGGTCAATGGTTGTCTT
GCTTTAAACGTCTTTTCGGTGTTGCCTCCGTTTGTACGGAAGCCATCAAAAGTATGTTCTTCAACAGGCCAGCCATTAGGTTATTTATCCTCTTGGCCGTTGTTTGCGTTATCCCACCACTATATTGTGTGGTTGGCGGCAAAGCAAGCT
TATCCTAATCTGGATGTGTTCGACCGGTATGCCCTTCTGGGTGACGATATTGTCATTGCAGATAGGGCAGTGGCAGAACAGTACCGACAGATATTAGAAGAACTGGGGGTAACCATATCATTCTCAAAATCAATTGTGAGTGATCATGGT
TGCCTTGAGTTCGCGAAGCAGTTTTGGGTAAAAGACATAAAAGTGAATCTTTCACCCGTATCTGCTCCTTCTGTTCTAGTATCGAGAACGGTTATGGGAATCACTCAGTTGGCTGATAAGTATGAGGTTAAATCAGCCTCAGGCTTATTA
CGCCTTGCTGGTGCTGGTTTCCGTGTTCGATCTCAGATGATGTCTCTCAAGTTAGCTTCAAAGTGGAGACGTCTTCTTGTTGTGGCTAGTAAGCCGCCGAAGAAGTTCGAATCAGCTTTGCATTGGTGGGTTGCAGGAGGTAAACCTCTC
AATCCTTATGTGAAGGCGATAATGGTTCGTCTGTTCATTAGTCAACTGAAGCCTAAAGAACCTCAGTTGCCTATGAACCGCATGTTCGAGGGTGAAGCGACAGTATCTGAATATATGCTTACGCGCAATTGGGTTTCCCAATGGCTAAAG
TGGTTATCTTGGTATTGTCGTGTAGCTCTTGGTCCAGATCCAACAATATCTGCATTGCTGGATGCTCCTGTAGTGGCATCAAAATGGCAGCGCTCAACTTTTGATATTACTATCAAGAAGTTTGGGTTGCTATGGAGGTGTTACGATATA
GGAATTCAGTACGCTGTTGATTGGTATCCAGGAGTCCTGGAACCGTTAATAGTGGGTCTCTTACCGTGTTGGTATAATTTGAGTTACCAACCTGTAGGATATTCTGTATCTATTGTTGATGGTATTGGCTATGTATCGCACCATAGTGTC
GCTGTTTACCTAATCAGTAAACAG**TGA**GTAAATAGGGTGACTGCATGTAAGAAATTAGGTACATCTGAGTACATAGTATCCTGTACAAGTACTCTTCTTCGGAAGGGGGGGGTGTGCAGGATCATAGGAACCGAATGATTAATCGGTTCCT
AAACc

>CasaMV1_newconsensus_sample311_312_313_314

>MT878084.1 Cannabis sativa mitovirus 1 isolate Bld1RdRp RNA-dependent RNA polymerase gene, complete cds

>MT878083.1 Cannabis sativa mitovirus 1 isolate Bld2RdRp RNA-dependent RNA polymerase gene, complete cds

>MT878082.1 Cannabis sativa mitovirus 1 isolate ConeRdRp RNA-dependent RNA polymerase gene, complete cds

>MT878081.1 Cannabis sativa mitovirus 1 isolate DeltaRdRp RNA-dependent RNA polymerase gene, complete cds

>MT878080.1 Cannabis sativa mitovirus 1 isolate RBRdRp RNA-dependent RNA polymerase gene, complete cds

>BK010437.1 TPA_inf: Cannabis sativa mitovirus 1 strain CasaMV1-MPC/MSU RNA-dependent RNA polymerase (RdRp) gene, complete cds

>BK010438.1 TPA_inf: Cannabis sativa mitovirus 1 strain CasaMV1-Finola RNA-dependent RNA polymerase (RdRp) gene, complete cds

>BK010436.1 TPA_inf: Cannabis sativa mitovirus 1 strain CasaMV1-UC-COE RNA-dependent RNA polymerase (RdRp) gene, complete cds

>BK010428.1 TPA_inf: Cannabis sativa mitovirus 1 strain CasaMV1-PurpleKush RNA-dependent RNA polymerase (RdRp) gene, complete cds

CLUSTAL multiple sequence alignment by MUSCLE (3.8)

```
MT878083.1                            --------------GAGCATTCATGTTCCCGACGGGGTGTGGTTTCCCACCACCCGACAC
MT878082.1                            --------------GAGCATTCATGTTCCCGACGGGGTGTGGTTTCCCACCACCCGACAC
MT878081.1                            -----------------CATTCATGTTCCCGACGGGGTGTGGTTTCCCACCACCCGACAC
MT878080.1                            --------------GAGCATTCATGTTCCCGACGGGGTGTGGTTTCCCACCACCCGACAC
MT878084.1                            --------------GAGCTTTCATGTTCCCGACGGGGTGTGGTTTCCCACCACCCGACAC
BK010428.1                            GGGGGTACCATCGGGAGCTTTCATGTTCCCGACGGGGTGTGGTTTCCCACCACCCGACAC
BK010437.1                            -----------CGGGAGCTTTCATGTTCCCGACGGGGTGTGGTTTCCCACCACCCGACAC
CasaMV1_newconsensus_sample311_3      --TGGTACCATCGGGAGCTTTCATGTTCCCGACGGGGTGTGGTTTCCCACCACCCGACAC
BK010438.1                            -----------------CTTTCATGTTCCCGACGGGGTGTGGTTTCCCACCACCCGACAC
BK010436.1                            ----------TCGGGAGCTTTCATGTTCCCGACGGGGTGTGGTTTCCCACCACCCGACAC
                                                       * *****************************************

MT878083.1                            AATATGAGTACATAGTGAAGATAACCAACAAAATTA-AACTTATCTTCCTAGGAAAGTGT
MT878082.1                            AATATGAGTACATAGTGAAGATAACCAACAAAATTA-AACTTATCTTCCTAGGAAAGTGT
MT878081.1                            AATATGAGTACATAGTGAAGATAACCAACAAAATTA-AACTTATCTTCCTAGGAAAGTGT
MT878080.1                            AATATGAGTACATAGTGAAGATAACCAACAAAATTA-AACTTATCTTCCTAGGAAAGTGT
MT878084.1                            -ATTTGAGTACATAGTGAAGATAATTATCAAAATTAAAACTTATCTTCCATGGAAATTAT
BK010428.1                            -ATTTGAGTACATAGTGAAGATAATTATCAAAATCAAAACTTATCTTCCATGGAAATTAT
BK010437.1                            -ATTTGAGTACATAGTGAAGATAATTATCAAAATTAAAACTTATCTTCCATGGAAATTAT
CasaMV1_newconsensus_sample311_3      -ATTTGAGTACATAGTGAAGATAATTATCAAAATTAAAACTTATCTTCCATGGAAATTAT
BK010438.1                            -ATTTGAGTACATAGTGAAGATAATTATGAAAATTAGAACTTATCTTCCATGGAAATCAT
BK010436.1                            -ATTTGAGTACATAGTGAAGATAATTATCAAAATTAAAACTTATCTTCCATGGAAATCAT
                                       ** ********************  *  ***** * ************  *****   *

MT878083.1                            GTGTCTACCTCCAAGGAGGCATCCACACGGGTTGATGGATATATTGATTAATCAGTCTAT
MT878082.1                            GTGTCTACCTCCAAGGAGGCATCCACACGGGTTGATGGATATATTGATTAATCAGTCTAT
MT878081.1                            GTGTCTACCTCCAAGGAGGCATCCACACGGGTTGATGGATATATTGATTAATCAGTCTAT
MT878080.1                            GTGTCTACCTCCAAGGAGGCATCCACACGGGTTGATGGATATATTGATTAATCAGTCTAT
MT878084.1                            GTGTTTACCTCCAAGAAGGCATCCACACGGGTTGATGGATATATTGATTAATCGGTATAT
BK010428.1                            GTGTTTACCTCCAAGAAGGCATCCACACGGGTTGATGGATATATTGATTAATCGGTATAT
BK010437.1                            GTGTTTACCTCCAAGAAGGCATCCACACGGGTTGATGGATATATTGATTAATCGGTATAT
CasaMV1_newconsensus_sample311_3      GTGTTTACCTCCAAGAAGGCATCCACACGGGTTGATGGATATATTGATTAATCGGTATAT
BK010438.1                            GTGTTTACCTCCAAGAAGGCATCCACACGGGTTGATGGATATATTGATTAATCGGTATAT
BK010436.1                            GTGTTTACCTCCAAGAAGGCATCCACACGGGTTGATGGATATATTGATTAATCGGTATAT
                                      **** ********** ************************************* ** ***

MT878083.1                            CCACAATAACTTGTGGACAACCTTAATAGTTATCTAGACCAAAGAATGAATCTAGATAAA
MT878082.1                            CCACAATAACTTGTGGACAACCTTAATAGTTATCTAGACCAAAGAATGAATCTAGATAAA
MT878081.1                            CCACAATAACTTGTGGACAACCTTAATAGTTATCTAGACCAAAGAATGAATCTAGATAAA
MT878080.1                            CCACAATAACTTGTGGACAACCTTAATAGTTATCTAGACCAAAGAATGAATCTAGATAAA
MT878084.1                            CCACAATAACTTGTGGACAACCTTAACAGGTTACTAGACCCAATAATGAATCTAGTAACA
BK010428.1                            CCACAATAACTTGTGGACAACCTTAACAGGTTACTAGACCCAATAATGAATCTAGTAACA
BK010437.1                            CCACAATAACTTGTGGACAACCTTAACAGGTTACTAGACCCAATAATGAATCTAGTAACA
CasaMV1_newconsensus_sample311_3      CCACAATAACTTGTGGACAACCTTAACAGGTTACTAGACCCAATAATGAATCTAGTAACA
BK010438.1                            CCACAATAACTTGTGGACAACCTTAACAGATTACTAGACCCAATAATGAATCTAGTAATA
BK010436.1                            CCACAATAACTTGTGGACAACCTTAACAGGTTACTAGACCCAATAATGAATCTAGTAATA
                                      ************************** ** *  ******* ** ***********  * *

MT878083.1                            CTAGATCCAGTATAGGGATA-CCTGGACGCCCCCCATCACTTGGACTTGATCCAAGGGTT
MT878082.1                            CTAGATCCAGTATAGGGATA-CCTGGACGCCCCCCATCACTTGGACTTGATCCAAGGGTT
MT878081.1                            CTAGATCCAGTATAGGGATA-CCTGGACGCCCCCCATCACTTGGACTTGATCCAAGGGTT
MT878080.1                            CTAGATCCAGTATAGGGATA-CCTGGACGCCCCCCATCACTTGGACTTGATCCAAGGGTT
MT878084.1                            CTGGATCCAGTATAGGGATATCCTGGACGCCCCCCATCATTCGGACTTGATCCGAAGGTT
BK010428.1                            CTGGATCCAGTATAGGGATATCCTGGACGCCCCCCATCATTCGGACTTGATCCGAAGGTT
BK010437.1                            CTGGATCCAGTATAGGGATATCCTGGACGCCCCCCATCATTCGGACTTGATCCGAAGGTT
CasaMV1_newconsensus_sample311_3      CTGGATCCAGTATAGGGATATCCTGGACGCCCCCCATCATTCGGACTTGATCCGAAGGTT
BK010438.1                            CTGGATCCAGTATAGGGATATCCTGGACGCCCCCCATCATTCGGACTTGATCCGAAGGTT
BK010436.1                            CTGGATCCAGTATAGGGATATCCTGGACGCCCCCCATCATTCGGACTTGATCCGAAGGTT
                                      ** ***************** ****************** * *********** * ****

MT878083.1                            AAGGAGAGAA-GATTCTACCGGAGGCTACTCCACCAAGTTGCTGTAATGA-CATGATTCG
MT878082.1                            AAGGAGAGAA-GATTCTACCGGAGGCTACTCCACCAAGTTGCTGTAATGA-CATGATTCG
MT878081.1                            AAGGAGAGAA-GATTCTACCGGAGGCTACTCCACCAAGTTGCTGTAATGA-CATGATTCG
MT878080.1                            AAGGAGAGAA-GATTCTACCGGAGGCTACTCCACCAAGTTGCTGTAATGA-CATGATTCG
MT878084.1                            AAGGAGAGAAGGATTCTACCGGAGGCTACTCCACCAAGTTGCGGTACTGAGTTTAATTCG
BK010428.1                            ACGGAGAGAAGGATTCTACCGGAGGCTACTCCACCAAGTTGCGGTACTGAGTTTAATTCG
BK010437.1                            AAGGAGAGAAGGATTCTACCGGAGGCTACTCCACCAAGTTGCGGTACTGAGTTTAATTCG
CasaMV1_newconsensus_sample311_3      AAGGAGAGAAGGATTCTACCGGAGGCTACTCCACCAAGTTGCGGTACTGAGTTTAATTCG
BK010438.1                            AAGGAGAGAAGGATTCTACCGGAGGCTACTCCACCAAGTTGCTGTAATGAGTTTAATTCG
BK010436.1                            AAGGAGAGAAGGATTCTACCGGAGGCTACTCCACCAAGTTGCGGTACTGAGTTTAATTCG
                                      * ******** ******************************* *** ***   * *****

MT878083.1                            TTGGTATACTAGACTTCCTCCTAGTCATTAGAGGATTTAGTC-CCTTGTGACTAGGATTG
MT878082.1                            TTGGTATACTAGACTTCCTCCTAGTCATTAGAGGATTTAGTC-CCTTGTGACTAGGATTG
MT878081.1                            TTGGTATACTAGACTTCCTCCTAGTCATTAGAGGATTTAGTC-CCTTGTGACTAGGATTG
MT878080.1                            TTGGTATACTAGACTTCCTCCTAGTCATTAGAGGATTTAGTC-CCTTGTGACTAGGATTG
MT878084.1                            TTGTTATACTAGATTTCCTCCTAGTTATTAGAGGATTTAGTC-TCTTTTGACTAGGATTG
BK010428.1                            TTGTTATACTAGATTTCCTCCTAGTTATTAGAGGATTTAGTC-TCTTTTGACTAGGATTG
BK010437.1                            TTGTTATACTAGATTTCCTCCTAGTTATTAGAGGATTTAGTC-TCTTTTGACTAGGATTG
CasaMV1_newconsensus_sample311_3      TTGTTATACTAGATTTCCTCCTAGTTATTAGAGGATTTAGTC-TCTTTTGACTAGGATTG
BK010438.1                            TTGTTATACTAGATTTCCTCCTAGTTATTTGAGGATTTAGTCTTTTTATGACTAGGATTG
BK010436.1                            TTGTTATACTAGAGTTCCTCCTAGTTATTAGAGGATTTAGTC-TCTTATGACTAGGATTG
                                      *** ********* *********** *** ************   ** ************

MT878083.1                            AATCAAAATATATTAGCG-TATCATGCTTTTCACCAACTTTGTGGAGCGCCTAAGGCAAG
MT878082.1                            AATCAAAATATATTAGCG-TATCATGCTTTTCACCAACTTTGTGGAGCGCCTAAGGCAAG
MT878081.1                            AATCAAAATATATTAGCG-TATCATGCTTTTCACCAACTTTGTGGAGCGCCTAAGGCAAG
MT878080.1                            AATCAAAATATATTAGCG-TATCATGCTTTTCACCAACTTTGTGGAGCGCCTAAGGCAAG
MT878084.1                            AATC-TAATATAATAATGAAATTATGCTCTTTACCAACTTTGTGGAGCGCCTAAGGCAAG
BK010428.1                            AATC-TAATATAATAATAAAATTATGCTCTTTACCAACTTTGTGGAGCGCCTAAGGCAAG
BK010437.1                            AATC-TAATATAATAATAAAATTATGCTCTTTACCAACTTTGTGGAGCGCCTAAGGCAAG
CasaMV1_newconsensus_sample311_3      AATC-TAATATAATAATAAAATTATGCTCTTTACCAACTTTGTGGAGCGCCTAAGGCAAG
BK010438.1                            AATC-TAATATAATAATGAAATTATGCTCTTTACCAACTTTGTGGAGCGCCTAAGGCAAG
BK010436.1                            AATC-TAATATAATAATGAAATTATGCTCTTTACCAACTTTGTGGAGCGCCTAAGGCAAG
                                      ****  ****** **     ** ***** ** ****************************

MT878083.1                            AACCTTGGCGTGGAATGTTTGAACATTTCAGGTTACTGCGTGGGTTCCTTATCAGAATTC
MT878082.1                            AACCTTGGCGTGGAATGTTTGAACATTTCAGGTTACTGCGTGGGTTCCTTATCAGAATTC
MT878081.1                            AACCTTGGCGTGGAATGTTTGAACATTTCAGGTTACTGCGTGGGTTCCTTATCAGAATTC
MT878080.1                            AACCTTGGCGTGGAATGTTTGAACATTTCAGGTTACTGCGTGGGTTCCTTATCAGAATTC
MT878084.1                            AACCTTGGCGTGGAATGTTTGAACATTTCAGGTTACTGCGTGGGTTCCTTATCAGAATCC
BK010428.1                            AACCTTGGCGTGGAATGTTTGAACATTTCAGGTTACTGCGTGGGTTCCTTATCAGAATCC
BK010437.1                            AACCTTGGCGTGGAATGTTTGAACATTTCAGGTTACTGCGTGGGTTCCTTATCAGAATCC
CasaMV1_newconsensus_sample311_3      AACCTTGGCGTGGAATGTTTGAACATTTCAGGTTACTGCGTGGGTTCCTTATCAGAATCC
BK010438.1                            AACCTTGGCGTGGAATGTTTGAACATTTCAGGTTACTGCGTGGGTTCCTTATCAGGATCC
```

```
BK010436.1                          AACCTTGGCGTGGAATGTTTGAACATTTCAGGTTACTGCGTGGGTTCCTTATCAGAATCC
                                    ******************************************************* ** *

MT878083.1                          ACACAGTAATCGCCAGTCACCCTACTCTCGAGGTTGCGATCGCTTCAATTAGCTTTGCTC
MT878082.1                          ACACAGTAATCGCCAGTCACCCTACTCTCGAGGTTGCGATCGCTTCAATTAGCTTTGCTC
MT878081.1                          ACACAGTAATCGCCAGTCACCCTACTCTCGAGGTTGCGATCGCTTCAATTAGCTTTGCTC
MT878080.1                          ACACAGTAATCGCCAGTCACCCTACTCTCGAGGTTGCGATCGCTTCAATTAGCTTTGCTC
MT878084.1                          ACACTGTAATCGCCAGTCACCCTACTCTTGAGGTTGCGATCGCTTCAATTAGCTTCGCCC
BK010428.1                          ACACAGTAATCGCCAGTCACCCTACTCTTGAGGTTGCGATCGCTTCAATTAGCTTCGCCC
BK010437.1                          ACACAGTAATCGCCAGTCACCCTACTCTTGAGGTTGCGATCGCTTCAATTAGCTTCGCCC
CasaMV1_newconsensus_sample311_3    ACACAGTAATCGCCAGTCACCCTACTCTTGAGGTTGCGATCGCTTCAATTAGCTTCGCCC
BK010438.1                          ACACAGTAATCGCCAGTCACCCTACTCTTGAGGTTGCGATCGCTTCAATAAGCTTTGCTC
BK010436.1                          ACACAGTAATCGCCAGTCACCCTACTCTTGAGGTTGCGATCGCTTCAATTAGCTTTGCTC
                                    **** *********************** ******************** ***** ** *

MT878083.1                          GCAAATGTAGACATATAAGTAAACACTCAGGTCTTCTTTTCACTGCGCTTTATCTTAAGC
MT878082.1                          GCAAATGTAGACATATAAGTAAACACTCAGGTCTTCTTTTCACTGCGCTTTATCTTAAGC
MT878081.1                          GCAAATGTAGACATATAAGTAAACACTCAGGTCTTCTTTTCACTGCGCTTTATCTTAAGC
MT878080.1                          GCAAATGTAGACATATAAGTAAACACTCAGGTCTTCTTTTCACTGCGCTTTATCTTAAGC
MT878084.1                          GCAAATGTAAACATATAAGTAAACACTCTGGTCTTCTTTTCACTGCGCTTTATTTAAAGC
BK010428.1                          GCAAATGTAAACATATAAGTAAACACTCTGGTCTTCTTTTCACTGCGCTTTATTTAAAGC
BK010437.1                          GCAAATGTAAACATATAAGTAAACACTCTGGTCTTCTTTTCACTGCGCTTTATTTAAAGC
CasaMV1_newconsensus_sample311_3    GCAAATGTAAACATATAAGTAAACACTCTGGTCTTCTTTTCACTGCGCTTTATTTAAAGC
BK010438.1                          GCAAATGTAAACATATAAGTAAACACTCTGGTCTTCTTTTCACTGCACTTTATTTAAAGC
BK010436.1                          GCAAATGTAAACATATAAGCAAACATTCTGGTCTTCTTTTCACTGCACTTTATTTAAAGC
                                    ********* ********* ***** ** ***************** ****** * ****

MT878083.1                          AGTGCTCTGTTTCCTTACAAAGGTATTATTCCCAATGTGAGAAAATGGAGGGTGACCTTA
MT878082.1                          AGTGCTCTGTTTCCTTACAAAGGTATTATTCCCAATGTGAGAAAATGGAGGGTGACCTTA
MT878081.1                          AGTGCTCTGTTTCCTTACAAAGGTATTATTCCCAATGTGAGAAAATGGAGGGTGACCTTA
MT878080.1                          AGTGCTCTGTTTCCTTACAAAGGTATTATTCCCAATGTGAGAAAATGGAGGGTGACCTTA
MT878084.1                          AGTGCTCCGTCTCTTTACAAAGGTATTATTCCCAATGTGAGGAGTTGGAGGGTGACTCCA
BK010428.1                          AGTGCTCCGTCTCTTTACAAAGGTATTATTCCCAATGTGAGGAGTTGGAGGGTGACTCCA
BK010437.1                          AGTGCTCCGTCTCTTTACAAAGGTATTATTCCCAATGTGAGGAGTTGGAGGGTGACTCCA
CasaMV1_newconsensus_sample311_3    AGTGCTCCGTCTCTTTACAAAGGTATTATTCCCAATGTGAGGAGTTGGAGGGTGACTCCA
BK010438.1                          AGTGCTCCGTCTCCTTACAAAGGTATTATTCCCAATGTGAGAAGTTGGAGGGTGACTCCA
BK010436.1                          AGTGCTCTGTCTCCTTACAAAGGTATTATTCCCAATGTGAGGAGCTAGAGGGTGACACCA
                                    ******* ** ** *************************** *  * *********   *

MT878083.1                          TGAGTGTATATGTCTCCCTGACCCGCACGGGGATACCGCGGATAATCCCGAAGCATCACC
MT878082.1                          TGAGTGTATATGTCTCCCTGACCCGCACGGGGATACCGCGGATAATCCCGAAGCATCACC
MT878081.1                          TGAGTGTATATGTCTCCCTGACCCGCACGGGGATACCGCGGATAATCCCGAAGCATCACC
MT878080.1                          TGAGTGTATATGTCTCCCTGACCCGCACGGGGATACCGCGGATAATCCCGAAGCATCACC
MT878084.1                          TGAGTGTATATGTTTCACTGAGCCGTTCAGGCATACCGCGGATCATCCCGAAGCATCACC
BK010428.1                          TGAGTGTATATGTTTCACTGAGCCGTTCAGGCATACCGCGGATCATCCCGAAGCATCACC
BK010437.1                          TGAGTGTATATGTTTCACTGAGCCGTTCAGGCATACCGCGGATCATCCCGAAGCATCACC
CasaMV1_newconsensus_sample311_3    TGAGTGTATATGTTTCACTGAGCCGTTCAGGCATACCGCGGATCATCCCGAAGCATCACC
BK010438.1                          TGAGTGTATATGTTTCCCTGAGTCGTTCAGGAATACCGCGGATTATCCCGAAGCATCACC
BK010436.1                          TGAGTGTATATGTTTCCCTGAGTCGTTCAGGGATACCGCGGATTATCCCGAAGCATCACC
                                    ************* ** ****  **  * ** *********** ****************

MT878083.1                          GAAACCAAATCAGGTTGAGGACGGCTCGTTCAGATTATCTTGTGAAGGTCTATCTGTCAT
MT878082.1                          GAAACCAAATCAGGTTGAGGACGGCTCGTTCAGATTATCTTGTGAAGGTCTATCTGTCAT
MT878081.1                          GAAACCAAATCAGGTTGAGGACGGCTCGTTCAGATTATCTCGTGAAGGTCTATCTGTCAT
MT878080.1                          GAAACCAAATCAGGTTGAGGACGGCTCGTTCAGATTATCTCGTGAAGGTCTATCTGTCAT
MT878084.1                          GTAACCAAATTAGGTTAAGGAATGCTCGTTCAGATTATCTCGTTCGAGTCTATTTATCTT
BK010428.1                          GTAACCAAATTAGGTTAAGGAATGCTCGTTCAGATTATCTCGTTCGAGTCTATTTATCTT
BK010437.1                          GTAACCAAATTAGGTTAAGGAATGCTCGTTCAGATTATCTCGTTCGAGTCTATTTATCTT
CasaMV1_newconsensus_sample311_3    GTAACCAAATTAGGTTAAGGAATGCTCGTTCAGATTATCTCGTTCGAGTCTATTTATCTT
BK010438.1                          GGAACCAAATCAGGTTAAGGAATGCTCGTTCAGATTATCTCGTTCGAGTCTATTTATCTT
BK010436.1                          GAAACCAAATCAGGTTGAGGAATGCTCGTTCAGATTATCTCGTTCGAGTCTACTTATCTT
                                    * ******** ***** ****  ***************** **    *****  * ** *

MT878083.1                          GGTTTTCTGTAGCTAAGATCGTGAAATTAGCGAAGCCTGTAAAGGCCTCTCTATTTCAGT
MT878082.1                          GGTTTTCTGTAGCTAAGATCGTGAAATTAGCGAAGCCTGTAAAGGCCTCTCTATTTCAGT
MT878081.1                          GGTTTTCTGTAGCTAAGATCGTGAAATTAGCGAAGCCTGTAAAGGCCTCTCTATTTCAGT
MT878080.1                          GGTTTTCTGTAGCTAAGATCGTGAAATTAGCGAAGCCTGTAAAGGCCTCTCTATTTCAGT
MT878084.1                          GGTTTTCTGTAGCTAAGATCGTGAAACTAGCGAAGCCTGTTAAAGCTTCTCTGTTTCAGT
BK010428.1                          GGTTTTCTGTAGCTAAGATCGTGAAACTAGCGAAGCCTGTTAAAGCTTCTCTGTTTCAGT
BK010437.1                          GGTTTTCTGTAGCTAAGATCGTGAAACTAGCGAAGCCTGTTAAAGCTTCTCTGTTTCAGT
CasaMV1_newconsensus_sample311_3    GGTTTTCTGTAGCTAAGATCGTGAAACTAGCGAAGCCTGTTAAAGCTTCTCTGTTTCAGT
BK010438.1                          GGTTTTCTGTAGCTAAGATCGTGAAATTAGCGAAGCCTGTGAAAGCTTCCCTATTTCAGT
BK010436.1                          GGTTTTCTGTAGCTAAGATCGTGAAATTGGCGAAGCCAGTTAAGGCTTCTCTATTTCAGT
                                    ************************** * ******** ** ** ** ** ** *******

MT878083.1                          CTATCGTACAAGAACCAAATGACATCGATCGTGTTCGTGGGGTTCTTGGAAGAATAAAAC
MT878082.1                          CTATCGTACAAGAACCAAATGACATCGATCGTGTTCGTGGGGTTCTTGGAAGAATAAAAC
MT878081.1                          CTATCGTACAAGAACCAAATGACATCGATCGTGTTCGTGGGGTTCTTGGAAGAATAAAAC
MT878080.1                          CTATCGTACAAGAACCAAATGACATCGATCGTGTTCGTGGGGTTCTTGGAAGAATAAAAC
MT878084.1                          CTATCGTACAAGAGCCAAAAGATATTGATTCGGTACGAGGAGTGTTAGGAAGAATAAAAC
BK010428.1                          CTATCGTACAAGAGCCAAAAGATATTGATTCGGTACGAGGAGTGTTAGGAAGAATAAAAC
BK010437.1                          CTATCGTACAAGAGCCAAAAGATATTGATTCGGTACGAGGAGTGTTAGGAAGAATAAAAC
CasaMV1_newconsensus_sample311_3    CTATCGTACAAGAGCCAAAAGATATTGATTCGGTACGAGGAGTGTTAGGAAGAATAAAAC
BK010438.1                          CTATCGTACAAGAGCCAAAAGATATTGATTCGGTACGAGGAGTGTTAGGAAGGATAAAAC
BK010436.1                          CTATCGTACAAGAGCCAAAAGATATTGATTCGGTACGAGGAGTGTTAGGAAGGATAAAAC
                                    ************* ***** ** ** ***   ** ** ** **  * ***** *******

MT878083.1                          AGAGCTTCCCTGAACTCCAACGAGCGTATCTTCCGTGGCTCGCTTCTATACCTTTACATA
MT878082.1                          AGAGCTTCCCTGAACTCCAACGAGCGTATCTTCCGTGGCTCGCTTCTATACCTTTACATA
MT878081.1                          AGAGCTTCCCTGAACTCCAACGAGCGTATCTTCCGTGGCTCGCTTCTATACCTTTACATA
MT878080.1                          AGAGCTTCCCTGAACTCCAACGAGCGTATCTTCCGTGGCTCGCTTCTATACCTTTACATA
MT878084.1                          AAAACTTCCCTATACTCCAGCGTTCGTATCTCCCCTGGCTCACTAAAGTACCTTTAAAGA
BK010428.1                          AGAACTTCCCTATACTCCAGCGTTCGTATCTCCCCTGGCTCACTAAAGTACCTTTAAAGA
BK010437.1                          AGAACTTCCCTATACTCCAGCGTTCGTATCTCCCCTGGCTCACTAAAGTACCTTTAAAGA
CasaMV1_newconsensus_sample311_3    AGAACTTCCCTATACTCCAGCGTTCGTATCTCCCCTGGCTCACTAAAGTACCTTTAAAGA
BK010438.1                          AGAACTTCCCTTCACTCCAGCGCTCGTATCTTCCCTGGCTCACAAAGATACCTTTAAAGA
BK010436.1                          AGAACTTCCCTCGCTCCAGCGTTCGTATCTTCCCTGGCTCACTAATATACCTTTGAAGA
                                    * * *******   ***** **  ******* ** ****** *     *******  * *

MT878083.1                          AGGGCATGAAGTGGGTTCCGACTTGGAAATCCACTCCAAATGATGATCGACAGTTCCCTT
MT878082.1                          AGGGTATGAAGTGGGTTCCGACTTGGAAATCCACTCCAAATGATGATCGACAGTTCCCTT
MT878081.1                          AGGGCATGAAGTGGGTTCCGACTTGGAAATCCACTCCAAATGATGATCGACAGTTCCCTT
MT878080.1                          AGGGCATGAAGTGGGTTCCGACTTGGAAATCCACTCCAAATGATGATCGACAGTTCCCTT
MT878084.1                          AAGGTATCAAGTGGGTTCCTACGTGGAAGTCCACTCCAAATGATGATAGGCAGTTCCCTT
BK010428.1                          AAGGTATCAAGTGGGTTCCTACGTGGAAGTCCACTCCAAATGATGATAGGCAGTTCCCTT
BK010437.1                          AAGGTATCAAGTGGGTTCCTACGTGGAAGTCCACTCCAAATGATGATAGGCAGTTCCCTT
CasaMV1_newconsensus_sample311_3    AAGGTATCAAGTGGGTTCCTACGTGGAAGTCCACTCCAAATGATGATAGGCAGTTCCCTT
BK010438.1                          AAGGTATTAAGTGGGTTCCTACGTGGAAGTCCACTCCAAATGATGATAGGCAATTCCCTT
BK010436.1                          AAGGTATTAAGTGGGTTCCTACGTGGAAGTCCACTCCAAATGATGATAGGCAATTCCCTT
                                    * ** ** *********** ** ***** ****************** * ** *******

MT878083.1                          CTCCTGATGTTGGAGATCGGGTGCCGACTATCTTTACTTCTTTGAAGCATGAGTTAGCGG
MT878082.1                          CTCCTGATGTTGGAGATCGGGTGCCGACTATCTTTACTTCTTTGAAGCATGAGTTAGCGG
MT878081.1                          CTCCTGATGTTGGAGATCGGGTGCCGACTATCTTTACTTCTTTGAAGCATGAGTTAGCGG
MT878080.1                          CTCCTGATGTTGGAGATCGGGTGCCGACTATCTTTACTTCTTTGAAGCATGAGTTAGCGG
MT878084.1                          CTCCTAATGTTGAGGATCGGGTTCCGACTATCTTTACTTCTTTGAAGCATGAGATGGCGG
BK010428.1                          CTCCTAATGTTGAGGATCGGGTTCCGACTATCTTTACTTCTTTGAAGCATGAGATGGCGG
BK010437.1                          CTCCTAATGTTGAGGATCGGGTTCCGACTATCTTTACTTCTTTGAAGCATGAGATGGCGG
CasaMV1_newconsensus_sample311_3    CTCCTAATGTTGAGGATCGGGTTCCGACTATCTTTACTTCTTTGAAGCATGAGATGGCGG
BK010438.1                          CTCCTAATGTTGAGGATCGGGTTCCGACTATCTTTACTTCTTTGAAGCATGAGATGGCGG
BK010436.1                          CTCCTAATGTTGAGGATCGGGTTCCGACTATCTTTACTTCTTTGAAGCATGAGATGGCGG
                                    ***** ******  ******** ****************************** * ****
```

```
MT878083.1                        CTTTCGCTCACCAACTCAATGTCATTCATTCATTTGAGGGTGTGTTTTCTCCAGGTATTC
MT878082.1                        CTTTCGCTCACCAACTCAATGTCATTCATTCATTTGAGGGTGTGTTTTCTCCAGGTATTC
MT878081.1                        CTTTCGCTCACCAACTCAATGTCATTCATTCATTTGAGGGTGTGTTTTCTCCAGGTATTC
MT878080.1                        CTTTCGCTCACCAACTCAATGTCATTCATTCATTTGAGGGTGTGTTTTCTCCAGGTATTC
MT878084.1                        CATTCGCTCACCAACTCAATGTCATTCATTCATTTGAGGGTGTGTTTTCTCCAGGTATCC
BK010428.1                        CATTCGCTCACCAACTCAATGTCATTCATTCATTTGAGGGTGTGTTTTCTCCAGGTATCC
BK010437.1                        CATTCGCTCACCAACTCAATGTCATTCATTCATTTGAGGGTGTGTTTTCTCCAGGTATCC
CasaMV1_newconsensus_sample311_3  CATTCGCTCACCAACTCAATGTCATTCATTCATTTGAGGGTGTGTTTTCTCCAGGTATCC
BK010438.1                        CATTCGCTCACCAACTCAATGTCATTCATTCATTTGAGGGTGTGTTTTCTCCAGGTATCC
BK010436.1                        CATTCGCTCACCAACTCAATGTCATTCATTCATTTGAGGGTGTGTTTTCTCCAGGTATCC
                                  * ******************************************************** *

MT878083.1                        TCTTTCGTGAGAGAACCATGTGGCCTCTCCAATATAGAGAGAATACTCGTATTTGTAACG
MT878082.1                        TCTTTCGTGAGAGAACCATGTGGCCTCTCCAATATAGAGAGAATACTCGTATTTGTAACG
MT878081.1                        TCTTTCGTGAGAGAACCATGTGGCCTCTCCAATATAGAGAGAATACTCGTATTTGTAACG
MT878080.1                        TCTTTCGTGAGAGAACCATGTGGCCTCTCCAATATAGAGAGAATACTCGTATTTGTAACG
MT878084.1                        TCTTTCGTGAGAGAACCATGTGGCCTCTCCAATATAGAGAGAATACTCGGATATGCAATG
BK010428.1                        TCTTTCGTGAGAGAACCATGTGGCCTCTCCAATATAGAGAGAATACTCGGATATGCAATG
BK010437.1                        TCTTTCGTGAGAGAACCATGTGGCCTCTCCAATATAGAGAGAATACTCGGATATGCAATG
CasaMV1_newconsensus_sample311_3  TCTTTCGTGAGAGAACCATGTGGCCTCTCCAATATAGAGAGAATACTCGGATATGCAATG
BK010438.1                        TCTTTCGTGAGAGAACCATGTGGCCTCTCCAATATAGAGAGAATACTCGGATATGTAATG
BK010436.1                        TCTTTCGTGAGAGAACCATGTGGCCTCTCCAATATAGAGAGAATACTCGGTTATGTAATG
                                  *************************************************  * ** ** *

MT878083.1                        AAGATTTGGATTTTTATGAGCGCTCTACTTTAGCCATAGGCTTTCAGTCTGTTGCGTCTG
MT878082.1                        AAGATTTGGATTTTTATGAGCGCTCTACTTTAGCCATAGGCTTTCAGTCTGTTGCGTCTG
MT878081.1                        AAGATTTGGATTTTTATGAGCGCTCTACTTTAGCCATAGGCTTTCAGTCTGTTGCGTCTG
MT878080.1                        AAGATTTGGATTTTTATGAGCGCTCTACTTTAGCCATAGGCTTTCAGTCTGTTGCGTCTG
MT878084.1                        AAGATTTGGATTATTATGAGCGCTCTACCTTAGCGGTAGGTTTTCAATCTATTGCGACTG
BK010428.1                        AAGATTTGGATTATTATGAGCGCTCTACCTTAGCGGTAGGTTTTCAATCTATTGCGTCTG
BK010437.1                        AAGATTTGGATTATTATGAGCGCTCTACCTTAGCGGTAGGTTTTCAATCTATTGCGTCTG
CasaMV1_newconsensus_sample311_3  AAGATTTGGATTATTATGAGCGCTCTACCTTAGCGGTAGGTTTTCAATCTATTGCGTCTG
BK010438.1                        AAGATTTGGATTATTATGAGCGCTCTACTTTAGCAGTAGGTTTTCAATCTATTGCGTCTG
BK010436.1                        AAGATTTGGACTATTATGAGCGCTCTACCTTAGCAGTAGGTTTTCAATCTATTGCATCAG
                                  ********** * *************** *****  **** ***** *** ****  * *
MT878083.1                        CTTATGATTCCTCTTGGTTACATTTCGGTCGGATAGCTCAGTCGGTAGAAGGAGGAGGAA
MT878082.1                        CTTATGATTCCTCTTGGTTACATTTCGGTCGGATAGCTCAGTCGGTAGAAGGAGGAGGAA
MT878081.1                        CTTATGATTCCTCTTGGTTACATTTCGGTCGGATAGCTCAGTCGGTAGAAGGAGGAGGAA
MT878080.1                        CTTATGATTCCTCTTGGTTACATTTCGGTCGGATAGCTCAGTCGGTAGAAGGAGGAGGAA
MT878084.1                        CTTATGATACCAATTGGTTGCATTTCGGTAGGATTGCTCAGTCGGTGGAAGGAGGAGGAA
BK010428.1                        CTTATGATACCAATTGGTTGCATTTCGGTAGGATTGCTCAGTCGGTGGAAGGAGGAGGAA
BK010437.1                        CTTATGATACCAATTGGTTGCATTTCGGTAGGATTGCTCAGTCGGTGGAAGGAGGAGGAA
CasaMV1_newconsensus_sample311_3  CTTATGATACCAATTGGTTGCATTTCGGTAGGATTGCTCAGTCGGTGGAAGGAGGAGGAA
BK010438.1                        CTTATGATACCAATTGGTTACATTTCGGTAGGATCGCTCAGTCGGTGGAAGGAGGAGGAA
BK010436.1                        CTTATGATACCAATTGGTTACATTTCGGTAGGATTGCTCAGTCGGTGGAAGGAGGAGGAA
                                  ******** **  ****** ********* **** *********** *************

MT878083.1                        AGAGGCGTTTGTTTGGTATAGGAAACTATGTCAAACAACGTCTCCTCCAACCTGTGCATG
MT878082.1                        AGAGGCGTTTGTTTGGTATAGGAAACTATGTCAAACAACGTCTCCTCCAACCTGTGCATG
MT878081.1                        AGAGGCGTTTGTTTGGTATAGGAAACTATGTCAAACAACGTCTCCTCCAACCTGTGCATG
MT878080.1                        AGAGGCGTTTGTTTGGTATAGGAAACTATGTCAAACAACGTCTCCTCCAACCTGTGCATG
MT878084.1                        AACGACGTTTATTTGGCATAGGAAACTATGTTAAACAGCGTCTTCTCCAACCTGTGCATG
BK010428.1                        AACGACGTTTATTTGGCATAGGAAACTATGTTAAACAGCGTCTTCTCCAACCTGTGCATG
BK010437.1                        AACGACGTTTATTTGGCATAGGAAACTATGTTAAACAGCGTCTTCTCCAACCTGTGCATG
CasaMV1_newconsensus_sample311_3  AACGACGTTTATTTGGCATAGGAAACTATGTTAAACAGCGTCTTCTCCAACCTGTGCATG
BK010438.1                        AACGACGATTGTTTGGTATAGGAAACTATGTTAAACAACGTCTTCTCCAACCTGTACATG
BK010436.1                        AACGACGATTGTTTGGCATAGGAAACTATGTAAAACAACGTCTTCTCCAACCTGTGCATG
                                  *  * ** ** ***** ************** ***** ***** *********** ****

MT878083.1                        AGTGGGCCATGCGGGTTCTTGCCCGCATACCCATGGATGGGACATTTAACCAAAGTGCTC
MT878082.1                        AGTGGGCCATGCGGGTTCTTGCCCGCATACCCATGGATGGGACATTTAACCAAAGTGCTC
MT878081.1                        AGTGGGCCATGCGGGTTCTTGCCCGCATACCCATGGATGGGACATTTAACCAAAGTGCTC
MT878080.1                        AGTGGGCCATGCGGGTTCTTGCCCGCATACCCATGGATGGGACATTTAACCAAAGTGCTC
MT878084.1                        ATTGGGCTATGCAGGTTTTATCCCGCATACCCATGGATGGGACCTTTGATCAAAGTGCTC
BK010428.1                        ATTGGGCTATGCAGGTTTTATCCCGCATACCCATGGATGGGACCTTTGATCAAAGTGCTC
BK010437.1                        ATTGGGCTATGCAGGTTTTATCCCGCATACCCATGGATGGGACCTTTGATCAAAGTGCTC
CasaMV1_newconsensus_sample311_3  ATTGGGCTATGCAGGTTTTATCCCGCATACCCATGGATGGGACCTTTGATCAAAGTGCTC
BK010438.1                        ATTGGGCTATGCAGGTTTTATCCCGCATACCCATGGATGGGACCTTTGATCAAAGTGCTC
BK010436.1                        ATTGGGCTATGCAGGTGTTATCCCGCATACCCATGGATGGGACCTTTGATCAAAGTGCTC
                                  * ***** **** ***  *  ********************** *** * **********

MT878083.1                        CCATACATCGGTTAGCCAGACTCGAGACAACAGGTTTTATAGCCTCGTTCGATTTGAAAT
MT878082.1                        CCATACATCGGTTAGCCAGACTCGAGACAACAGGTTTTATAGCCTCGTTCGATTTGAAAT
MT878081.1                        CCATACATCGGTTAGCCAGACTCGAGACAACAGGTTTTATAGCCTCGTTCGATTTGAAAT
MT878080.1                        CCATACATCGGTTAGCCAGACTCGAGACAACAGGTTTTATAGCCTCGTTCGATTTGAAAT
MT878084.1                        CCATACATCGGTTAGCCAGACTAGAGACAACAGGCTTTATAGCTTCGTTCGATTTAAAAT
BK010428.1                        CCATACATCGGTTAGCCAGACTAGAGACAACAGGCTTTATAGCTTCGTTCGATTTAAAAT
BK010437.1                        CCATACATCGGTTAGCCAGACTAGAGACAACAGGCTTTATAGCTTCGTTCGATTTAAAAT
CasaMV1_newconsensus_sample311_3  CCATACATCGGTTAGCCAGACTAGAGACAACAGGCTTTATAGCTTCGTTCGATTTAAAAT
BK010438.1                        CCATACATCGGTTAGCCAGACTAGAGACAACAGGCTTTATAGCTTCGTTCGATTTAAAAT
BK010436.1                        CCATACATCGGTTAGCCAGACTTGAGACAACAGGCTTTATAGCTTCGTTTGATTTAAAAT
                                  ********************** *********** ******** ***** ***** ****

MT878083.1                        CTGCAACTGACCGGTGGCCAGTGTCTGTCATTCATGATGTATTTGCATTAATGTTTGGCC
MT878082.1                        CTGCAACTGACCGGTGGCCAGTGTCTGTCATTCATGATGTATTTGCATTAATGTTTGGCC
MT878081.1                        CTGCAACTGACCGGTGGCCAGTGTCTGTCATTCATGATGTATTTGCATTAATGTTTGGCC
MT878080.1                        CTGCAACTGACCGGTGGCCAGTGTCTGTCATTCATGATGTATTTGCATTAATGTTTGGCC
MT878084.1                        CTGCTACTGACCGATGGCCAGTTTCGGTTATTCATGATGTGTTCGCACTCATGTTCAGCC
BK010428.1                        CTGCTACTGACCGATGGCCAGTTTCGGTTATTCATGATGTGTTCGCACTCATGTTTAGCC
BK010437.1                        CTGCTACTGACCGATGGCCAGTTTCGGTTATTCATGATGTGTTCGCACTCATGTTTAGCC
CasaMV1_newconsensus_sample311_3  CTGCTACTGACCGATGGCCAGTTTCGGTTATTCATGATGTGTTCGCACTCATGTTTAGCC
BK010438.1                        CTGCTACTGACCGGTGGCCAGTTTCGGTTATTCATGATGTCTTTGCACTCATGTTTGGCC
BK010436.1                        CTGCTACTGACCGGTGGCCAGTTTCGGTGATTCATGATGTGTTCGCACTCATGTTTGGCC
                                  **** ******** ******** ** ** *********** ** *** * *****  ***

MT878083.1                        CGACTCTGGCCTCATGTGTGGTTAATGGTTGTTTAGCTCTTAACGTCTTTTCACTGGTTA
MT878082.1                        CGACTCTGGCCTCATGTGTGGTTAATGGTTGTTTAGCTCTTAACGTCTTTTCACTGGTTA
MT878081.1                        CGACTCTGGCCTCATGTGTGGTTAATGGTTGTTTAGCTCTTAACGTCTTTTCACTGGTTA
MT878080.1                        CGACTCTGGCCTCATGTGTGGTTAATGGTTGTTTAGCTCTTAACGTCTTTTCACTGGTTA
MT878084.1                        CAACATTGGCTTCATGTGTGGTCAATGGTTGTCTTGCTTTAAACGTCTTTTCGGTGTTGC
BK010428.1                        CAACATTGGCTTCATGTGTGGTCAATGGTTGTCTTGCTTTAAACGTCTTTTCGGTGTTGC
BK010437.1                        CAACATTGGCTTCATGTGTGGTCAATGGTTGTCTTGCTTTAAACGTCTTTTCGGTGTTGC
CasaMV1_newconsensus_sample311_3  CAACATTGGCTTCATGTGTGGTCAATGGTTGTCTTGCTTTAAACGTCTTTTCGGTGTTGC
BK010438.1                        CAACATTGGCTTCATGTGTGGTCAATGGTTGTCTTGCTCTCAACGTCTTTTCGGTGTTAC
BK010436.1                        CAACATTGGCTTCATGTGTGGTCAATGGTTGTCTTGCTTTAAATGTCTTTTCGGTGTTGC
                                  * **  **** *********** ********* * *** * ** ********  ** *

MT878083.1                        ATCCTATGGTTAGGAAACCGTCAAAAGTTTGTTTTAAGACTGGACAGCCTTTAGGCTACT
MT878082.1                        ATCCTATGGTTAGGAAACCGTCAAAAGTTTGTTTTAAGACTGGACAGCCTTTAGGCTACT
MT878081.1                        ATCCTATGGTTAGGAAACCGTCAAAAGTTTGTTTTAAGACTGGACAGCCTTTAGGCTACT
MT878080.1                        ATCCTATGGTTAGGAAACCGTCAAAAGTTTGTTTTAAGACTGGACAGCCTTTAGGCTACT
MT878084.1                        CTCCGTTTGTACGGAAGCCATCAAAAGTATGTTTTAAAACAGGCCAGCCATTAGGTTATT
BK010428.1                        CTCCGTTTGTACGGAAGCCATCAAAAGTATGTTTTAAAACAGGCCAGCCATTAGGTTATT
BK010437.1                        CTCCGTTTGTACGGAAGCCATCAAAAGTATGTTTTAAAACAGGCCAGCCATTAGGTTATT
CasaMV1_newconsensus_sample311_3  CTCCGTTTGTACGGAAGCCATCAAAAGTATGTTTTAAAACAGGCCAGCCATTAGGTTATT
BK010438.1                        CTCCGTTTGTTCGGAAGTCATCGAAAGTCTGTTTTAAGACTGGACAGCCATTAGGTTATT
BK010436.1                        CTCCGTTTGTTCGGAAGCCATCAAAAGTATGTTTTAAAACAGGGCAGCCATTAGGTTATT
                                   ***  * **  ****  * ** ***** ******** ** ** ***** ***** ** *

MT878083.1                        ATTCCTCTTGGCCGCTTTTTGCGCTTTCCCACCACTATATTGTGTGGTTGGCAGCAAAGC
MT878082.1                        ATTCCTCTTGGCCGCTTTTTGCGCTTTCCCACCACTATATTGTGTGGTTGGCAGCAAAGC
MT878081.1                        ATTCCTCTTGGCCGCTTTTTGCGCTTTCCCACCACTATATTGTGTGGTTGGCAGCAAAGC
MT878080.1                        ATTCCTCTTGGCCGCTTTTTGCGCTTTCCCACCACTATATTGTGTGGTTGGCAGCAAAGC
```

```
MT878084.1                          TATCCTCTTGGCCGTTGTTTGCGTTATCCCACCACTATATTGTGTGGTTGGCGGCAAAGC
BK010428.1                          TATCCTCTTGGCCGTTGTTTGCGTTATCCCACCACTATATTGTGTGGTTGGCGGCAAAGC
BK010437.1                          TATCCTCTTGGCCGTTGTTTGCGTTATCCCACCACTATATTGTGTGGTTGGCGGCAAAGC
CasaMV1_newconsensus_sample311_3    TATCCTCTTGGCCGTTGTTTGCGTTATCCCACCACTATATTGTGTGGTTGGCGGCAAAGC
BK010438.1                          TATCCTCTTGGCCGTTATTTGCGTTGTCACACCATTACATTGTGTGGTTGGCAGCAAAGC
BK010436.1                          TATCCTCTTGGCCGTTGTTTGCGTTGTCCCACCACTATATTGTGTGGTTGGCCGCAAAGC
                                      ************ * ****** * ** ***** ** ************** *******

MT878083.1                          AAGCCTATCCTAATCTGGATGTGTTCGACAGGTATGCTCTTCTTGGCGATGACATAGTCA
MT878082.1                          AAGCCTATCCTAATCTGGATGTGTTCGACAGGTATGCTCTTCTTGGCGATGACATAGTCA
MT878081.1                          AAGCCTATCCTAATCTGGATGTGTTCGACAGGTATGCTCTTCTTGGCGATGACATAGTCA
MT878080.1                          AAGCCTATCCTAATCTGGATGTGTTCGACAGGTATGCTCTTCTTGGCGATGACATAGTCA
MT878084.1                          AAGCTTATCCTAATCTGGATGTGTTCGACCGGTATGCCCTTCTGGGTGACGATATTGTCA
BK010428.1                          AAGCTTATCCTAATCTGGATGTGTTCGACCGGTATGCCCTTCTGGGTGACGATATCGTCA
BK010437.1                          AAGCTTATCCTAATCTGGATGTGTTCGACCGGTATGCCCTTCTGGGTGACGATATTGTCA
CasaMV1_newconsensus_sample311_3    AAGCTTATCCTAATCTGGATGTGTTCGACCGGTATGCCCTTCTGGGTGACGATATTGTCA
BK010438.1                          AAGCCTATCCTAATCTGGATGTGTTCGACCGGTATGCTCTTCTCGGTGACGATATCGTCA
BK010436.1                          AAGCTTATCCTAATCTGGATGTGTTCGACCGGTATGCTCTTCTGGGTGACGATATCGTCA
                                    **** ************************ ******* ***** ** ** ** ** ****

MT878083.1                          TTGCCGATCGAGCAGTTGCCGAACAGTACCGACAGATACTGGAAGAGCTTGGGGTGACCA
MT878082.1                          TTGCCGATCGAGCAGTTGCCGAACAGTACCGACAGATACTGGAAGAGCTTGGGGTGACCA
MT878081.1                          TTGCCGATCGAGCAGTTGCCGAACAGTACCGACAGATACTGGAAGAGCTTGGGGTGACCA
MT878080.1                          TTGCCGATCGAGCAGTTGCCGAACAGTACCGACAGATACTGGAAGAGCTTGGGGTGACCA
MT878084.1                          TTGCAGATAGGGCAGTGGCAGAACAGTATCGACAGATATTAGAAGAACTGGGGGTAACCA
BK010428.1                          TTGCAGATAGGGCAGTGGCAGAACAGTATCGACAGATATTAGAAGAACTGGGGGTAACCA
BK010437.1                          TTGCAGATAGGGCAGTGGCAGAACAGTATCGACAGATATTAGAAGAACTGGGGGTAACCA
CasaMV1_newconsensus_sample311_3    TTGCAGATAGGGCAGTGGCAGAACAGTATCGACAGATATTAGAAGAACTGGGGGTAACCA
BK010438.1                          TTGCGGATAGAGCAGTGGCTGAACAGTATCGACAGATACTAGAGGAATTAGGGGTAACCA
BK010436.1                          TTGCAGATAGAGCAGTGGCTGAACAGTATCGACAGATACTAGTAGAATTAGGGGTGACCA
                                    **** *** * ***** ** ******** ********* * *  **  * ***** ****



MT878083.1                          TATCATTTTCAAAATCGATTATAAGTGATTATGGTTGTCTTGAGTTCGCGAAGCAGTTCT
MT878082.1                          TATCATTTTCAAAATCGATTATAAGTGATTATGGTTGTCTTGAGTTCGCAAAGCAGTTCT
MT878081.1                          TATCATTTTCAAAATCGATTATAAGTGATTATGGTTGTCTTGAGTTCGCGAAGCAGTTCT
MT878080.1                          TATCATTTTCAAAATCGATTATAAGTGATTATGGTTGTCTTGAGTTCGCGAAGCAGTTCT
MT878084.1                          TATCATTCTCAAAATCAATTGTGAGTGATTATGGTTGCCTTGAGTTCGCGAAGCAGTTTT
BK010428.1                          TATCATTCTCAAAATCAATTGTGAGTGATTATGGTTGCCTTGAGTTCGCGAAGCAGTTTT
BK010437.1                          TATCATTCTCAAAATCAATTGTGAGTGATTATGGTTGCCTTGAGTTCGCGAAGCAGTTTT
CasaMV1_newconsensus_sample311_3    TATCATTCTCAAAATCAATTGTGAGTGATTATGGTTGCCTTGAGTTCGCGAAGCAGTTTT
BK010438.1                          TATCATTCTCAAAATCTATTGTGAGTGATTATGGTTGTCTTGAATTCGCGAAGCAGTTTT
BK010436.1                          TATCATTCTCAAAATCTATTGTGAGTGATTTTGGTTGTCTTGAGTTCGCGAAGCAGTTCT
                                    ******* ******** *** * ******* ****** ***** ***** ******** *

MT878083.1                          GGGTGAAAGACTTAAAAGTGAATCTTTCACCTGTATCTGCACCTTCTGTTCTAGTATCGA
MT878082.1                          GGGTGAAAGACTTAAAAGTGAATCTTTCACCTGTATCTGCACCTTCTGTTCTAGTATCGA
MT878081.1                          GGGTGAAAGACTTAAAAGTGAATCTTTCACCTGTATCTGCACCTTCTGTTCTAGTATCGA
MT878080.1                          GGGTGAAAGACTTAAAAGTGAATCTTTCACCTGTATCTGCACCTTCTGTTCTAGTATCGA
MT878084.1                          GGGTAAAAGACATAAAAGTGAATCTTTCACCCGTGTCTGCTCCTTCTGTTCTAGTATCGA
BK010428.1                          GGGTAAAAGACATAAAAGTGAATCTTTCACCCGTATCTGCTCCTTCTGTTCTAGTATCGA
BK010437.1                          GGGTAAAAGACATAAAAGTGAATCTTTCACCCGTATCTGCTCCTTCTGTTCTAGTATCGA
CasaMV1_newconsensus_sample311_3    GGGTGAAAGACATAAAAGTGAATCTTTCACCCGTATCTGCTCCTTCTGTTCTAGTATCGA
BK010438.1                          GGGTAAAAGACATAAAAGTGAATCTTTCACCTATATCTGCACCTTCAGTTCTAGTATCGA
BK010436.1                          GGGTGAAAGACATAAAAGTGAATCTTTCACCTGTATCTGCACCTTCAGTACTAGTATCGA
                                    **** ****** *******************  * ***** ***** ** **********

MT878083.1                          GGTCAATCATGGGGATTACCCAGTTAGCGGATAAATATGAGGTTAATTCAGCCTCAGGTT
MT878082.1                          GGTCAATCATGGGGATTACCCAGTTAGCGGATAAATATGAGGTTAATTCAGCCTCAGGTT
MT878081.1                          GGTCAATCATGGGGATTACCCAGTTAGCGGATAAATATGAGGTTAATTCAGCCTCAGGTT
MT878080.1                          GGTCAATCATGGGGATTACCCAGTTAGCGGATAAATATGAGGTTAATTCAGCCTCAGGTT
MT878084.1                          GAACGGTCATGGGAATCACTCAGTTGGCTGATAAGTATGAGGTTAAATCAGCCTCAGGCT
BK010428.1                          GAACGGTCATGGGAATCACTCAGTTGGCTGATAAGTATGAGGTTAAATCAGCCTCAGGCT
BK010437.1                          GAACGGTCATGGGAATCACTCAGTTGGCTGATAAGTATGAGGTTAAATCAGCCTCAGGCT
CasaMV1_newconsensus_sample311_3    GAACGGTCATGGGAATCACTCAGTTGGCTGATAAGTATGAGGTTAAATCAGCCTCAGGCT
BK010438.1                          GAACGATCATGGGTATCACCCAGCTGGCAGATAAGTATGAGGTTAATTCAGCCTCAGGCT
BK010436.1                          GGACGATTATGGGAATCACCCAGCTCGCTGATAAGTATGAGGTTAATTCAGCCTCAGGCT
                                    *  *  * ***** ** ** *** * ** ***** *********** *********** *

MT878083.1                          TACTTCGTTTAGCTGGAGCTGGTTTCCGTGTGAGATCTCAGATGATGTCTCTCAAGTTAG
MT878082.1                          TACTTCGTTTAGCTGGAGCTGGTTTCCGTGTGAGATCTCAGATGATGTCTCTTAAGTTAG
MT878081.1                          TACTTCGTTTAGCTGGAGCTGGTTTCCGTGTGAGATCTCAGATGATGTCTCTTAAGTTAG
MT878080.1                          TACTTCGTTTAGCTGGAGCTGGTTTCCGTGTGAGATCTCAGATGATGTCTCTTAAGTTAG
MT878084.1                          TATTACGCCTTGCTGGTGCTGGTTTCCGTGTTCGATCTCAGATGATGTCTCTCAAGTTAG
BK010428.1                          TATTACGCCTTGCTGGTGCTGGTTTCCGTGTTCGATCTCAGATGATGTCTCTCAAGTTAG
BK010437.1                          TATTACGCCTTGCTGGTGCTGGTTTCCGTGTTCGATCTCAGATGATGTCTCTCAAGTTAG
CasaMV1_newconsensus_sample311_3    TATTACGCCTTGCTGGTGCTGGTTTCCGTGTTCGATCTCAGATGATGTCTCTCAAGTTAG
BK010438.1                          TATTACGTTTAGCTGGTGCTGGTTTCCGTGTTCGCTCTCAGATGATGTCTCTTAAGTTAG
BK010436.1                          TATTACGTCTTGCTGGTGCTGGTTTCCGAGTCCGTTCTCAGATGATGTCTCTCAAGTTAG
                                    ** * **  * ***** *********** **  * ***************** *******

MT878083.1                          CTGCAAGGTGGAGACGTCTTCTTGTCGTGGCTGGGAAGCCACCTAAGAAGTTCGAATCAG
MT878082.1                          CTGCAAGGTGGAGACGTCTTCTTGTCGTGGCTGGGAAGCCACCTAAGAAGTTCGAATCAG
MT878081.1                          CTGCAAGGTGGAGACGTCTTCTTGTCGTGGCTGGGAAGCCACCTAAGAAGTTCGAATCAG
MT878080.1                          CTGCAAGGTGGAGACGTCTTCTTGTCGTGGCTGGGAAGCCACCTAAGAAGTTCGAATCAG
MT878084.1                          CTTCAAAGTGGAGACGTCTTCTTGTTGTGGCTAGTAAGCCGCCGAAGAAGTTCGAATCAG
BK010428.1                          CTTCAAAGTGGAGACGTCTTCTTGTTGTGGCTAGTAAGCCGCCGAAGAAGTTCGAATCAG
BK010437.1                          CTTCAAAGTGGAGACGTCTTCTTGTTGTGGCTAGTAAGCCGCCGAAGAAGTTCGAATCAG
CasaMV1_newconsensus_sample311_3    CTTCAAAGTGGAGACGTCTTCTTGTTGTGGCTAGTAAGCCGCCGAAGAAGTTCGAATCAG
BK010438.1                          CTGCAAAGTGGAGACGTCTTCTTGTTGTGGCTAGTAAGCCTCCCAAGAAATTCGAATCAG
BK010436.1                          CATCAAAGTGGAGACGTCTTCTTGTTGTGGCTAGTAAGCCTCCCAAGAAATTCGAATCAG
                                    *  *** ****************** ****** * ***** ** ***** **********

MT878083.1                          CCTTACATTGGTGGGTCGCAGGGGGTAAACCCCTCAATCCCTATGTGAGGGCGATAATGG
MT878082.1                          CCTTACATTGGTGGGTTGCAGGGGGTAAACCCCTCAATCCCTATGTGAGGGCGATAATGG
MT878081.1                          CCTTACATTGGTGGGTTGCAGGGGGTAAACCCCTCAATCCCTATGTGAGGGCGATAATGG
MT878080.1                          CCTTACATTGGTGGGTTGCAGGGGGTAAACCCCTCAATCCCTATGTGAGGGCGATAATGG
MT878084.1                          CTTTGCATTGGTGGGTTGCAGGAGGTAAACCTCTCAATCCTTATGTAAAGGCGATAATGG
BK010428.1                          CTTTGCATTGGTGGGTTGCAGGAGGTAAACCTCTCAATCCTTATGTGAAGGCGATAATGG
BK010437.1                          CTTTGCATTGGTGGGTTGCAGGAGGTAAACCTCTCAATCCTTATGTGAAGGCGATAATGG
CasaMV1_newconsensus_sample311_3    CTTTGCATTGGTGGGTTGCAGGAGGTAAACCTCTCAATCCTTATGTGAAGGCGATAATGG
BK010438.1                          CTTTACATTGGTGGGTTGCAGGGGGTAAACCTCTCAATCCTTATGTGAAGGCGATAATGG
BK010436.1                          CTTTGCATTGGTGGGTTGCTGGGGGTAAACCTCTCAATCCATATGTGAAGGCGATAATGA
                                    * ** *********** ** ** ******** ******** ***** * **********

MT878083.1                          TTCGTCTGTTCATTACTCAGTTGAAGCCTAAAGAACCTCAACTGCCTATGAACCGCATGT
MT878082.1                          TTCGTCTGTTCATTACTCAGTTGAAGCCTAAAGAACCTCAACTGCCTATGAACCGCATGT
MT878081.1                          TTCGTCTGTTCATTACTCAGTTGAAGCCTAAAGAACCTCAACTGCCTATGAACCGCATGT
MT878080.1                          TTCGTCTGTTCATTACTCAGTTGAAGCCTAAAGAACCTCAACTGCCTATGAACCGCATGT
MT878084.1                          TTCGTCTGTTCATTAGTCAACTGAAGCCTAAAGAACCTCAGTTGCCTATGAACCGCATGT
BK010428.1                          TTCGTCTGTTCATTAGTCAACTGAAGCCTAAAGAACCTCAGTTGCCTATGAACCGCATGT
BK010437.1                          TTCGTCTGTTCATTAGTCAACTGAAGCCTAAAGAACCTCAGTTGCCTATGAACCGCATGT
CasaMV1_newconsensus_sample311_3    TTCGTCTGTTCATTAGTCAACTGAAGCCTAAAGAACCTCAGTTGCCTATGAACCGCATGT
BK010438.1                          TTCGTCTGTTCATTAGTCAACTGAAGCCTAAAGAACCTCAGTTGCCTATGAATCGCATGT
BK010436.1                          TTCGTCTGTTCATTAGCCAACTGAAGCCTAAAGAACCTCAGTTGCCTATGGACCGCATGT
                                    ***************  **  *******************  ******** * *******

MT878083.1                          TCGATGGGGAGGCGGCAATGTCAGAGTATACCCTGACTCGTAATTGGGTTTCCCAATGGC
MT878082.1                          TCGATGGGGAGGCGGCAATGTCAGAGTATACCCTGACTCGTAATTGGGTTTCCCAATGGC
MT878081.1                          TCGATGGGGAGGCGGCAATGTCAGAGTATACCCTGACTCGTAATTGGGTTTCCCAATGGC
MT878080.1                          TCGATGGGGAGGCGGCAATGTCAGAGTATACCCTGACTCGTAATTGGGTTTCCCAATGGC
```

```
MT878084.1                         TCGAGGGTGAAGCGACAGTATCTGAATATATGCTTACGCGCAATTGGGTTTCCCAATGGC
BK010428.1                         TCGAGGGTGAAGCGACAGTATCTGAATATATGCTTACGCGCAATTGGGTTTCCCAATGGC
BK010437.1                         TCGAGGGTGAAGCGACAGTATCTGAATATATGCTTACGCGCAATTGGGTTTCCCAATGGC
CasaMV1_newconsensus_sample311_3   TCGAGGGTGAAGCGACAGTATCTGAATATATGCTTACGCGCAATTGGGTTTCCCAATGGC
BK010438.1                         TCGAGGGTGAAGCAACAGTATCTGAGTATATGCTTACTCGTAATTGGGTTTCCCAATGGC
BK010436.1                         TCGAGGGTGAAGCAACAGTATCAGAATACATGCTTACTCGTAATTGGGTTTCCCAATGGC
                                   **** ** ** **  ** * ** ** ** *  ** ** ** *******************

MT878083.1                         TTCAGTGGTTATCATGGTACTGTCGTGTTCCTCTAGGTCCAGATCCAACAATATCTGCAT
MT878082.1                         TTCAGTGGTTATCATGGTACTGTCGTGTTCCTCTAGGTCCAGATCCAACAATATCTGCAT
MT878081.1                         TTCAGTGGTTATCATGGTACTGTCGTGTTCCTCTAGGTCCAGATCCAACAATATCTGCAT
MT878080.1                         TTCAGTGGTTATCATGGTACTGTCGTGTTCCTCTAGGTCCAGATCCAACAATATCTGCAT
MT878084.1                         TAAAGTGGTTATCTTGGTATTGTCGTGTAGCTCTTGGTCCAGATCCAACAATATCTGCAT
BK010428.1                         TAAAGTGGTTATCTTGGTATTGTCGTGTAGCTCTTGGTCCAGATCCAACAATATCTGCAT
BK010437.1                         TAAAGTGGTTATCTTGGTATTGTCGTGTAGCTCTTGGTCCAGATCCAACAATATCTGCAT
CasaMV1_newconsensus_sample311_3   TAAAGTGGTTATCTTGGTATTGTCGTGTAGCTCTTGGTCCAGATCCAACAATATCTGCAT
BK010438.1                         TTAAGTGGTTATCTTGGTATTGTCGCGTAGCCCTCGGTCCAGATCCAACATTAACTGCAT
BK010436.1                         TTAAGTGGTTGTCTTGGTATTGTCGTGTAGCTCTCGGTCCAGATCCAACAATATCTGCAT
                                   *  ******* ** ***** ***** **  * ** *************** ** ******

MT878083.1                         TGCTGGATGCTCCTGTAGTGGCGTCAAAATGGCAGCGCTCAACTTTCGATGCTCGCATCA
MT878082.1                         TGCTGGATGCTCCTGTAGTGGCGTCAAAATGGCAGCGCTCAACTTTCGATGCTCGCATCA
MT878081.1                         TGCTGGATGCTCCTGTAGTGGCGTCAAAATGGCAGCGCTCAACTTTCGATGCTCGCATCA
MT878080.1                         TGCTGGATGCTCCTGTAGTGGCGTCAAAATGGCAGCGCTCAACTTTCGATGCTCGCATCA
MT878084.1                         TGCTGGATGCTCCTGTAGTGGCATCAAAATGGCAGCGCTCAACTTTTGATATTACTATCA
BK010428.1                         TGCTGGATGCTCCTGTAGTGGCATCAAAATGGCAGCGCTCAACTTTTGATATTACTATCA
BK010437.1                         TGCTGGATGCTCCTGTAGTGGCATCAAAATGGCAGCGCTCAACTTTTGATATTACTATCA
CasaMV1_newconsensus_sample311_3   TGCTGGATGCTCCTGTAGTGGCATCAAAATGGCAGCGCTCAACTTTTGATATTACTATCA
BK010438.1                         TGCTGGATGCTCCCGTAGTGGCATCAAAATGGCAGCGCTCAACTTTTGATATTACTATCA
BK010436.1                         TGCTGGATGCTCCCGTAGTGGCATCAAAATGGCAGCGCTCAACTTTCGATATTACTATCA
                                   ************* ******** *********************** ***  *   ****

MT878083.1                         AAAAGTTTGGGTTGTTATGGAGATGTTACGATATAGGAATTCAGTACGCAGTCTCTTGGT
MT878082.1                         AAAAGTTTGGGTTGTTATGGAGATGTTACGATATAGGAATTCAGTACGCAGTCTCTTGGT
MT878081.1                         AAAAGTTTGGGTTGTTATGGAGATGTTACGATATAGGAATTCAGTACGCAGTCTCTTGGT
MT878080.1                         AAAAGTTTGGGTTGTTATGGAGATGTTACGATATAGGAATTCAGTACGCAGTCTCTTGGT
MT878084.1                         AGAAGTTTGGGTTGCTATGGAGGTGTTACGATATAGGAATTCAGTACACTGTTGATTGGT
BK010428.1                         AGAAGTTTGGGTTGCTATGGAGGTGTTACGATATAGGAATTCAGTACACTGTTGATTGGT
BK010437.1                         AGAAGTTTGGGTTGCTATGGAGGTGTTACGATATAGGAATTCAGTACACTGTTGATTGGT
CasaMV1_newconsensus_sample311_3   AGAAGTTTGGGTTGCTATGGAGGTGTTACGATATAGGAATTCAGTACACTGTTGATTGGT
BK010438.1                         AAAAGTTCGGGTTGTTATGGAGATGCTACGATATAGGAATTCAGTACGCTATTGATTGGT
BK010436.1                         AAAAGTTTGGGTTGTTGTGGAGATGTTACGATATAGGAATTCAGTACGCTGTTGATTGGT
                                   * ***** ****** * ***** ** ******************** *  *   *****

MT878083.1                         ATCCATTAGTTTTGGAACCAAGGATTGTGGGTCTCTTGCCGTGTTGGTACGTTTTAACAT
MT878082.1                         ATCCATTAGTTTTGGAACCAAGGATTGTGGGTCTCTTGCCGTGTTGGTACGTTTTAACAT
MT878081.1                         ATCCATTAGTTTTGGAACCAAGGATTGTGGGTCTCTTGCCGTGTTGGTACGTTTTAACAT
MT878080.1                         ATCCATTAGTTTTGGAACCAAGGATTGTGGGTCTCTTGCCGTGTTGGTACGTTTTAACAT
MT878084.1                         ATCCAGGAGTCCTGGAACCGTTAATAGTGGGTCTCTTACCGTGTTGGTATAATTTGAGTT
BK010428.1                         ATCCAGGAGTCCTGGAACCGTTAATAGTGGGTCTCTTACCGTGTTGGTATAATTTGAGTT
BK010437.1                         ATCCAGGAGTCCTGGAACCGTTAATAGTGGGTCTCTTACCGTGTTGGTATAATTTGAGTT
CasaMV1_newconsensus_sample311_3   ATCCAGGAGTCCTGGAACCGTTAATAGTGGGTCTCTTACCGTGTTGGTATAATTTGAGTT
BK010438.1                         ATCCAGGAGTCCTGGAACCGTTGATAGTGGGTCCCTTACCGTGTTGGTACAATTTGAGTA
BK010436.1                         ATCCAGGAGTCCTGGAACCGTTAATAGTGGGTCCCTTACCGTGTTGGTACAACGTGAGTT
                                   *****  ***  *******    ** ******* *** ***********      * *

MT878083.1                         ATCAACCGGTCGGATATTCTGTATCTATTGTTGATGGTATTGGCTATGTATCGCACCATA
MT878082.1                         ATCAACCGGTCGGATATTCTGTATCTATTGTTGATGGTATTGGCTATGTATCGCACCATA
MT878081.1                         ATCAACCGGTCGGATATTCTGTATCTATTGTTGATGGTATTGGCTATGTATCGCACCATA
MT878080.1                         ATCAACCGGTCGGATATTCTGTATCTATTGTTGATGGTATTGGCTATGTATCGCACCATA
MT878084.1                         ACCAACCTGTAGGATATTCTGTATCTATCGTTGATGGTATTGGCTATGTATCGCACCATA
BK010428.1                         ACCAACCTGTAGGATATTCTGTATCTATTGTTGATGGTATTGGCTATGTATCGCACCATA
BK010437.1                         ACCAACCTGTAGGATATTCTGTATCTATTGTTGATGGTATTGGCTATGTATCGCACCATA
CasaMV1_newconsensus_sample311_3   ACCAACCTGTAGGATATTCTGTATCTATCGTTGATGGTATTGGCTATGTATCGCACCATA
BK010438.1                         TCCAACCTGTAGGGTATTCTGTATCTATTGTTGATGGTATTGGTTATGTATCGCACCATA
BK010436.1                         CCCAACCAGTAGGGTATTCTGTATCTATTGTTGATGGTATTGGCTATGTATCGCACCATA
                                     ***** ** ** ************** ************* ***************

MT878083.1                         GTGTCGCTGTTTATCTAATCAGTAAACAGTGAGTAAATAGGGTGACTGACATATAAGAAT
MT878082.1                         GTGTCGCTGTTTATCTAATCAGTAAACAGTGAGTAAATAGGGTGACTGACATATAAGAAT
MT878081.1                         GTGTCGCTGTTTATCTAATCAGTAAACAGTGAGTAAATAGGGTGACTGACATATAAGAAT
MT878080.1                         GTGTCGCTGTTTATCTAATCAGTAAACAGTGAGTAAATAGGGTGACTGACATATAAGAAT
MT878084.1                         GTGTCGCTGTTTACCTAATCAGTAAACAGTGAGTAAATAGGGTGACTG-CATGTAAGAAA
BK010428.1                         GTGTCGCTGTTTACCTAATCAGTAAACAGTGAGTAAATAGGGTGACTG-CATGTAAGAAA
BK010437.1                         GTGTCGCTGTTTACCTAATCAGTAAACAGTGAGTAAATAGGGTGACTG-CATGTAAGAAA
CasaMV1_newconsensus_sample311_3   GTGTCGCTGTTTACCTAATCAGTAAACAGTGAGTAAATAGGGTGACTG-CATGTAAGAAA
BK010438.1                         GTGTCACTGTCTACCTAATCAGTAAACAGTGAGTAAATAGGGTGACTG-CATGTAAGAAA
BK010436.1                         GTGTCACTGTTTACCTAATCAGTAAACAGTGAGTAAATAGGGTGACTG-CATGTAAGAAA
                                   ***** **** ** ********************************** *** ******

MT878083.1                         TTAGGTACATCTGAGTACATAGTATCCTGTACAAGTACTCTTCTTCGGAAGGGGGGGTGT
MT878082.1                         TTAGGTACATCTGAGTACATAGTATCCTGTACAAGTACTCTTCTTCGGAAGGGGGGGTGT
MT878081.1                         TTAGGTACATCTGAGTACATAGTATCCTGTACAAGTACTCTTCTTCGGAAGGGGGGGTGT
MT878080.1                         TTAGGTACATCTGAGTACATAGTATCCTGTACAAGTACTCTTCTTCGGAAGGGGGGGTGT
MT878084.1                         TTAGGTACATCTGAGTACATAGTATCCTGTACAAGTACTCTTCTTCGGAAGGGGGGGTGT
BK010428.1                         TTAGGTACATCTGAGTACATAGTATCCTGTACAAGTACTCTTCTTCGGAAGGGGGGGTGT
BK010437.1                         TTAGGTACATCTGAGTACATAGTATCCTGTACAAGTACTCTTCTTCGGAAGGGGGGGTGT
CasaMV1_newconsensus_sample311_3   TTAGGTACATCTGAGTACATAGTATCCTGTACAAGTACTCTTCTTCGGAAGGGGGGGTGT
BK010438.1                         TTAGGTACATCTGAGTACATAGTATCCTGTACAAGTACTCTTCTTCGGAAGGGGGGGTGT
BK010436.1                         TTAGGTACATCTGAGTACATAGTATCCTGTACAAGTACTCTTCTTCGGAAGGGGGGGTGT
                                   ************************************************************

MT878083.1                         GCAGGATCATAGGAACC-----------------------
MT878082.1                         GCAGGATCATAGGAACCGA---------------------
MT878081.1                         GCAGGATCATAGGAACCG----------------------
MT878080.1                         GCAGGATCATAGGAACCG----------------------
MT878084.1                         GCAGGATCATAGGAACCGA---------------------
BK010428.1                         GCAGGATCATAGGAACCGAATGATTAATCGGTTCCTAAACC
BK010437.1                         GCAGGATCATAGGAACCGAA--------------------
CasaMV1_newconsensus_sample311_3   GCAGGATCATAGGAACCGAATGATTAATCGGTTCCTAAACC
BK010438.1                         GCAGG-----------------------------------
BK010436.1                         GCAGGATCATAGGAACCG----------------------
                                   *****
```
