# Supplementary material for: Cannabis Virome Reconstruction and Antiviral RNAi Characterization through Small RNA Sequencing
Source: Plants (Basel). 2023 Nov 21;12(23):3925. doi: 10.3390/plants12233925 (PMC10707731; doi:10.3390/plants12233925)
Supplement: Supplementary file 1 [file plants-12-03925-s001.zip › Suppementary Figures S1-S4.pdf]

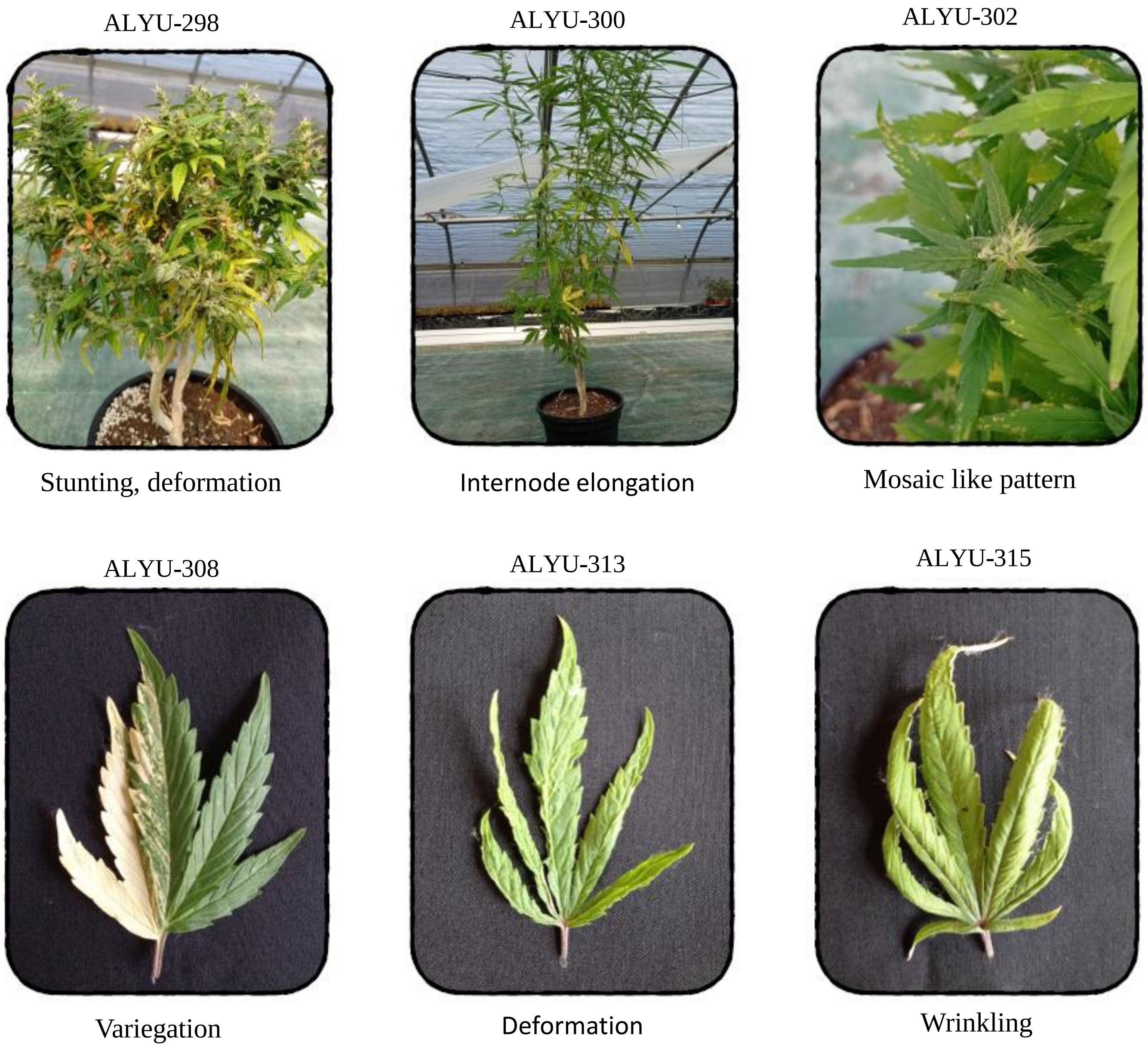

Figure S1. Examples of virus-like and other symptoms of hemp plants. Sample names are indicated above, while symptoms are named below each image.

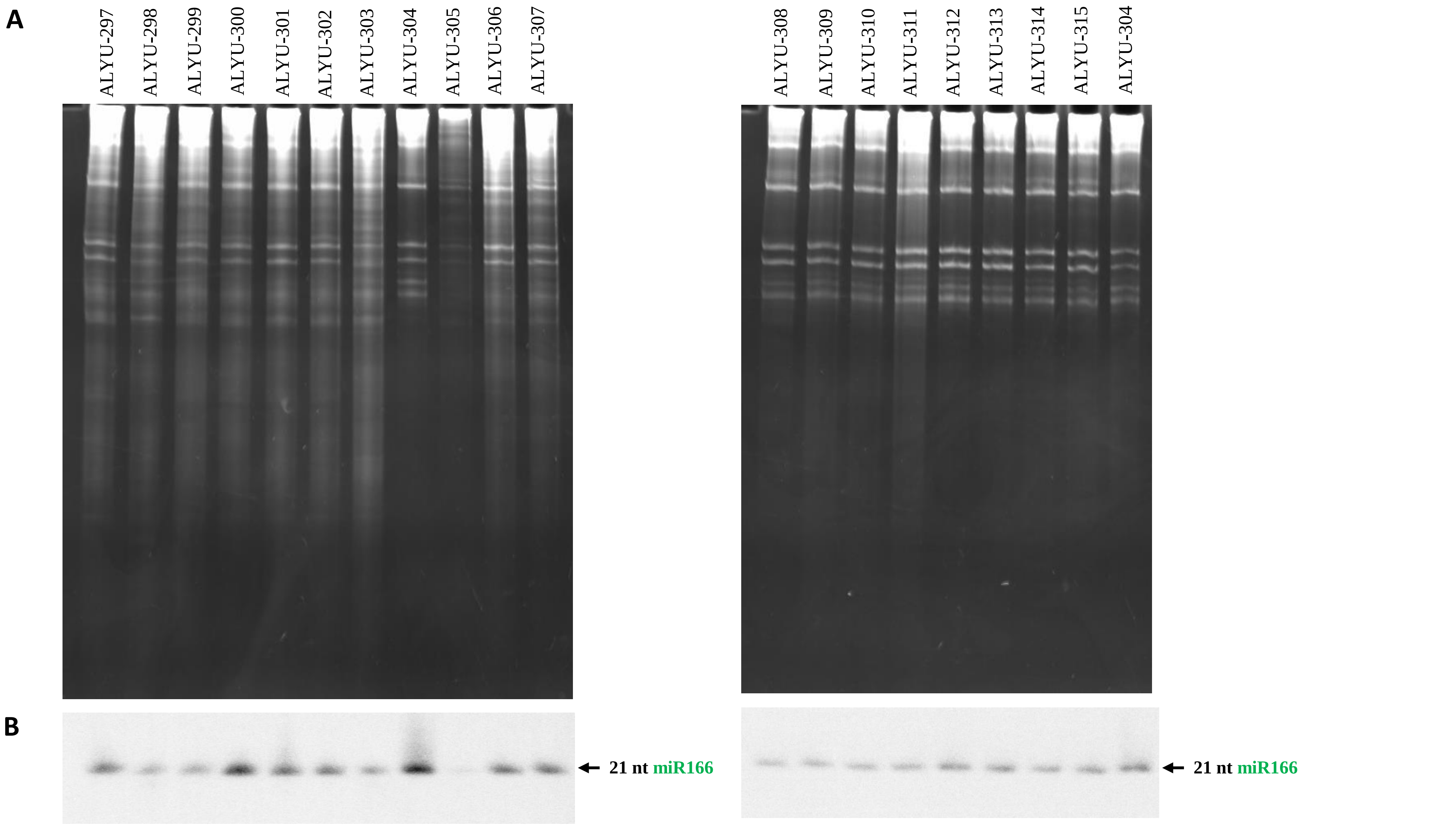

Figure S2. Small RNA blot hybridization analysis of total RNA from hemp plants. Total RNA was extracted from 19 leaf samples (ALYU-297-315) of hep plants using CTAB-LiCl protocol, separated on 15% polyacrylamide gel, stained with ethidium bromide (EtBr) (**A**), blotted onto a nylon N+ membrane, UV-crosslinked to the membrane and then hybridized with P32-labelled DNA oligonucleotide probes specific to the evolutionarily conserved plant miRNA (21 nt miR166; the probe miR166a_as 5'-GGGGAATGAAGCCTGGTCCGA). The membrane was washed and exposed on a phosphor screen and scanned (**B**). Position of 21 nt plant miR166 is indicated. Note that one of the samples (ALYU-304) was loaded on both gels.

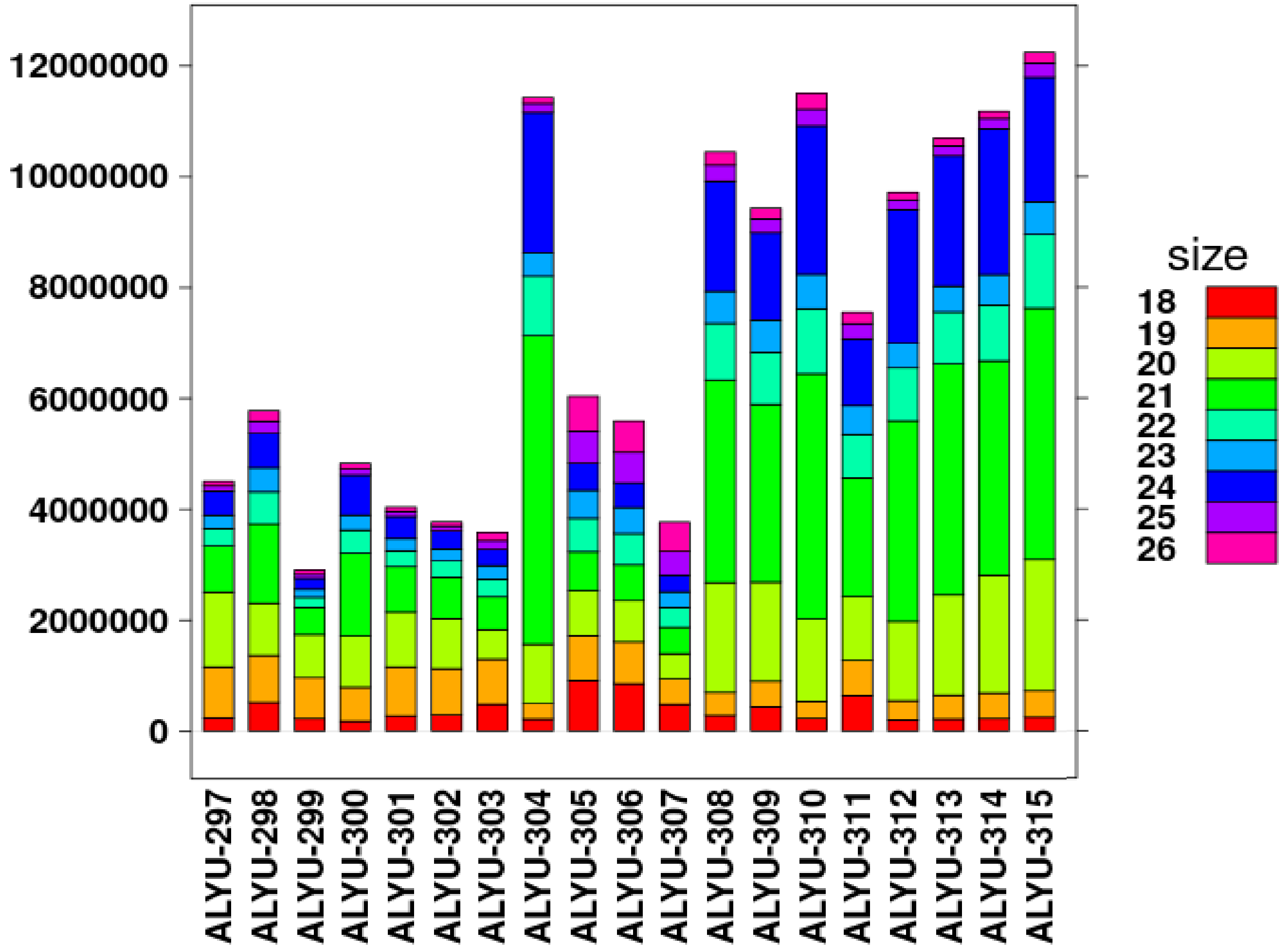

Figure S3. Size profiles of Illumina small RNA reads for 19 leaf samples (ALYU-297-315) of hemp plants. Illumina sRNA reads were sorted by size and those in the size range from 18 to 26 nts were counted. The resulting counts were plotted as stacked bar graphs, with size-classes color-coded.

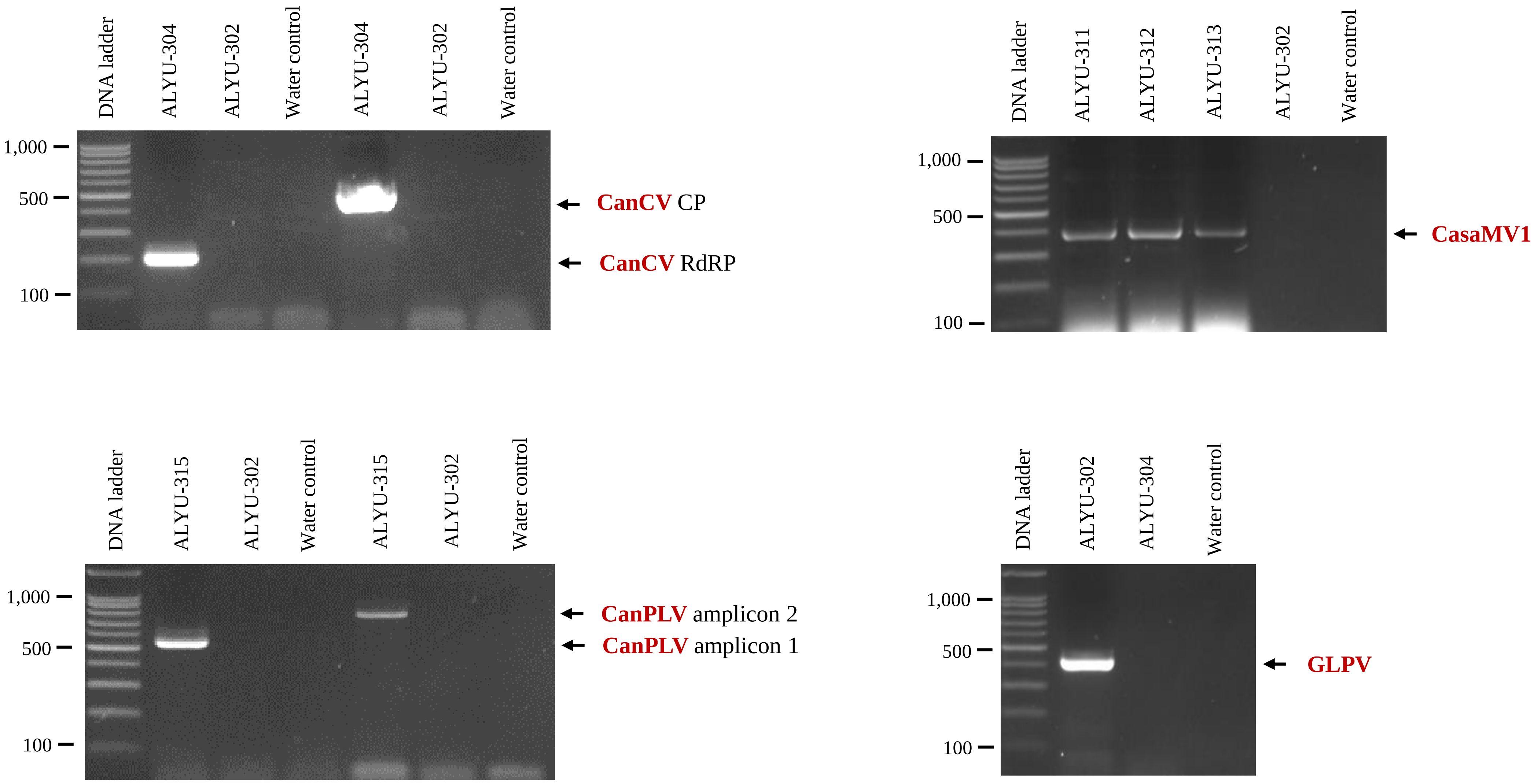

Figure S4. RT-PCR analysis of total RNA extracted from Cannabis leaf samples. Total RNA samples (ALYU-302, ALYU-304, ALYU-311, ALYU-312, ALYU-313, ALYU-315) were used for RT-PCR analysis with primer pairs specific for CanCV, CanPLV, CasaMV1 and CPLV (see Table S1 for primer sequences and amplicon sizes) as described in Materials and Methods. Representative pictures of 1% agarose gels stained with ethidium bromide are shown with the PCR products of expected sizes indicated by arrows for each virus and, in some cases, viral genome segments (CanCV) or different amplicons of the same genomic RNA (CanPLV). DNA ladder 100 bp plus (PanReac AppliChem, Turkey) was used.
